# Supplementary material for: Zero-field J-spectroscopy of quadrupolar nuclei
Source: Nat Commun. 2024 May 27;15:4487. doi: 10.1038/s41467-024-48390-2 (PMC11637023; doi:10.1038/s41467-024-48390-2)
Supplement: Supplementary file 1 — Supplementary Information [file 41467_2024_48390_MOESM1_ESM.pdf]

# *Supporting Information:*
# *Zero-Field J-spectroscopy of Quadrupolar Nuclei*


Román Picazo-Frutos,[a,b] Kirill F. Sheberstov,[a,b,c] John W. Blanchard,[a,b,d] Erik Van Dyke,[a,b]

Moritz Reh,[e,f] Tobias Sjoelander,[g,h,i] Alexander Pines,[h,i]

Dmitry Budker,[a,b,e] and Danila A. Barskiy*,[a,b,h,i]

[a]Helmholtz-Institut Mainz, GSI Helmholtzzentrum für Schwerionenforschung GmbH, 55128 Mainz, Germany

[b]Johannes Gutenberg-Universitat Mainz, 55128 Mainz, Germany

[c]Department of Chemistry, École Normale Supérieure, PSL University, Paris, France

[d]Quantum Technology Center, University of Maryland, College Park, MD, USA

[e]Department of Physics, University of California – Berkeley, Berkeley, California 94720, USA

[f]Kirchhoff-Institut für Physik, Universität Heidelberg, Im Neuenheimer Feld 227, 69120 Heidelberg, Germany

[g]Department of Physics, University of Basel, Klingelbergstrasse 82, Basel, CH-4056, Switzerland

[h]Department of Chemistry, University of California - Berkeley, California 94720-3220, USA

[i]Materials Science Division, Lawrence Berkeley National Laboratory, Berkeley, California 94720-3220, USA


## Table of Contents


A. Analysis procedure for extracting *J*-coupling ratio .......... 2

a. Systematic error in the analysis procedure .......... 7

B. Pulse-length dependence .......... 9

C. $T_1$ measurement of 15N ammonia .......... 11

D. Analytical calculations .......... 11

a. Zero-field NMR spectrum of $^{15}NH_4^+$ .......... 12

b. Zero-field NMR spectrum of $^{15}NDH_3^+$ .......... 13

c. Zero-field NMR spectrum of $^{15}ND_2H_2^+$ .......... 15

d. Zero-field NMR spectrum of $^{15}ND_3H^+$ .......... 18

e. Zero-field NMR spectrum of $^{15}ND_4^+$ .......... 19
E. Analysis and simulations programs .......... 21
a. Python code to eliminate baseline from time-domain signals .......... 21
b. Python code to construct and export the different partitions .......... 25
c. J-couplings ratio analysis of quadrupolar nuclei .......... 30
d. Simulation example of $^{15}NH_3$ .......... 30
References .......... 30

# A. Analysis procedure for extracting *J*-coupling ratio

All of the scans of the [$^{15}$N]- and [$^{14}$N] ammonium mixture (**Figure 1**) were subject to the same data analysis. Two commercially available magnetometers (QuSpin QZFM Gen-2; 4×4×4 mm$^3$, Rb vapor cell) were used to detect the signal in a gradiometer configuration. The difference between the two channels of the sensitive axis was taken as a gradiometric output signal, thus, enhancing the signal (by a factor ≈1.5 determined by the geometry of the sample and the exact location of the sensors) while simultaneously removing any common-mode noise. The experimental setup details can be found elsewhere.[1]

The data were acquired at a sampling frequency of 50 kHz and later downsized to 1 kHz (to follow the Nyquist condition to measure frequencies of up to 500 Hz) by an averaging-down procedure which removed high-frequency noise.[1] The NMR spectra were obtained from the time-domain signals via the usual Fourier transformation used in conventional high-field NMR. An extra step is added by scaling up by a factor of two every point of the time-domain signal except the initial point to get rid of baseline-offset artifacts, described recently in Ref. [2]. The data were zero-filled by the same amount of data points $t_{\mathrm{acq}}$ (zero-filling factor = 2) and no line broadening was added.

The resulting spectra exhibit a non-flat baseline attributed to a significant DC-offset component (observed at 0 Hz and not fully removed by the gradiometric detection, see below) and low-frequency fluctuations in the time-domain signal stemming from the temperature-lock PID readjustment of the optically pumped magnetometer (OPM) following the excitation pulse. This baseline profile poses challenges when fitting *J*-coupling frequencies. To address this issue, several steps have been implemented; here, we outline all of them:

(i) Subtraction of the two gradiometer channels of the two OPMs to enhance the signal while reducing common-mode noise.
(ii) Application of a moving average (over 25 subsequent points) subtraction to eliminate low-frequency oscillations, ensuring that high frequencies (>50 Hz) remain unaffected.
(iii) Removal of the initial 50 ms of data (since the OPM requires this time to regain sensitivity after saturation caused by the magnetic-field excitation pulse) leaves us with useful data points corresponding to $t_{\mathrm{acq}} = 2.048$ s (the sampling rate is 1 kHz).
(iv) Subtraction of analytical functions and sinusoidal components at 50 Hz and overtones from the original data in the time domain. Specifically, we employed the following fit model: $model = \{"exponential\ decays"\} + \{"power-line\ noise\ sinusoidals"\} +$

$\{"decaying\ sinusoidals"\} + \{"3rd - degree\ polynomial"\}$, resulting in a fit with a coefficient of determination $R^2 = 0.991$.

(v) Application of a 3rd-order bandpass filter to remove low-frequency noise, with low and high cutoff frequencies set at 20 Hz and 1000 Hz, respectively.

(vi) Additional baseline removal in the frequency domain by interpolating the baseline outside the regions of interest and subtracting it from the Fourier data. The complex data were phase-corrected to compensate for the initial dropped points and experimental imperfections.

(vii) Conversion of the spectra into magnetic-field units following the OPM calibration conversion factor of $0.9\ \mathrm{V/nT}$.

These steps applied to the entire dataset of 36000 spectra ensure the accuracy and quality of the analysis. In steps (ii) and (v), the applied filters had a low-frequency cutoff significantly lower than the lowest-frequency NMR peak. The baseline-reduction steps in frequency domain (step vi) included frequency ranges far from Lorentzian peaks.

It is important to note that the standard phase correction typically applied to high-field NMR spectra (0th and 1st order) proved insufficient for achieving universally positive absorptive lines in the ZULF NMR spectra. To address this, we implemented an interpolation of a frequency-dependent phase function $\varphi(\nu)$ enabling an absorptive phase across all relevant NMR peaks. The reason for this phase accumulation is currently under investigation. This modeling approach ensured the absence of baseline distortions introduced by high-order phase corrections.[3]

To obtain enough statistical points for extracting *J*-coupling frequencies, we performed identical shuttling experiments with prepolarization at 2 T for $N_0 = 36000$ scans. Different partitions were constructed from the original dataset, ranging from a single scan (the average of all 36000 scans) to groups of 10 averaged scans each (3600 partitions). For each partition, the whole processing procedure described above was conducted, with the same values of phase correction and baseline interpolation of the average of all scans were used for all of the partitions. We then fit the real part of the sum of five complex Lorentzians (each with variable phase parameter), to our complex data. From this fitting, we extrapolate the *J*-coupling values as well as the standard error of the fit.

Once we have extracted five *J*-coupling frequencies from the fit for each partition (**Figure 2**), we constructed the cumulative distribution function (CDF)[5] by (i) sorting all the values $\{\nu\}$ in ascending order in the horizontal axis and (ii) constructing $1/n$ points in the vertical axis, where $n$ is the number of partitions (e.g. for groups of 3000 averaged scans, the partition number is $n = N_0/3000 = 12$, thus, the vertical axis ranges as $\{1/12, 2/12, \ldots, 11/12, 1\}$ ). These so-called sigmoid curves are fit with a CDF of a normal distribution with mean $\mu$ and standard deviation $\sigma$:

$$\frac{1}{2}\mathrm{Erfc}\left(\frac{\mu - \nu}{\sqrt{2}\sigma}\right), \tag{S1}$$

Where Erfc is the error function. The result can be found in **Figure S1**. Note that only after the partition with more than four points or more (4th row and onwards), the fit is adequate. One can note that the sigmoids broaden with an increasing number of partitions since the fit of each individual averaged scan becomes less accurate (higher-order partitions have lower signal-to-noise ratio).[6]

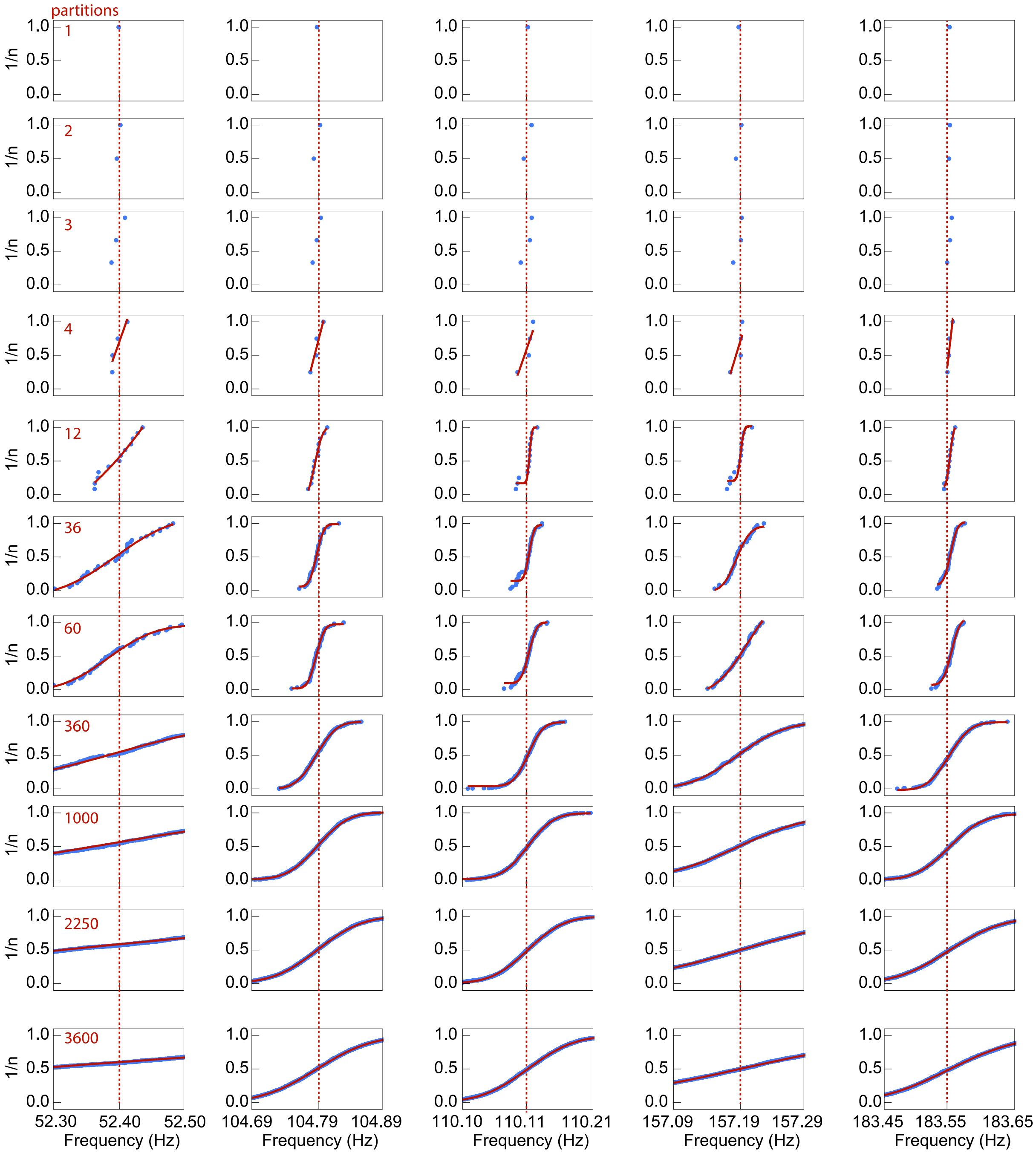


**Figure S1.** Sigmoid curves including fit with an increasing number of partitions.

From the sigmoid fits (**Figure S1**), we can extract the mean value of each frequency and the standard error of the mean (standard deviation divided by the square root of the number of measurements). Since there are several peaks corresponding to [$^{14}$N]- and [$^{15}$N]-ammonia, there are 6 different ways to extract the ratio of *J*-couplings. The results can be found in **Table S1**.

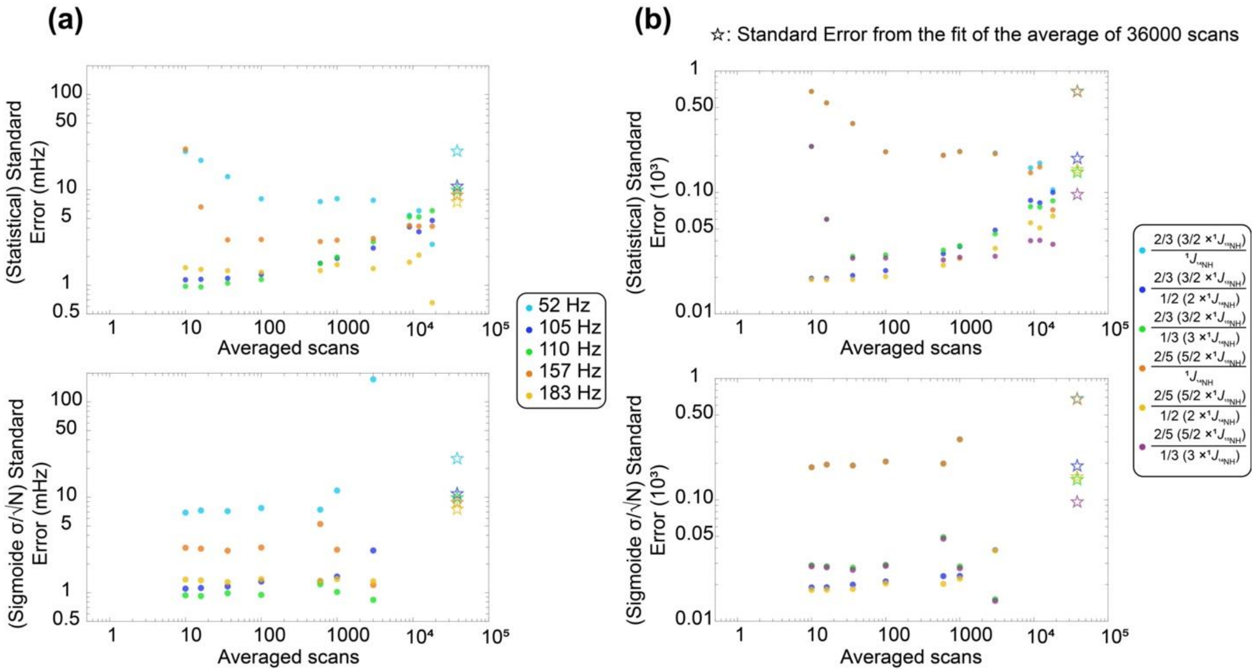


**Figure S2.** Standard error of the **(a)** *J*-coupling frequencies and **(b)** ratio of *J*-coupling frequencies extracted for different partitions of averaged scans. The top row shows values of the standard error determined from individual measurements (statistics) and the bottom shows the value of the standard error extracted from the fits of sigmoid curves.

**Figure S2** shows the standard error achieved as a function of number of partitions using the statistical (top) and CDF (bottom) method. The value obtained from the direct fit of all averaged scans is added with a ☆ symbol for comparison.

The standard error of the ratio of *J*-couplings in **Figure S2b** was obtained from the values in **Figure S2a** using error propagation formula:

$$\sigma_{\frac{J_{15_{\mathrm{NH}}}}{J_{14_{\mathrm{NH}}}}} \approx \left(\frac{J_{15_{\mathrm{NH}}}}{J_{14_{\mathrm{NH}}}}\right)\sqrt{\left(\frac{\sigma_{J_{15_{\mathrm{NH}}}}}{J_{15_{\mathrm{NH}}}}\right)^2 + \left(\frac{\sigma_{J_{14_{\mathrm{NH}}}}}{J_{14_{\mathrm{NH}}}}\right)^2}\ . \quad \text{(S2)}$$

The numerical values of **Figure S2** are also tabulated in **Table S2** for completeness. The *J*-couplings and their ratios are expressed in the order introduced in the **Table S1**.

**Table S1.** Frequencies of the zero-field peaks and their relationship to *J*-coupling values. Peaks of $^{14}\mathrm{N}H_4^+$ and $^{15}\mathrm{N}H_4^+$ are denoted as ①, ②, ③, and $\boxed{1}$, $\boxed{2}$, respectively.

| **Peak** | **Corresponding frequencies (Hz)** | **Relationship to *J*-coupling** | **Ways of extracting** $\left\lvert\frac{^1J_{15_{\mathrm{NH}}}}{^1J_{14_{\mathrm{NH}}}}\right\rvert$ **value** |
|---|---|---|---|
| ① | 52.4021(15) | $^1J_{14_{\mathrm{NH}}}$ | $(2/3)\times\boxed{1}/$ ① |
| ② | 104.7741(10) | $2\times{}^1J_{14_{\mathrm{NH}}}$ | $\frac{(2/3)\times\boxed{1}}{(1/2)\times ②}$ |
| ③ | 157.171(8) | $3\times{}^1J_{14_{\mathrm{NH}}}$ | $\frac{(2/3)\times\boxed{1}}{(1/3)\times ③}$ |
| $\boxed{1}$ | 110.114(9) | $(3/2)\times{}^1J_{15_{\mathrm{NH}}}$ | $\frac{(2/5)\times\boxed{2}}{①}$ |
| $\boxed{2}$ | 183.554(7) | $(5/2)\times{}^1J_{15_{\mathrm{NH}}}$ | $\frac{(2/5)\times\boxed{2}}{(1/2)\times ②}$ |
| | | | $\frac{(2/5)\times\boxed{2}}{(1/3)\times ③}$ |

**Table S2.** Standard Error of the ratio of *J*-coupling frequencies using two different methods. Note that the order of appearance of the $\left\lvert\frac{^1J_{15_{\mathrm{NH}}}}{^1J_{14_{\mathrm{NH}}}}\right\rvert$ values correspond to the order introduced in the **Table S1**.

| $N_0/n$ | $n$ | $\left\lvert\frac{^1J_{15_{\mathrm{NH}}}}{^1J_{14_{\mathrm{NH}}}}\right\rvert$ **(single-scan fit)** | $\left\lvert\frac{^1J_{15_{\mathrm{NH}}}}{^1J_{14_{\mathrm{NH}}}}\right\rvert$ **(statistical mean & error)** | $\left\lvert\frac{^1J_{15_{\mathrm{NH}}}}{^1J_{14_{\mathrm{NH}}}}\right\rvert$ **(mean & error from CDF fit)** |
|---|---|---|---|---|
| 1☆ | 36000 | 1.4009(7) | N/A | N/A |
| | | 1.40108(18) | | |
| | | 1.40103(14) | | |
| | | 1.4012(6) | | |
| | | 1.40134(15) | | |
| | | 1.40129(9) | | |
| 12 | 3000 | N/A | 1.40100(21) | 1.347(4) |
| | | | 1.40108(5) | 1.40116(4) |
| | | | 1.40102(5) | 1.401034(15) |
| | | | 1.40127(21) | 1.347(4) |
| | | | 1.40135(3) | 1.40139(4) |
| | | | 1.40129(3) | 1.401266(15) |
| 36 | 1000 | N/A | 1.40102(22) | 1.4011(3) |
| | | | 1.40108(4) | 1.401120(24) |
| | | | 1.40102(4) | 1.40109(3) |
| | | | 1.40129(22) | 1.40135(3) |
| | | | 1.40135(3) | 1.401365(22) |
| | | | 1.40129(3) | 1.40133(3) |
| 60 | 600 | N/A | 1.40111(20) | 1.4014(20) |
| | | | 1.40108(3) | 1.401124(24) |
| | | | 1.40102(3) | 1.40093(5) |
| | | | 1.40138(20) | 1.40166(20) |
| | | | 1.401355(25) | 1.401386(20) |
| | | | 1.40129(3) | 1.40119(5) |

| | | | | |
|---|---|---|---|---|
| 360 | 100 | N/A | 1.40159(22) | 1.40117(21) |
| | | | 1.401082(23) | 1.401109(21) |
| | | | 1.40102(3) | 1.40105(3) |
| | | | 1.40187(22) | 1.40142(21) |
| | | | 1.401360(20) | 1.401352(20) |
| | | | 1.40129(3) | 1.4013(3) |
| 1000 | 36 | N/A | 1.4028(4) | 1.40133(19) |
| | | | 1.40108(20) | 1.401073(20) |
| | | | 1.40101(3) | 1.40104(3) |
| | | | 1.4031(4) | 1.40159(19) |
| | | | 1.401362(19) | 1.401332(18) |
| | | | 1.40129(3) | 1.40130(3) |
| 2250 | 16 | N/A | 1.4035(5) | 1.40224(20) |
| | | | 1.401075(20) | 1.401071(19) |
| | | | 1.40109(6) | 1.40099(3) |
| | | | 1.4038(5) | 1.40249(20) |
| | | | 1.401361(19) | 1.401326(18) |
| | | | 1.40137(6) | 1.40124(3) |
| 3600 | 10 | N/A | 1.40368(7) | 1.40295(19) |
| | | | 1.401075(20) | 1.401089(19) |
| | | | 1.40193(24) | 1.4001(3) |
| | | | 1.40397(7) | 1.40321(19) |
| | | | 1.401368(19) | 1.401343(19) |
| | | | 1.40223(24) | 1.40125(3) |

## a. Systematic error in the analysis procedure

To test the robustness of the analysis procedure against baseline correction parameters, we studied the variation within the fitting results using different approaches to process the ZULF NMR spectra. **Table S3** is a collection of different reasonable processing steps typically used.

**Table S3**. Different options of the analysis procedure.

| Option | Dropped points (ms) | Moving average removal | Baseline correction in time domain | Bandpass filter | Zero filling | Phase correction in frequency domain | Baseline correction in frequency domain |
|---|---|---|---|---|---|---|---|
| **#1** | 0.05 | ✓ | ✓ | ✓ | ✓ | ✓ | ✗ |
| **#2** | 0.05 | ✓ | ✓ | ✓ | ✓ | ✓ | ✓ |
| **#3** | 0.05 | ✓ | ✓ | ✓ | ✓ | ✗ | ✓ |
| **#4** | 0.05 | ✓ | ✓ | ✓ | ✗ | ✓ | ✓ |
| **#5** | 0.15 | ✓ | ✓ | ✓ | ✓ | ✗ | ✗ |
| **#6** | 0.05 | ✓ | ✓ | ✗ | ✓ | ✓ | ✗ |
| **#7** | 0.05 | ✓ | ✓ | ✗ | ✓ | ✗ | ✗ |

| | | | | | | | |
|---|---|---|---|---|---|---|---|
| **#8** | 0.15 | ✓ | ✓ | ✗ | ✓ | ✓ | ✗ |
| **#9** | 0.15 | ✓ | ✓ | ✗ | ✓ | ✗ | ✗ |
| **#10** | 0.15 | ✓ | ✓ | ✗ | ✗ | ✓ | ✗ |
| **#11** | 0.05 | ✓ | ✓ | ✗ | ✗ | ✗ | ✗ |

It is worth noting that options #1-#5 applied the cleaning procedures on the average of all the partitions, whereas in options #6-11, the baseline of each individual scan was removed before averaging. The different 11 options under consideration yielded different fitted results, shown in **Table S4**.

**Table S4**. Fitted results using different options for the analysis procedure.

| **Statistical Analysis** | | | | | | | | | | | | |
|---|---|---|---|---|---|---|---|---|---|---|---|---|
| **Measured $\lvert J_{^{15}NH}/J_{^{14}NH}\rvert$ value** | **#1** | **#2** | **#3** | **#4** | **#5** | **#6** | **#7** | **#8** | **#9** | **#10** | **#11** | **Systematic Error (Statistical)** |
| $(4/3)\cdot(\boxed{1}/\textcircled{2})$ | 1.40107 ± 0.000023 | 1.40108 ± 0.000023 | 1.40108 ± 0.000021 | 1.40039 ± 0.000023 | 1.40124 ± 0.000028 | 1.40098 ± 0.000018 | 1.40113 ± 0.000018 | 1.40118 ± 0.000028 | 1.40124 ± 0.000028 | 1.40132 ± 0.000026 | 1.40025 ± 0.000026 | 0.0004 |
| $2\cdot(\boxed{1}/\textcircled{3})$ | 1.40106 ± 0.000032 | 1.40102 ± 0.000031 | 1.40086 ± 0.000025 | 1.40031 ± 0.000053 | 1.40135 ± 0.000033 | 1.40119 ± 0.000031 | 1.40116 ± 0.000025 | 1.40106 ± 0.000042 | 1.40115 ± 0.000035 | 1.40132 ± 0.00026 | 1.4007 ± 0.000026 | 0.0003 |
| $(4/5)\cdot(\boxed{2}/\textcircled{2})$ | 1.4014 ± 0.00002 | 1.40136 ± 0.00002 | 1.4018 ± 0.00002 | 1.40131 ± 0.000026 | 1.40125 ± 0.000029 | 1.40106 ± 0.000018 | 1.40137 ± 0.000019 | 1.40162 ± 0.000027 | 1.40134 ± 0.000028 | 1.40165 ± 0.00004 | 1.40082 ± 0.000032 | 0.0003 |
| $(6/5)\cdot(\boxed{2}/\textcircled{3})$ | 1.40139 ± 0.00003 | 1.40129 ± 0.000029 | 1.40157 ± 0.000024 | 1.40124 ± 0.000055 | 1.40136 ± 0.000034 | 1.40128 ± 0.000031 | 1.4014 ± 0.000025 | 1.4015 ± 0.000042 | 1.40125 ± 0.000035 | 1.40165 ± 0.00027 | 1.40127 ± 0.000032 | 0.00014 |
| **Cumulative Distribution Function fit** | | | | | | | | | | | | |
| **Measured $\lvert J_{^{15}NH}/J_{^{14}NH}\rvert$ value** | **#1** | **#2** | **#3** | **#4** | **#5** | **#6** | **#7** | **#8** | **#9** | **#10** | **#11** | **Systematic Error (CDF)** |
| $(4/3)\cdot(\boxed{1}/\textcircled{2})$ | 1.40108 ± 0.000022 | 1.4011 ± 0.000022 | 1.4011 ± 0.00002 | 1.40039 ± 0.000023 | 1.40125 ± 0.000027 | 1.40096 ± 0.000017 | 1.40111 ± 0.000017 | 1.40118 ± 0.000028 | 1.40124 ± 0.000028 | 1.40133 ± 0.000025 | 1.40021 ± 0.00002 | 0.0004 |
| $2\cdot(\boxed{1}/\textcircled{3})$ | 1.40107 ± 0.000031 | 1.40103 ± 0.00003 | 1.40087 ± 0.000025 | 1.40023 ± 0.000052 | 1.40135 ± 0.000033 | 1.4012 ± 0.000033 | 1.40116 ± 0.000026 | 1.40107 ± 0.000042 | 1.40117 ± 0.000033 | 1.40108 ± 0.000049 | 1.40067 ± 0.000025 | 0.0003 |
| $(4/5)\cdot(\boxed{2}/\textcircled{2})$ | 1.4014 ± 0.00002 | 1.40136 ± 0.00002 | 1.4018 ± 0.00002 | 1.40133 ± 0.000026 | 1.40125 ± 0.000027 | 1.40104 ± 0.000017 | 1.40135 ± 0.000018 | 1.40162 ± 0.000027 | 1.40133 ± 0.000028 | 1.40164 ± 0.00004 | 1.40077 ± 0.000028 | 0.0003 |
| $(6/5)\cdot(\boxed{2}/\textcircled{3})$ | 1.40139 ± 0.00003 | 1.4013 ± 0.000029 | 1.40157 ± 0.000024 | 1.40117 ± 0.000054 | 1.40135 ± 0.000033 | 1.40127 ± 0.000033 | 1.4014 ± 0.000026 | 1.4015 ± 0.000041 | 1.40126 ± 0.000033 | 1.40138 ± 0.000059 | 1.40122 ± 0.000032 | 0.00012 |

Since the results presented in the **Table S4** differ between the options well beyond the error bars of the individual values of the ratio, we construct in the last column a "systematic error" defined as the standard deviation of the results taking these 11 different options of data processing. These systematic errors are the error bars shown in **Figure 2c** of the main text.

As an example, **Figure S3** shows the results for options #2, #6, and #11, respectively, applied on 360 partitions of 100 averaged scans each. The black dashed lines indicate the values obtained by direct fit of all 360 scans, and the red dashed lines indicate the value that was obtained with the data collected in Berkeley using a homebuilt zero-field spectrometer.[7]

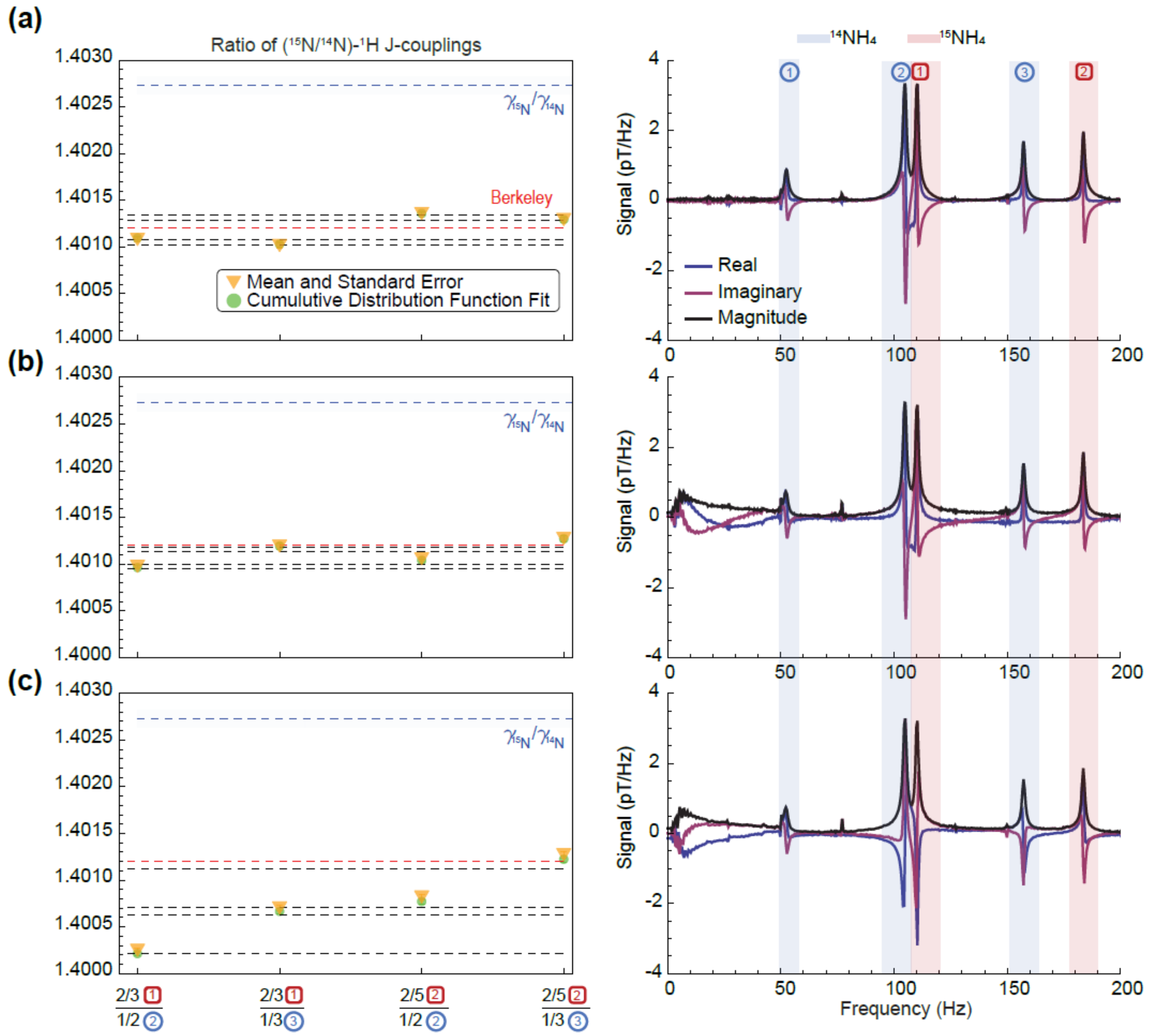


**Figure S3.** Results showing the estimated $\left|J_{^{15}\mathrm{NH}}/J_{^{14}\mathrm{NH}}\right|$ ratio using **(a)** option #2, **(b)** option #8, and **(c)** option #11 of the postprocessing procedure.

## B. Pulse-length dependence

To investigate opportunities of resolving $^1$H-D *J*-coupling with zero-field NMR techniques, we simulated the zero-field spectra for deuterated ammonium isotopologues and analyzed their energy level structures using perturbation theory.

For all isotopologues $^{15}\mathrm{ND}_x\mathrm{H}^+_{4-x}$ (where $x = 1 - 3$) one can distinguish regions of low- and high-frequency peaks (**Figure S4a**). High-frequency peaks correspond to transitions within the strongly-coupled subsystem consisting of $^{15}$N and $^1$H spins when total deuterium spin remains unchanged. Low-frequency peaks correspond to the deuterium spin flips keeping the strongly-coupled $^{15}$N-$^1$H subsystem unperturbed. These groups of peaks are expected to respond differently to the magnetic pulse excitation and thus, by plotting their integrals as a function of magnetic-pulse length, one can extract information

about subtle spin-spin interactions which otherwise would have taken more sophisticated multinuclear high-field NMR analysis.

For example, for $^{15}NDH_3^+$, one can clearly see that different $^1$H-D *J*-couplings result in different calculated dependencies of low-frequency integrals on the excitation pulse length (**Figure S4b**). Even a subtle change of ~3 Hz in $J_{HD}$ dramatically modifies the pulse length dependence. Importantly, not only a magnitude but also a sign of the *J*-coupling can be understood by experimentally probing the pulse-length dependence (**Figure 5**).

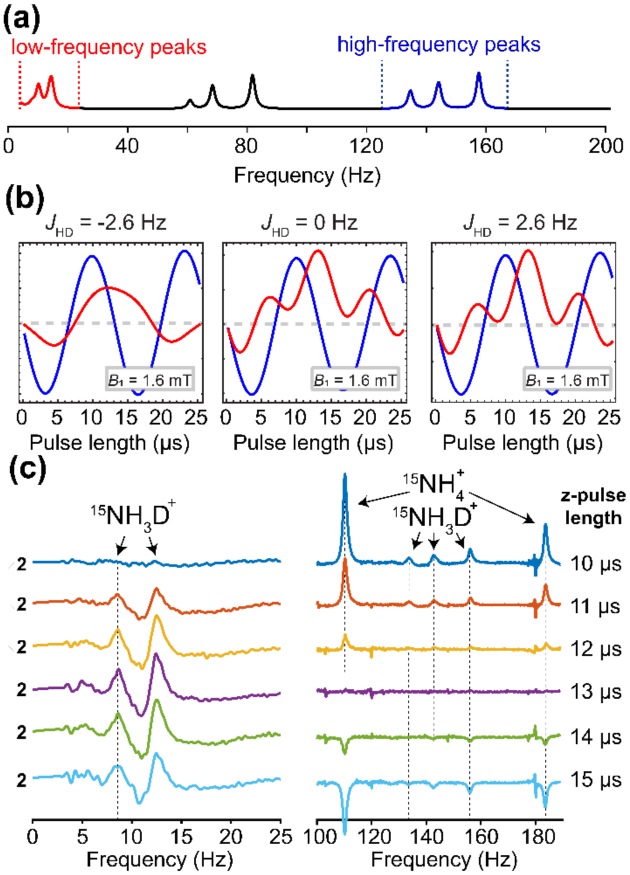


**Figure S4**. **(a)** Calculated zero-field J-spectrum of $^{15}NDH_3^+$ featuring low-frequency (0-15 Hz, red) and high-frequency (120-180 Hz, blue) regions of the spectrum. **(b)** Calculated integrals for the low-frequency (0-15 Hz, red) and high-frequency (120-180 Hz, blue) peaks in the spectrum of $^{15}NDH_3^+$ as a function of the magnetic pulse excitation length assuming $J_{HD}$ = -2.6 Hz (left), 0 Hz (middle), 2.6 Hz (right). **(c)** ZULF-NMR spectra of $^{15}ND_xH_{4-x}^+$ solution with deuterium fraction p = 24% recorded after the action of constant-amplitude magnetic-field pulses with various duration (10-15 μs, amplitude 1.6 mT) applied in the direction of the magnetometer-detection axis. Note that the low-frequency (0-15 Hz) and high-frequency (100-200 Hz) parts of the spectrum respond differently to the magnetic-pulse excitation.

## C. $T_1$ measurement of $^{15}$N ammonia

The $T_1$ measurement was performed in a Magritek Spinsolve 1.1 T on a solution of 6 M ammonium chloride with 50% $^{15}$N isotopic labeling dissolved in aqueous sulfuric acid (1.9 M). The proton $T_1$ measurement was carried out using a standard inversion-recovery experiment for the two different ammonium isotopologues (**Figure S5a-b**). The parameters for the experiment were: four scans, 6.4 s of acquisition, 2 min delay between scans, 10 s maximum inversion time, four dummy scans, and 27 steps. A refocused INEPT (insensitive nuclei enhanced by polarization transfer) pulse sequence was used to transfer polarization from the protons of $NH_4^+$ to $^{15}$N using an interpulse delay of 1/(4*J*) (|*J*|= 74 Hz) and a refocus delay of 1.135 ms. A 90° pulse was applied to convert the coherence generated in the refocused INEPT experiment back into $S_z$ spin order in $^{15}$N which was then allowed to relax for a variable time at 1 tesla before detection. The measured points represent integrals taken from $^{15}$N spectra that are the sum of 16 averages with an interscan delay of 20 s (**Figure S5c**). The estimated $T_1 = (49 \pm 3)$ s is the result of fitting with a mono-exponential decay function and error is estimated as the standard error of the fit.

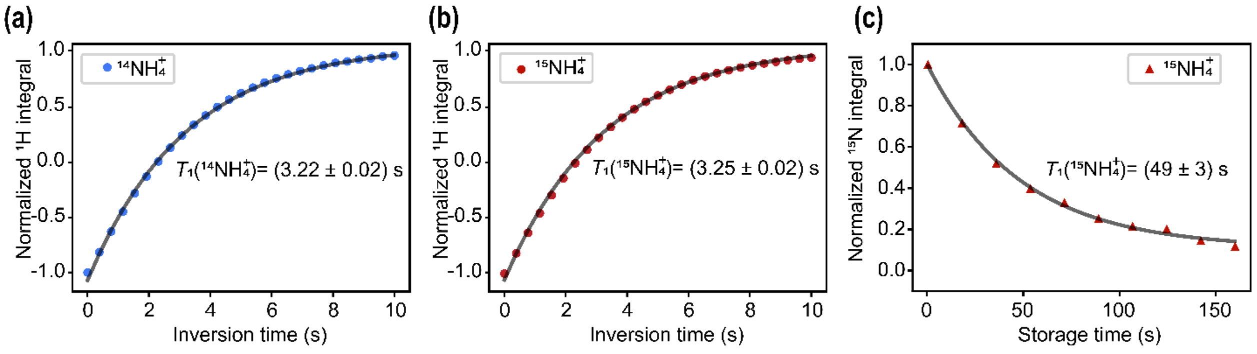


**Figure S5**. **(a)** Decay of an $^1$H signal as a function of inversion time measured for $^{14}$N-ammonium. **(b)** Inversion-recovery measurement for $^{15}$N-ammonium. **(c)** Decay of an $^{15}$N signal as a function of storage time measured using refocused INEPT.

## D. Analytical calculations

Amongst all possible nuclear spin interactions, only electron-mediated *J*-couplings are important for analyzing the zero-field NMR spectra of molecules in the liquid state. The nuclear spin Hamiltonian ($\hat{H}$) for different isotopologues of the ammonium cation ($^{15}ND_xH_{4-x}^+$, where $x$ can take values from 0 to 4) is therefore given by

$$\hat{H} = J_{NH}(\hat{\mathbf{S}} \cdot \hat{\mathbf{K}}_A) + J_{ND}(\hat{\mathbf{S}} \cdot \hat{\mathbf{K}}_B) + J_{HD}(\hat{\mathbf{K}}_A \cdot \hat{\mathbf{K}}_B)\,, \tag{S3}$$

where we follow the notation suggested by Butler et al.,[4] i.e., $\hat{\mathbf{S}}$ denotes the spin of the heteronucleus (in our case, $^{15}$N or $^{14}$N), $\hat{\mathbf{K}}_A$ denotes the total proton spin ($\hat{\mathbf{K}}_A = \sum_{i=1}^{4-x} \hat{\mathbf{I}}_i^{H}$), and $\hat{\mathbf{K}}_B$ denotes the total deuterium spin ($\hat{\mathbf{K}}_B = \sum_{i=1}^{x} \hat{\mathbf{I}}_i^{D}$). The values of the couplings were extracted from the experimentally measured spectra and are shown in Table **S5**. Notice that due to the electronic similarity of $^1$H and D, the coupling

between $^{15}$N and D was estimated as $J_{\mathrm{ND}} \approx J_{\mathrm{NH}}\left(\frac{\gamma_{\mathrm{D}}}{\gamma_{\mathrm{H}}}\right)$, ignoring isotope effects. Below we derive the zero-field NMR frequencies in *J*-spectra for various isotopologues of ammonium cation.

**Table S5.** Heteronuclear J-couplings in the $^{15}\mathrm{N}D_xH_{4-x}^{+}$ and $^{14}\mathrm{N}H_4^{+}$ spin systems measured in this work.

| ***J*-coupling** | **Associated peaks** | **Extracted coupling value (Hz)** |
|---|---|---|
| $^{1}J_{^{15}\mathrm{NH}}$ * | $(2/3) \times \boxed{1}$ | -73.410(6) |
| | $(2/5) \times \boxed{2}$ | -73.422(3) |
| $^{1}J_{^{14}\mathrm{NH}}$ * | ① | 52.4021(15) |
| | $(1/2) \times$ ② | 52.392(5) |
| | $(1/3) \times$ ③ | 52.390(3) |
| $^{1}J_{^{15}\mathrm{ND}}$ ** | | -11.3(1) |
| $^{2}J_{\mathrm{HD}}$ * | | -2.6(1) |

*data extracted from the fit of averaged 36000 zero-field NMR spectra;

**data extracted from the high-field (800 MHz) $^{15}$N NMR spectra.

## a. Zero-field NMR spectrum of $^{15}\mathrm{NH}_4^{+}$

For *x* = 0 Eq. (S3) simplifies to

$$\hat{H} = J_{\mathrm{NH}}\left(\hat{\mathbf{S}} \cdot \hat{\mathbf{K}}_{\mathrm{A}}\right). \tag{S4}$$

By introducing the total spin $\hat{\mathbf{F}}_{\mathrm{A}}$ of the $^{15}$N-$^{1}$H system as $\hat{\mathbf{F}}_{\mathrm{A}} = \hat{\mathbf{S}} + \hat{\mathbf{K}}_{\mathrm{A}}$, one can show that

$$\hat{\mathbf{S}} \cdot \hat{\mathbf{K}}_{\mathrm{A}} = \frac{1}{2}\left(\hat{\mathbf{F}}_{\mathrm{A}}^{2} - \hat{\mathbf{S}}^{\mathbf{2}} - \hat{\mathbf{K}}_{\mathrm{A}}^{2}\right). \tag{S5}$$

Thus, since the value of *S* equals to ½, eigenstates of the Hamiltonian (Eq. S4) denoted $|F_{\mathrm{A}}, K_{\mathrm{A}}\rangle$, can be expressed using quantum numbers $F_{\mathrm{A}}$ and $K_{\mathrm{A}}$ (corresponding to the operators $\hat{\mathbf{F}}_{\mathrm{A}}^{2}$ and $\hat{\mathbf{K}}_{\mathrm{A}}^{2}$) and their energies (*E*) can be determined using the equation

$$E = \frac{J_{\mathrm{NH}}}{2}\left(F_{\mathrm{A}}(F_{\mathrm{A}}+1) - S(S+1) - K_{\mathrm{A}}(K_{\mathrm{A}}+1)\right). \tag{S6}$$

Since $\hat{\mathbf{K}}_{\mathrm{A}}^{2}$ and $\hat{\mathbf{S}}^{\mathbf{2}}$ commute with the perturbation (magnetic DC pulse along *z*-direction), transitions between eigenstates are constrained by $\Delta K_{\mathrm{A}} = \Delta S = 0$, while $\Delta F_{\mathrm{A}} = 0, \pm 1$. Therefore, for $^{15}\mathrm{NH}_4^{+}$ we expect peaks at $\frac{5}{2}J_{\mathrm{NH}}$ and $\frac{3}{2}J_{\mathrm{NH}}$, corresponding to transitions between $\left|\frac{5}{2}, 2\right\rangle \rightarrow \left|\frac{3}{2}, 2\right\rangle$ and $\left|\frac{3}{2}, 1\right\rangle \rightarrow \left|\frac{1}{2}, 1\right\rangle$ as shown in **Figure 1b** and **Table S6**.

**Table S6.** Nuclear spin eigenstates, corresponding energies, allowed transitions and zero-field NMR spectral frequencies of $^{15}\mathrm{N}H_4^+$ ion.

| Eigenstates, $\lvert F_\mathrm{A}, K_\mathrm{A}\rangle$ | Energy | Transition | Frequency |
|---|---|---|---|
| $\left\lvert \frac{5}{2}, 2\right\rangle$ | $J_\mathrm{NH}$ | $\left\lvert \frac{5}{2}, 2\right\rangle \to \left\lvert \frac{3}{2}, 2\right\rangle$ | $\frac{5}{2}J_\mathrm{NH}$ |
| $\left\lvert \frac{3}{2}, 2\right\rangle$ | $-\frac{3}{2}J_\mathrm{NH}$ | $\left\lvert \frac{3}{2}, 1\right\rangle \to \left\lvert \frac{1}{2}, 1\right\rangle$ | $\frac{3}{2}J_\mathrm{NH}$ |
| $\left\lvert \frac{3}{2}, 1\right\rangle$ | $\frac{1}{2}J_\mathrm{NH}$ | | |
| $\left\lvert \frac{1}{2}, 1\right\rangle$ | $-J_\mathrm{NH}$ | | |
| $\left\lvert \frac{1}{2}, 0\right\rangle$ | 0 | | |

## b. Zero-field NMR spectrum of $^{15}\mathrm{NDH}_3^+$

Since $|J_\mathrm{ND}|, |J_\mathrm{HD}| < |J_\mathrm{NH}|$, we can apply perturbation theory and split the Hamiltonian (S4) into two parts:

$$\hat{H} = \hat{H}_0 + \hat{H}_1\,, \tag{S7}$$

where $\hat{H}_0 = J_\mathrm{NH}(\hat{\mathbf{S}} \cdot \hat{\mathbf{K}}_\mathrm{A})$ is the same as in Eq. (S4) and $\hat{H}_1 = J_\mathrm{ND}(\hat{\mathbf{S}} \cdot \hat{\mathbf{K}}_\mathrm{B}) + J_\mathrm{HD}(\hat{\mathbf{K}}_\mathrm{A} \cdot \hat{\mathbf{K}}_\mathrm{B})$. Hamiltonian $\hat{H}_1$ can be simplified further. Using geometric arguments[4], one may show that $\hat{\mathbf{S}}$ and $\hat{\mathbf{K}}_\mathrm{A}$ can be replaced by their projections on $\hat{\mathbf{F}}_\mathrm{A}$:

$$\hat{H}_1 = J_\mathrm{ND}(\hat{\mathbf{S}} \cdot \hat{\mathbf{K}}_\mathrm{B}) + J_\mathrm{HD}(\hat{\mathbf{K}}_\mathrm{A} \cdot \hat{\mathbf{K}}_\mathrm{B}) = J_\mathrm{ND}\frac{\langle \hat{\mathbf{S}} \cdot \hat{\mathbf{F}}_\mathrm{A}\rangle}{\langle \hat{\mathbf{F}}_\mathrm{A}^2\rangle}(\hat{\mathbf{F}}_\mathrm{A} \cdot \hat{\mathbf{K}}_\mathrm{B}) + J_\mathrm{HD}\frac{\langle \hat{\mathbf{K}}_\mathrm{A} \cdot \hat{\mathbf{F}}_\mathrm{A}\rangle}{\langle \hat{\mathbf{F}}_\mathrm{A}^2\rangle}(\hat{\mathbf{F}}_\mathrm{A} \cdot \hat{\mathbf{K}}_\mathrm{B}) =$$

$$= \frac{J_\mathrm{ND}^{\parallel} + J_\mathrm{HD}^{\parallel}}{2}\left(\hat{\mathbf{F}}^2 - \hat{\mathbf{F}}_\mathrm{A}^2 - \hat{\mathbf{K}}_\mathrm{B}^2\right), \tag{S8}$$

where $\hat{\mathbf{F}} = \hat{\mathbf{F}}_\mathrm{A} + \hat{\mathbf{K}}_\mathrm{B}$ is the total spin of the system. This notation suggests that there are no longer single couplings between protons and the heteronucleus to the deuterium atoms but rather a total coupling of the strongly coupled system $(\hat{\mathbf{S}}, \hat{\mathbf{K}}_\mathrm{A})$ to the weakly coupled deuterium $(\hat{\mathbf{K}}_\mathrm{B})$. Since $\hat{\mathbf{K}}_\mathrm{A} = \hat{\mathbf{F}}_\mathrm{A} - \hat{\mathbf{S}}$ and $\hat{\mathbf{S}} = \hat{\mathbf{F}}_\mathrm{A} - \hat{\mathbf{K}}_\mathrm{A}$, by analogy with (S5) one may find that

$$J_\mathrm{ND}^{\parallel} = J_\mathrm{ND}\frac{\langle \hat{\mathbf{S}} \cdot \hat{\mathbf{F}}_\mathrm{A}\rangle}{\langle \hat{\mathbf{F}}_\mathrm{A}^2\rangle} = J_\mathrm{ND}\frac{\left(F_\mathrm{A}(F_\mathrm{A}+1) + S(S+1) - K_\mathrm{A}(K_\mathrm{A}+1)\right)}{2F_\mathrm{A}(F_\mathrm{A}+1)}\,, \tag{S9}$$

and

$$J_\mathrm{HD}^{\parallel} = J_\mathrm{HD}\frac{\langle \hat{\mathbf{K}}_\mathrm{A} \cdot \hat{\mathbf{F}}_\mathrm{A}\rangle}{\langle \hat{\mathbf{F}}_\mathrm{A}^2\rangle} = J_\mathrm{HD}\frac{\left(F_\mathrm{A}(F_\mathrm{A}+1) + K_\mathrm{A}(K_\mathrm{A}+1) - S(S+1)\right)}{2F_\mathrm{A}(F_\mathrm{A}+1)}\,. \tag{S10}$$

Since $K_\mathrm{B}$ = 1, i.e., it is fixed, and we are left with $F$, $F_\mathrm{A}$, and $K_\mathrm{A}$ as the quantum numbers for defining the eigenstates of $\hat{H}$ which will be denoted as $\lvert F, F_\mathrm{A}, K_\mathrm{A}\rangle$. **Table S7** shows eigenstates, allowed transitions and corresponding spectral lines. Note that we distinguish between high-frequency and low-frequency transitions. High-frequency transitions are transitions in which the quantum number of the strongly

coupled system ($F_\mathrm{A}$) changes. If $\Delta F_\mathrm{A} = 0$ and only $F$ changes, we will refer to these transitions as low-frequency transitions. These transitions can therefore be seen as deuterium spin flip. The only allowed transitions are those that leave $K_\mathrm{A}$ unchanged.

**Table S7.** Nuclear spin eigenstates, corresponding energies, allowed transitions and zero-field NMR spectral frequencies of $^{15}\mathrm{N}DH_3^+$ ion ($K_B = 1$ and it is fixed).

| Eigenstate, $\lvert F, F_\mathrm{A}, K_\mathrm{A}\rangle$ | $E_0$ | $E_1$ |
|---|---|---|
| $\left\lvert 3, 2, \frac{3}{2}\right\rangle$ | $\frac{3}{4}J_\mathrm{NH}$ | $\frac{1}{2}(J_\mathrm{ND} + 3J_\mathrm{HD})$ |
| $\left\lvert 2, 2, \frac{3}{2}\right\rangle$ | $\frac{3}{4}J_\mathrm{NH}$ | $-\frac{1}{4}(J_\mathrm{ND} + 3J_\mathrm{HD})$ |
| $\left\lvert 1, 2, \frac{3}{2}\right\rangle$ | $\frac{3}{4}J_\mathrm{NH}$ | $-\frac{3}{4}(J_\mathrm{ND} + 3J_\mathrm{HD})$ |
| $\left\lvert 2, 1, \frac{3}{2}\right\rangle$ | $-\frac{5}{4}J_\mathrm{NH}$ | $\frac{1}{4}(-J_\mathrm{ND} + 5J_\mathrm{HD})$ |
| $\left\lvert 1, 1, \frac{3}{2}\right\rangle$ | $-\frac{5}{4}J_\mathrm{NH}$ | $-\frac{1}{4}(-J_\mathrm{ND} + 5J_\mathrm{HD})$ |
| $\left\lvert 0, 1, \frac{3}{2}\right\rangle$ | $-\frac{5}{4}J_\mathrm{NH}$ | $-\frac{1}{2}(-J_\mathrm{ND} + 5J_\mathrm{HD})$ |
| $\left\lvert 2, 1, \frac{1}{2}\right\rangle$ | $\frac{1}{4}J_\mathrm{NH}$ | $\frac{1}{2}(J_\mathrm{ND} + J_\mathrm{HD})$ |
| $\left\lvert 1, 1, \frac{1}{2}\right\rangle$ | $\frac{1}{4}J_\mathrm{NH}$ | $-\frac{1}{2}(J_\mathrm{ND} + J_\mathrm{HD})$ |
| $\left\lvert 0, 1, \frac{1}{2}\right\rangle$ | $\frac{1}{4}J_\mathrm{NH}$ | $-(J_\mathrm{ND} + J_\mathrm{HD})$ |
| $\left\lvert 1, 0, \frac{1}{2}\right\rangle$ | $-\frac{3}{4}J_\mathrm{NH}$ | 0 |

| Transition | Frequency (high) |
|---|---|
| $\left\lvert 3, 2, \frac{3}{2}\right\rangle \rightarrow \left\lvert 2, 1, \frac{3}{2}\right\rangle$ | $2J_\mathrm{NH} + \frac{3}{4}J_\mathrm{ND} + \frac{1}{4}J_\mathrm{HD}$ |
| $\left\lvert 2, 2, \frac{3}{2}\right\rangle \rightarrow \left\lvert 2, 1, \frac{3}{2}\right\rangle$ | $2J_\mathrm{NH} - 2J_\mathrm{HD}$ |
| $\left\lvert 2, 2, \frac{3}{2}\right\rangle \rightarrow \left\lvert 1, 1, \frac{3}{2}\right\rangle$ | $2J_\mathrm{NH} - \frac{1}{2}J_\mathrm{ND} + \frac{1}{2}J_\mathrm{HD}$ |
| $\left\lvert 1, 2, \frac{3}{2}\right\rangle \rightarrow \left\lvert 2, 1, \frac{3}{2}\right\rangle$ | $2J_\mathrm{NH} - \frac{1}{2}J_\mathrm{ND} - \frac{7}{2}J_\mathrm{HD}$ |
| $\left\lvert 1, 2, \frac{3}{2}\right\rangle \rightarrow \left\lvert 1, 1, \frac{3}{2}\right\rangle$ | $2J_\mathrm{NH} - J_\mathrm{ND} - J_\mathrm{HD}$ |
| $\left\lvert 1, 2, \frac{3}{2}\right\rangle \rightarrow \left\lvert 0, 1, \frac{3}{2}\right\rangle$ | $2J_\mathrm{NH} - \frac{5}{4}J_\mathrm{ND} + \frac{1}{4}J_\mathrm{HD}$ |

| | |
|---|---|
| $\left\|2,1,\frac{1}{2}\right\rangle \to \left\|1,0,\frac{1}{2}\right\rangle$ | $J_{\mathrm{NH}} + \frac{1}{2}J_{\mathrm{ND}} + \frac{1}{2}J_{\mathrm{HD}}$ |
| $\left\|1,1,\frac{1}{2}\right\rangle \to \left\|1,0,\frac{1}{2}\right\rangle$ | $J_{\mathrm{NH}} - \frac{1}{2}J_{\mathrm{ND}} - \frac{1}{2}J_{\mathrm{HD}}$ |
| $\left\|0,1,\frac{1}{2}\right\rangle \to \left\|1,0,\frac{1}{2}\right\rangle$ | $2J_{\mathrm{NH}} - J_{\mathrm{ND}} - J_{\mathrm{HD}}$ |

| Transition | Frequency (low) |
|---|---|
| $\left\|3,2,\frac{3}{2}\right\rangle \to \left\|2,2,\frac{3}{2}\right\rangle$ | $\frac{3}{4}J_{\mathrm{ND}} + \frac{9}{4}J_{\mathrm{HD}}$ |
| $\left\|2,2,\frac{3}{2}\right\rangle \to \left\|1,2,\frac{3}{2}\right\rangle$ | $\frac{1}{2}J_{\mathrm{ND}} + \frac{3}{2}J_{\mathrm{HD}}$ |
| $\left\|1,1,\frac{3}{2}\right\rangle \to \left\|0,1,\frac{3}{2}\right\rangle$ | $-\frac{1}{4}J_{\mathrm{ND}} + \frac{5}{4}J_{\mathrm{HD}}$ |
| $\left\|1,1,\frac{1}{2}\right\rangle \to \left\|0,1,\frac{1}{2}\right\rangle$ | $\frac{1}{2}J_{\mathrm{ND}} + \frac{1}{2}J_{\mathrm{HD}}$ |

### c. Zero-field NMR spectrum of $^{15}\mathrm{ND_2H_2^+}$

We use the same approach for calculating nuclear energy levels of the ion $^{15}\mathrm{ND_2H_2^+}$. However, here we have an additional degree of freedom since $K_{\mathrm{B}}$ can now take values 0, 1 and 2. States are denoted $|F, F_{\mathrm{A}}, K_{\mathrm{A}}\rangle$ and $K_{\mathrm{B}}$ is given explicitly (**Table S8**). One should note that perturbation theory will eventually break down for states with larger $K_{\mathrm{B}}$ (specifically, $K_{\mathrm{B}} = 2$ in this case) since the corresponding term in the Hamiltonian increases. However, states with $K_{\mathrm{B}} = 1$ are still approximated well by perturbation theory. The selection rules introduced above remain unchanged.

**Table S8.** Nuclear spin eigenstates, corresponding energies, allowed transitions and zero-field NMR spectral frequencies of $^{15}\mathrm{N}D_2H_2^+$ ion.

| $K_\mathrm{B}$ | Eigenstate, $\lvert F, F_\mathrm{A}, K_\mathrm{A}\rangle$ | $E_0$ | $E_1$ |
|---|---|---|---|
| 0 | $\left\lvert\frac{3}{2},\frac{3}{2},1\right\rangle$ | $\frac{1}{2}J_\mathrm{NH}$ | 0 |
| | $\left\lvert\frac{1}{2},\frac{1}{2},0\right\rangle$ | 0 | 0 |
| 1 | $\left\lvert\frac{5}{2},\frac{3}{2},1\right\rangle$ | $\frac{1}{2}J_\mathrm{NH}$ | $\frac{1}{2}\left(J_\mathrm{ND}+2J_\mathrm{HD}\right)$ |
| | $\left\lvert\frac{3}{2},\frac{3}{2},1\right\rangle$ | $\frac{1}{2}J_\mathrm{NH}$ | $-\frac{1}{3}(J_\mathrm{ND}+2J_\mathrm{HD})$ |
| | $\left\lvert\frac{1}{2},\frac{3}{2},1\right\rangle$ | $\frac{1}{2}J_\mathrm{NH}$ | $-\frac{5}{6}(J_\mathrm{ND}+2J_\mathrm{HD})$ |
| | $\left\lvert\frac{3}{2},\frac{1}{2},1\right\rangle$ | $-J_\mathrm{NH}$ | $-\frac{1}{6}(J_\mathrm{ND}-4J_\mathrm{HD})$ |
| | $\left\lvert\frac{1}{2},\frac{1}{2},1\right\rangle$ | $-J_\mathrm{NH}$ | $\frac{1}{3}\left(J_\mathrm{ND}-4J_\mathrm{HD}\right)$ |
| | $\left\lvert\frac{3}{2},\frac{1}{2},0\right\rangle$ | 0 | $\frac{1}{2}J_\mathrm{ND}$ |
| | $\left\lvert\frac{1}{2},\frac{1}{2},0\right\rangle$ | 0 | $-J_\mathrm{ND}$ |
| 2 | $\left\lvert\frac{7}{2},\frac{3}{2},1\right\rangle$ | $\frac{1}{2}J_\mathrm{NH}$ | $J_\mathrm{ND}+2J_\mathrm{HD}$ |
| | $\left\lvert\frac{5}{2},\frac{3}{2},1\right\rangle$ | $\frac{1}{2}J_\mathrm{NH}$ | $-\frac{1}{6}(J_\mathrm{ND}+2J_\mathrm{HD})$ |
| | $\left\lvert\frac{3}{2},\frac{3}{2},1\right\rangle$ | $\frac{1}{2}J_\mathrm{NH}$ | $-(J_\mathrm{ND}+2J_\mathrm{HD})$ |
| | $\left\lvert\frac{1}{2},\frac{3}{2},1\right\rangle$ | $\frac{1}{2}J_\mathrm{NH}$ | $-\frac{3}{2}(J_\mathrm{ND}+2J_\mathrm{HD})$ |
| | $\left\lvert\frac{5}{2},\frac{1}{2},1\right\rangle$ | $-J_\mathrm{NH}$ | $-\frac{1}{3}(J_\mathrm{ND}-4J_\mathrm{HD})$ |
| | $\left\lvert\frac{3}{2},\frac{1}{2},1\right\rangle$ | $-J_\mathrm{NH}$ | $\frac{1}{2}\left(J_\mathrm{ND}-4J_\mathrm{HD}\right)$ |
| | $\left\lvert\frac{1}{2},\frac{1}{2},1\right\rangle$ | $-J_\mathrm{NH}$ | $\left(J_\mathrm{ND}+2J_\mathrm{HD}\right)$ |
| | $\left\lvert\frac{5}{2},\frac{1}{2},0\right\rangle$ | 0 | $J_\mathrm{ND}$ |
| | $\left\lvert\frac{3}{2},\frac{1}{2},0\right\rangle$ | 0 | $-\frac{3}{2}J_\mathrm{ND}$ |

| $K_B$ | Transition | Frequency (high) |
|---|---|---|
| 1 | $\left\|\frac{5}{2},\frac{3}{2},1\right\rangle \rightarrow \left\|\frac{3}{2},\frac{1}{2},1\right\rangle$ | $\frac{3}{2}J_{NH}+\frac{2}{3}J_{ND}+\frac{1}{3}J_{HD}$ |
| | $\left\|\frac{3}{2},\frac{3}{2},1\right\rangle \rightarrow \left\|\frac{3}{2},\frac{1}{2},1\right\rangle$ | $\frac{3}{2}J_{NH}-\frac{1}{6}J_{ND}-\frac{4}{3}J_{HD}$ |
| | $\left\|\frac{3}{2},\frac{3}{2},1\right\rangle \rightarrow \left\|\frac{1}{2},\frac{1}{2},1\right\rangle$ | $\frac{3}{2}J_{NH}-\frac{2}{3}J_{ND}+\frac{2}{3}J_{HD}$ |
| | $\left\|\frac{1}{2},\frac{3}{2},1\right\rangle \rightarrow \left\|\frac{1}{2},\frac{1}{2},1\right\rangle$ | $\frac{3}{2}J_{NH}-\frac{7}{6}J_{ND}-\frac{1}{3}J_{HD}$ |
| 2 | $\left\|\frac{7}{2},\frac{3}{2},1\right\rangle \rightarrow \left\|\frac{5}{2},\frac{1}{2},1\right\rangle$ | $\frac{3}{2}J_{NH}+\frac{4}{3}J_{ND}+\frac{2}{3}J_{HD}$ |
| | $\left\|\frac{5}{2},\frac{3}{2},1\right\rangle \rightarrow \left\|\frac{5}{2},\frac{1}{2},1\right\rangle$ | $\frac{3}{2}J_{NH}+\frac{1}{6}J_{ND}-\frac{5}{3}J_{HD}$ |
| | $\left\|\frac{5}{2},\frac{3}{2},1\right\rangle \rightarrow \left\|\frac{3}{2},\frac{1}{2},1\right\rangle$ | $\frac{3}{2}J_{NH}-\frac{2}{3}J_{ND}+\frac{5}{3}J_{HD}$ |
| | $\left\|\frac{3}{2},\frac{3}{2},1\right\rangle \rightarrow \left\|\frac{5}{2},\frac{1}{2},1\right\rangle$ | $\frac{3}{2}J_{NH}-\frac{2}{3}J_{ND}-\frac{10}{3}J_{HD}$ |
| | $\left\|\frac{3}{2},\frac{3}{2},1\right\rangle \rightarrow \left\|\frac{3}{2},\frac{1}{2},1\right\rangle$ | $\frac{3}{2}J_{NH}-\frac{3}{2}J_{ND}$ |
| | $\left\|\frac{3}{2},\frac{3}{2},1\right\rangle \rightarrow \left\|\frac{1}{2},\frac{1}{2},1\right\rangle$ | $\frac{3}{2}J_{NH}-2J_{ND}+2J_{HD}$ |
| | $\left\|\frac{1}{2},\frac{3}{2},1\right\rangle \rightarrow \left\|\frac{3}{2},\frac{1}{2},1\right\rangle$ | $\frac{3}{2}J_{NH}-2J_{ND}-J_{HD}$ |
| | $\left\|\frac{1}{2},\frac{3}{2},1\right\rangle \rightarrow \left\|\frac{1}{2},\frac{1}{2},1\right\rangle$ | $\frac{3}{2}J_{NH}-\frac{5}{2}J_{ND}+J_{HD}$ |

| $K_B$ | Transition | Spectral frequency (low) |
|---|---|---|
| 1 | $\left\|\frac{5}{2},\frac{3}{2},1\right\rangle \rightarrow \left\|\frac{3}{2},\frac{3}{2},1\right\rangle$ | $\frac{5}{6}J_{ND}+\frac{5}{3}J_{HD}$ |
| | $\left\|\frac{3}{2},\frac{3}{2},1\right\rangle \rightarrow \left\|\frac{1}{2},\frac{3}{2},1\right\rangle$ | $\frac{1}{2}J_{ND}+J_{HD}$ |
| | $\left\|\frac{3}{2},\frac{1}{2},1\right\rangle \rightarrow \left\|\frac{1}{2},\frac{1}{2},1\right\rangle$ | $-\frac{1}{2}J_{ND}+2J_{HD}$ |
| | $\left\|\frac{3}{2},\frac{1}{2},0\right\rangle \rightarrow \left\|\frac{1}{2},\frac{1}{2},0\right\rangle$ | $\frac{3}{2}J_{ND}$ |
| 2 | $\left\|\frac{7}{2},\frac{3}{2},1\right\rangle \rightarrow \left\|\frac{5}{2},\frac{3}{2},1\right\rangle$ | $\frac{7}{6}J_{ND}+\frac{7}{3}J_{HD}$ |

| | |
|---|---|
| $\left\|\frac{5}{2},\frac{3}{2},1\right\rangle \rightarrow \left\|\frac{3}{2},\frac{3}{2},1\right\rangle$ | $\frac{5}{6}J_{\mathrm{ND}}+\frac{5}{3}J_{\mathrm{HD}}$ |
| $\left\|\frac{3}{2},\frac{3}{2},1\right\rangle \rightarrow \left\|\frac{1}{2},\frac{3}{2},1\right\rangle$ | $\frac{1}{2}J_{\mathrm{ND}}+J_{\mathrm{HD}}$ |
| $\left\|\frac{5}{2},\frac{1}{2},1\right\rangle \rightarrow \left\|\frac{3}{2},\frac{1}{2},1\right\rangle$ | $-\frac{5}{6}J_{\mathrm{ND}}+\frac{10}{3}J_{\mathrm{HD}}$ |
| $\left\|\frac{3}{2},\frac{1}{2},1\right\rangle \rightarrow \left\|\frac{1}{2},\frac{1}{2},1\right\rangle$ | $-\frac{1}{2}J_{\mathrm{ND}}+2J_{\mathrm{HD}}$ |
| $\left\|\frac{5}{2},\frac{1}{2},0\right\rangle \rightarrow \left\|\frac{3}{2},\frac{1}{2},0\right\rangle$ | $\frac{5}{2}J_{\mathrm{ND}}$ |

### d. Zero-field NMR spectrum of $^{15}\mathrm{ND_3H^+}$

Analogously, we derive energy levels and corresponding transitions for the $^{15}\mathrm{ND_3H^+}$ ion (**Table S9**). One may note that actual frequencies may deviate from the derived ones due to the fact that perturbation theory no longer holds for large $K_{\mathrm{B}}$ values.

**Table S9.** Nuclear spin eigenstates, corresponding energies, allowed transitions and zero-field NMR spectral frequencies of $^{15}\mathrm{N}D_3H^+$ ion.

| $K_{\mathrm{B}}$ | Eigenstate, $\|F,K_{\mathrm{B}},F_{\mathrm{A}}\rangle$ | $E_0$ | $E_1$ |
|---|---|---|---|
| 0 | $\|4,3,1\rangle$ | $\frac{1}{4}J_{\mathrm{NH}}$ | $\frac{3}{2}J_{\mathrm{ND}}+\frac{3}{2}J_{\mathrm{HD}}$ |
| | $\|3,3,1\rangle$ | $\frac{1}{4}J_{\mathrm{NH}}$ | $-\frac{1}{2}J_{\mathrm{ND}}-\frac{1}{2}J_{\mathrm{HD}}$ |
| | $\|2,3,1\rangle$ | $\frac{1}{4}J_{\mathrm{NH}}$ | $-2J_{\mathrm{ND}}-2J_{\mathrm{HD}}$ |
| | $\|3,2,1\rangle$ | $\frac{1}{4}J_{\mathrm{NH}}$ | $J_{\mathrm{ND}}+J_{\mathrm{HD}}$ |
| | $\|2,2,1\rangle$ | $\frac{1}{4}J_{\mathrm{NH}}$ | $-\frac{1}{2}J_{\mathrm{ND}}-\frac{1}{2}J_{\mathrm{HD}}$ |
| | $\|1,2,1\rangle$ | $\frac{1}{4}J_{\mathrm{NH}}$ | $-\frac{3}{2}J_{\mathrm{ND}}-\frac{3}{2}J_{\mathrm{HD}}$ |
| | $\|2,1,1\rangle$ | $\frac{1}{4}J_{\mathrm{NH}}$ | $\frac{1}{2}J_{\mathrm{ND}}+\frac{1}{2}J_{\mathrm{HD}}$ |
| | $\|1,1,1\rangle$ | $\frac{1}{4}J_{\mathrm{NH}}$ | $-\frac{1}{2}J_{\mathrm{ND}}-\frac{1}{2}J_{\mathrm{HD}}$ |
| | $\|0,1,1\rangle$ | $\frac{1}{4}J_{\mathrm{NH}}$ | $-J_{\mathrm{ND}}-J_{\mathrm{HD}}$ |
| | $\|1,0,1\rangle$ | $\frac{1}{4}J_{\mathrm{NH}}$ | 0 |
| | $\|3,3,0\rangle$ | $-\frac{3}{4}J_{\mathrm{NH}}$ | 0 |
| | $\|2,2,0\rangle$ | $-\frac{3}{4}J_{\mathrm{NH}}$ | 0 |
| | $\|1,1,0\rangle$ | $-\frac{3}{4}J_{\mathrm{NH}}$ | 0 |
| | $\|0,0,0\rangle$ | $-\frac{3}{4}J_{\mathrm{NH}}$ | 0 |

| Transition | Frequency (high) |
|---|---|
| $\|4,3,1\rangle \rightarrow \|3,3,0\rangle$ | $J_{NH} + \frac{3}{2}J_{ND} + \frac{3}{2}J_{HD}$ |
| $\|3,3,1\rangle \rightarrow \|3,3,0\rangle$ | $J_{NH} - \frac{1}{2}J_{ND} - \frac{1}{2}J_{HD}$ |
| $\|2,3,1\rangle \rightarrow \|3,3,0\rangle$ | $J_{NH} - 2J_{ND} - 2J_{HD}$ |
| $\|3,2,1\rangle \rightarrow \|2,2,0\rangle$ | $J_{NH} + J_{ND} + J_{HD}$ |
| $\|2,2,1\rangle \rightarrow \|2,2,0\rangle$ | $J_{NH} - \frac{1}{2}J_{ND} - \frac{1}{2}J_{HD}$ |
| $\|1,2,1\rangle \rightarrow \|2,2,0\rangle$ | $J_{NH} - \frac{3}{2}J_{ND} - \frac{3}{2}J_{HD}$ |
| $\|2,1,1\rangle \rightarrow \|1,1,0\rangle$ | $J_{NH} + \frac{1}{2}J_{ND} + \frac{1}{2}J_{HD}$ |
| $\|1,1,1\rangle \rightarrow \|1,1,0\rangle$ | $J_{NH} - \frac{1}{2}J_{ND} - \frac{1}{2}J_{HD}$ |
| $\|0,1,1\rangle \rightarrow \|1,1,0\rangle$ | $J_{NH} - J_{ND} - J_{HD}$ |

| Transition | Spectral frequency (high) |
|---|---|
| $\|4,3,1\rangle \rightarrow \|3,3,1\rangle$ | $2J_{ND} + 2J_{HD}$ |
| $\|3,3,1\rangle \rightarrow \|2,3,1\rangle$ | $\frac{3}{2}J_{ND} + \frac{3}{2}J_{HD}$ |
| $\|3,2,1\rangle \rightarrow \|2,2,1\rangle$ | $\frac{3}{2}J_{ND} + \frac{3}{2}J_{HD}$ |
| $\|3,2,1\rangle \rightarrow \|2,2,1\rangle$ | $J_{ND} + J_{HD}$ |
| $\|3,2,1\rangle \rightarrow \|2,2,1\rangle$ | $J_{ND} + J_{HD}$ |
| $\|3,2,1\rangle \rightarrow \|2,2,1\rangle$ | $\frac{1}{2}J_{ND} + \frac{1}{2}J_{HD}$ |

### e. Zero-field NMR spectrum of $^{15}ND_4^+$

For $^{15}ND_4^+$ ($x = 4$) the spectrum may be solved analytically without using perturbation theory. The Hamiltonian is given by

$$\hat{H} = J_{ND}(\hat{\mathbf{S}} \cdot \hat{\mathbf{K}}_B). \tag{S11}$$

The selection rules introduced above remain unchanged, but $\hat{\mathbf{K}}_{\mathrm{A}}$ is now replaced by $\hat{\mathbf{K}}_{\mathrm{B}}$. We therefore obtain the states and transitions shown in **Table S10**. States are denoted as $|F, K_{\mathrm{B}}\rangle$, here $F$ is the quantum number corresponding to $\hat{\mathbf{F}} = \hat{\mathbf{S}} + \hat{\mathbf{K}}_{\mathrm{B}}$.

**Table S10.** Nuclear spin eigenstates, corresponding energies, allowed transitions and zero-field NMR spectral frequencies of $^{15}\mathrm{N}D_4^+$ ion.

| Eigenstate, $\|F, K_{\mathrm{B}}\rangle$ | Energy | Transition | Frequency |
|---|---|---|---|
| $\left\|\frac{9}{2}, 4\right\rangle$ | $2J_{\mathrm{ND}}$ | $\left\|\frac{9}{2}, 4\right\rangle \rightarrow \left\|\frac{7}{2}, 4\right\rangle$ | $\frac{9}{2}J_{\mathrm{ND}}$ |
| $\left\|\frac{7}{2}, 4\right\rangle$ | $-\frac{5}{2}J_{\mathrm{ND}}$ | $\left\|\frac{7}{2}, 3\right\rangle \rightarrow \left\|\frac{5}{2}, 3\right\rangle$ | $\frac{7}{2}J_{\mathrm{ND}}$ |
| $\left\|\frac{7}{2}, 3\right\rangle$ | $\frac{3}{2}J_{\mathrm{ND}}$ | $\left\|\frac{5}{2}, 2\right\rangle \rightarrow \left\|\frac{3}{2}, 2\right\rangle$ | $\frac{5}{2}J_{\mathrm{ND}}$ |
| $\left\|\frac{5}{2}, 3\right\rangle$ | $-2J_{\mathrm{ND}}$ | $\left\|\frac{3}{2}, 1\right\rangle \rightarrow \left\|\frac{1}{2}, 1\right\rangle$ | $\frac{3}{2}J_{\mathrm{ND}}$ |
| $\left\|\frac{5}{2}, 2\right\rangle$ | $J_{\mathrm{ND}}$ | | |
| $\left\|\frac{3}{2}, 2\right\rangle$ | $-\frac{3}{2}J_{\mathrm{ND}}$ | | |
| $\left\|\frac{3}{2}, 1\right\rangle$ | $\frac{1}{2}J_{\mathrm{ND}}$ | | |
| $\left\|\frac{1}{2}, 1\right\rangle$ | $-J_{\mathrm{ND}}$ | | |
| $\left\|\frac{1}{2}, 0\right\rangle$ | $0$ | | |

## E. Analysis and simulations programs

### a. Python code to eliminate baseline from time-domain signals

```
# -*- coding: utf-8 -*-
"""
Created on Mon Jan 24 19:50:26 2022

@author: evandyke
"""

import tkinter as tk
from tkinter import filedialog
import numpy as np
import matplotlib.pyplot as plt
from scipy import fft
import scipy.interpolate as spline
from scipy.optimize import curve_fit
import lvm_read
from matplotlib.backends.backend_tkagg import (FigureCanvasTkAgg, NavigationToolbar2Tk)
import os as os
import random
import time as t

time0 = t.time()
zfSpectrum = []
fid = []
block = 1000  # number of scans to sum
count = block - 1  # counter for summing

"""
Select files with GUI
"""
# filepath = filedialog.askopenfilenames(filetypes=[('LVM files', '*.lvm')],
#                                        initialdir="path/to/data.lvm")

# filepath = filedialog.askopenfilenames(filetypes=[('LVM files', '*.lvm')],
# initialdir="path/to/data.lvm")

"""
Process all files in directory without GUI
"""
filepath = "path/to/data.lvm"  # designate path to data directory
scans = []
```

```python
for file in os.listdir(filepath):
    if file.endswith(".lvm"):  # finds all the lvm files and adds them to a list
        scans.append(file)

numberOfScans = len(scans)
for j in range(numberOfScans):

    datapath = filepath + '/' + random.choice(scans)
    Data = lvm_read.read(datapath)  # randomize processing

    # Data = lvm_read.read(filepath[j])
    # Data = lvm_read.read(random.choice(filepath))  # randomize processing
    data = (Data[0]['data'])
    # Fid = (data[:,0], data[:,1]) # Single channel detection
    fid = (data[:, 0], data[:, 4] - data[:, 2])  # Dual channel detection
    fid = np.array(fid)

    """
    Acquisition parameters
    """

    pointsToDrop = 49  # points to remove from front of data array
    fid = fid[:, (pointsToDrop):]  # shortens FID by pointsToDrop from beginning of array
    numPts = np.size(fid[0, :])  # reads number of data points after drop
    dt = fid[0, 1] - fid[0, 0]  # determines dwell time, i.e. inverse of sampling rate
    aqTime = fid[0, -1]  # takes total acquisition time as last number of time axis
    bandwidth = 1 / dt  # computes spectral bandwidth

    # print(f'numPts = {numPts}, aqTime = {aqTime}, \ndt = {dt}, bandwidth = {bandwidth}')

    """
    Signal parameters
    """

    timeAxis = np.array(fid[0, :])  # makes array of time points
    signal = fid[1, :]  # makes array of signal amplitudes

    """
    Fitting FID with Spline
    """

    splineFactor = 2  # spline factor is degree of polynomial to fit at each fitting section
    splineFitFID = spline.UnivariateSpline(timeAxis, signal,
                                           s=splineFactor)  # fits spline to time domain data to detrend low frequency noise
```

```
    splineFitFID = splineFitFID(timeAxis)  # generates spline curve to subtract from time domain data
    splineCorrectedFID = signal - splineFitFID  # subtracts spline from time domain data

    """
    Moving Average FID correction
    """
    frameSize = 25  # number of points in moving average
    movingAverage = []
    for i in range(np.size(signal)):
        chunk = splineCorrectedFID[i:i + frameSize]
        if len(chunk) < frameSize:  # shrinks the window size as moving averages approaches end of array
            frameSize = len(chunk)
        avg = np.sum(chunk) / frameSize  # computes moving average
        movingAverage.append(avg)
    polyMACorrectedFID = splineCorrectedFID - movingAverage  # subtracts moving average from time domain
data

    """
    Line broadening
    """

    # broadFactor = 0.2  # Hz
    # polyMACorrectedFID = polyMACorrectedFID * np.exp(-timeAxis * broadFactor)

    """
    Zero filling
    """
    fillFactor = 1
    zeroFillAmpl = np.pad(polyMACorrectedFID, (0, numPts * (fillFactor - 1)), 'constant', constant_values=0)
    zfTime = np.pad(timeAxis, (0, numPts * (fillFactor - 1)), 'linear_ramp',
                    end_values=(0, aqTime * fillFactor))
    zfSI = np.size(zeroFillAmpl)

    if j == 0:
        fids = np.zeros(np.size(zeroFillAmpl))  # make empty array on first loop

    fids = np.c_[fids, zeroFillAmpl]  # concatenate time domain signal column-wise into array

    fids = np.sum(fids, axis=1)  # sum columns of data into one column

    """
    Sum time domain data into partitions
    """

    if j == count:
```

```
        partitions = int(numberOfScans / block)

        fids = fids / block  # generate average of summed array

        if j == numberOfScans - 1:  # sets aside one partition for plotting
            partitionedFID = fids

        path = f'path/to/save/{partitions} partitions of {block} scans Spline and MA/'  # path to save partitions

        if not os.path.exists(path):  # creates folder in selected path if none exists
            os.mkdir(path)

        np.savetxt((path + f"scan {j + 1}.txt"), np.c_[zfTime, fids])

        fids = np.zeros(np.size(zeroFillAmpl))  # empties array so that new partition can be processed

        count = count + block  # increase the count by block size to trigger generation of next partition

"""
Fourier Transform
"""
freqCorrected = abs(
    fft.fftshift(fft.fft(partitionedFID, zfSI))) * dt / 0.9 * 1000  # fourier transform and convert to units of pT
zfx = np.arange(-bandwidth / 2, bandwidth / 2, bandwidth / zfSI)  # generate frequency axis for NMR spectrum

"""
Plot
"""
fig = plt.figure(dpi=600, figsize=(7, 7))  # create figure to view processing

ax0 = fig.add_subplot(221)
ax0.plot(timeAxis, signal, label='Original FID')
ax0.plot(np.transpose(timeAxis), splineFitFID, label='Spline Fit')
ax0.plot(np.transpose(timeAxis), splineCorrectedFID, label='Spline corrected FID')
ax0.plot(np.transpose(timeAxis), movingAverage, label='MA fit')
ax0.set_xlabel('Time (s)')
ax0.legend()

ax1 = fig.add_subplot(222)
ax1.plot(zfTime, partitionedFID, label='Corrected FID')
ax1.set_ylabel('Signal (V)')
ax1.set_xlabel('Time (s)')
ax1.legend()

ax2 = fig.add_subplot(212)
```

```
ax2.plot(zfx, freqCorrected, linewidth=1)
ax2.set_xlim([0, 200])
ax2.set_ylim([-0.01, 2.0])
ax2.set_ylabel('Signal (pT/Hz)')
ax2.set_xlabel('Frequency (Hz)')

fig.savefig(f'figure name.png', bbox_inches='tight')

plt.ion()
plt.show()

time1 = t.time()

print('running time = ', time1 - time0, 'seconds')
```

b. Python code to construct and export the different partitions

```
# -*- coding: utf-8 -*-
"""
Created on Mon Jan 24 19:50:26 2022

@author: evandyke
"""

import tkinter as tk
from tkinter import filedialog
import numpy as np
import matplotlib.pyplot as plt
from numpy import ndarray
from scipy import fft
import scipy.interpolate as spline
from scipy.optimize import curve_fit
import lvm_read
from matplotlib.backends.backend_tkagg import (FigureCanvasTkAgg, NavigationToolbar2Tk)
import os as os
```

```python
import random
import time as t

time0 = t.time()
zfSpectrum = []
fid = []
block = 1  # number of scans to sum
count = block - 1  # counter for summing

"""
Select files with GUI
"""
# filepath = filedialog.askopenfilenames(filetypes=[('LVM files', '*.lvm')],  initialdir="path/to/data")
#

"""
Process all files in directory without GUI
"""
filepath = "path/to/data"
scans = []

for file in os.listdir(filepath):
    if file.endswith(".lvm"):
        scans.append(file)

numberOfScans = len(scans)
for j in range(numberOfScans):

    datapath = filepath + '/' + random.choice(scans)
    Data = lvm_read.read(datapath)  # randomized processing

    # Data = lvm_read.read(filepath[j])  # uncomment for sequential processing with GUI
    # Data = lvm_read.read(random.choice(filepath))  # uncomment for randomized processing with GUI
    data = (Data[0]['data'])
    # Fid = (data[:,0], data[:,1]) # Single channel detection
    fid = (data[:, 0], data[:, 4] - data[:, 2])  # Dual channel detection
    fid = np.array(fid)

    """
    Acquisition parameters
    """

    pointsToDrop = 49
    fid = fid[:, pointsToDrop:]  # shortens FID
    numPts = np.size(fid[0, :])  # determines number of points in time domain
```

```
dt = fid[0, 1] - fid[0, 0]   # computes dwell time a.k.a. inverse sampling rate
aqTime = fid[0, -1]          # gets acquisition time by extracting last value of time axis
bandwidth = 1 / dt

# print(f'numPts = {numPts}, aqTime = {aqTime}, \ndt = {dt}, bandwidth = {bandwidth}')

"""
Signal parameters
"""

timeAxis: ndarray = np.array(fid[0, :])
signal = fid[1, :]

"""
Fitting FID with Spline
"""

splineFactor = 2
splineFitFID = spline.UnivariateSpline(timeAxis, signal, s=splineFactor)
splineFitFID = splineFitFID(timeAxis)
splineCorrectedFID = signal - splineFitFID

"""
Moving Average FID correction
"""
frameSize = 25  # number of points in moving average
movingAverage = []
for i in range(np.size(signal)):
    chunk = splineCorrectedFID[i:i + frameSize]
    if len(chunk) < frameSize:
        frameSize = len(chunk)
    chunk = np.sum(chunk) / frameSize
    movingAverage.append(chunk)
polyMACorrectedFID = splineCorrectedFID - movingAverage

"""
Line broadening
"""
# broadFactor = 0.2  # Hz
# polyMACorrectedFID = polyMACorrectedFID * np.exp(-timeAxis * broadFactor)

"""
Zero filling
"""
fillFactor = 1
```

```
    zeroFillAmpl = np.pad(polyMACorrectedFID, (0, numPts * (fillFactor - 1)), 'constant', constant_values=0)
    zfTime = np.pad(timeAxis, (0, numPts * (fillFactor - 1)), 'linear_ramp', end_values=(0, aqTime * fillFactor))
    zfSI = np.size(zeroFillAmpl)

    if j == 0:
        fids: ndarray = np.zeros(np.size(zeroFillAmpl))

    fids = np.c_[fids, zeroFillAmpl]

    if j > 0:
        fids = np.sum(fids, axis=1)

    """
    Sum time domain data into partitions
    """
    if j == 0:
        partitionedFID = np.zeros(np.size(zeroFillAmpl))
        fids = np.sum(fids, axis=1) / block

    if j == count:
        partitions = int(numberOfScans / block)

        fids = fids / block

        partitionedFID = np.c_[partitionedFID, fids]
        partitionedFID = np.sum(partitionedFID, axis=1)

        path = f'path/to/save/{partitions} partitions of {block} scans spline s2 and MA 25pts/'

        if not os.path.exists(path):
            os.mkdir(path)

        np.savetxt((path + f"scan {j + 1}.txt"), np.c_[zfTime, fids])

        fids = np.zeros(np.size(zeroFillAmpl))

        count = count + block

"""
Fourier Transform
"""
partitionedFID = partitionedFID / numberOfScans
bandwidth = round(bandwidth, 3)
freqCorrected = abs(fft.fftshift(fft.fft(partitionedFID, zfSI))) * dt / 0.9 * 1000  # convert to units of pT
zfx = np.arange(-bandwidth / 2, bandwidth / 2 + bandwidth / zfSI, bandwidth / zfSI)
```

```
zfx = zfx[:-1]

"""
Plot
"""

plt.clf()
fig = plt.figure(dpi=600, figsize=(7, 7))
ax6 = fig.add_subplot(222)
ax6.plot(zfTime, partitionedFID, label='Corrected FID')
ax6.set_ylabel('Signal (V)')
ax6.set_xlabel('Time (s)')
ax6.legend()

ax5 = fig.add_subplot(221)
ax5.plot(timeAxis, signal, label='Original FID')
ax5.plot(np.transpose(timeAxis), splineFitFID, label='Spline Fit')
# ax5.plot(np.transpose(timeAxis), fittedBaseline, label='Polynomial fit')
# ax5.plot(np.transpose(timeAxis), splineCorrectedFID, label='Spline corrected FID')
# ax5.plot(np.transpose(timeAxis), movingAverage, label='MA fit')
# ax5.plot(np.transpose(timeAxis), polyMACorrectedFID, label='MA corrected FID')

ax5.set_xlabel('Time (s)')
ax5.legend()

ax7 = fig.add_subplot(212)
ax7.plot(zfx, freqCorrected, linewidth=1)
ax7.set_xlim([0, 200])
ax7.set_ylim([-0.01, 2.0])
ax7.set_ylabel('Signal (pT/Hz)')
ax7.set_xlabel('Frequency (Hz)')
plt.savefig(f'{numberOfScans} scans spline s{splineFactor} MA window {frameSize}.png',
            bbox_inches='tight')

plt.ion()
plt.show()

time1 = t.time()

print('running time = ', time1 - time0, 'seconds')
```

### c. J-couplings ratio analysis of quadrupolar nuclei

The following documents are provided in separated files as supplementary information:

(i) Supplementary Software 1

(ii) Supplementary Data 1

### d. Simulation example of $^{15}NH_3$

The following documents are provided in separated files as supplementary information:

(i) Supplementary Software 2

(ii) Supplementary Data 2

## References


(1) Eills, J.; Picazo-Frutos, R.; Bondar, O.; Cavallari, E.; Carrera, C.; Barker, S. J.; Utz, M.; Aime, S.; Reineri, F.; Budker, D.; Blanchard, J. W. Metabolic Reactions Studied by Zero- and Low-Field Nuclear Magnetic Resonance. **2022**.

(2) Stern, Q.; Sheberstov, K. Simulation of NMR Spectra at Zero-and Ultra-Low Field from A to Z-a Tribute to Prof. Konstantin L'vovich Ivanov. https://doi.org/10.5194/mr-2022-18.

(3) Levitt, M. H. *Spin Dynamics: Basics of Nuclear Magnetic Resonance, 2nd Edition.*; 2009; Vol. 34A. https://doi.org/10.1002/cmr.a.20130.

(4) Butler, M. C.; Ledbetter, M. P.; Theis, T.; Blanchard, J. W.; Budker, D.; Pines, A. Multiplets at Zero Magnetic Field: The Geometry of Zero-Field NMR. *J. Chem. Phys.* **2013**, *138* (18), 184202. https://doi.org/10.1063/1.4803144.

(5) Feller, William. *An introduction to probability theory and its applications*. Vol. 2, John Wiley & Sons, **1991**.

(6) Poullet, J.-B.; Sima, D. M.; Van Huffel, S., MRS signal quantitation: A review of time- and frequency-domain methods. *J. Magn. Reson.* **2008,** *195* (2), 134-144.

(7) Blanchard, J. W. Zero and Ultra-Low-Field Nuclear Magnetic Resonance Spectroscopy Via Optical Magnetometry. University of California at Berkeley, 2014.
