# Supplementary material for: Zero-field J-spectroscopy of quadrupolar nuclei
Source: Nat Commun. 2024 May 27;15:4487. doi: 10.1038/s41467-024-48390-2 (PMC11637023; doi:10.1038/s41467-024-48390-2)
Supplement: Supplementary file 3 — Description of Additional Supplementary Files [file 41467_2024_48390_MOESM3_ESM.pdf]

Description of Additional Supplementary Files

File Name: Supplementary Data 1
Description: PDF document of a Mathematica notebook with the complete analysis all 36000 spectra, including Cumulative Distribution Fit analysis to obtain the precise *J*-coupling peak.

File Name: Supplementary Data 2
Description: PDF document of a Mathematica notebook producing the simulations shown in Figures 4 and 5.

File Name: Supplementary Data 3
Description: Text file with all 36000 scans averaged together in .lvm format. We subtract the 3rd column from the 5th column to obtain the gradiometric time signal.

File Name: Supplementary Software 1
Description: Mathematica notebook with the complete analysis all 36000 spectra, including Cumulative Distribution Fit analysis to obtain the precise *J*-coupling peak.

File Name: Supplementary Software 2
Description: Mathematica notebook producing the simulations shown in Figures 4 and 5.
