# Supplementary material for: Zero-field J-spectroscopy of quadrupolar nuclei
Source: Nat Commun. 2024 May 27;15:4487. doi: 10.1038/s41467-024-48390-2 (PMC11637023; doi:10.1038/s41467-024-48390-2)
Supplement: Supplementary file 4 — Supplementary Data 1 [file 41467_2024_48390_MOESM4_ESM.pdf]

PreInitializations

J-couplings ratio analysis of quadrupolar nuclei

For 36000 scans

2/15/24 17:19:23 In[]:=

```
datafolderMain = "T:\\Projects\\ZULF NMR\\Roman\\ZULF\\Papers\\Quadrupole
    project\\Phase IV Data\\Experiment 1 - Thermal spectra\\Partitions";
```

2/15/24 17:19:23 In[]:=

```
l = 1; p = 2;
```

Remove 0.05 s initial data ⇒ 5000*0.05=250 points. m stores is all the aux variables I need data[i,m]. Take every l element. s is less points for the plots. 5000*1.024=5120 points less for 3.072 s
p is channel

2/15/24 17:19:23 In[]:=

```
datafolder = FileNameJoin[{datafolderMain, "00001 partitions of 36000 scans"}];
Clear@data
```

2/15/24 17:19:23 In[]:=

```
For[i = 1, i ≤ 1, i++,
  data[1, 1, 1, i] = ToExpression@ReplaceStringR@
      Import[FileNameJoin[{datafolder, "Scan-" <> IntegerString[i, 10, 4] <> ".lvm"}],
        "Table", NumberPoint → ",", "FieldSeparators" → "\t"][[23 ;; -2 ;; l, {1, 3}]] // Quiet;
  data[2, 1, 1, i] = ToExpression@ReplaceStringR@
      Import[FileNameJoin[{datafolder, "Scan-" <> IntegerString[i, 10, 4] <> ".lvm"}],
        "Table", NumberPoint → ",", "FieldSeparators" → "\t"][[23 ;; -2 ;; l, {1, 5}]] // Quiet;
  data[3, 1, 1, i] = data[2, 1, 1, i] - data[1, 1, 1, i].DiagonalMatrix[{0, 1}];
 ];
```

2/15/24 17:19:24 In[]:=

```
rate = 1000;
tacq0 = 0.05; tacq = 2.048;
MA = 0.025;
ZF = tacq; LB = 0.;
```

2/15/24 17:19:24 In[]:=

```
k = 3; kmax = 3;
n = 1;
o = 1;
ν1 = 20; ν2 = 1000; order = 3;
```

2/15/24 17:19:24 In[]:=

```
s = 10;
ListPlot[{
   {#[[1]], Re[#[[2]]]} & /@ data[1, 1, n, o][[ ;; ;; s]],
   {#[[1]], Re[#[[2]]]} & /@ data[2, 1, n, o][[ ;; ;; s]],
   {#[[1]], Re[#[[2]]]} & /@ data[3, 1, n, o][[ ;; ;; s]]
  }, PlotRange → {{tacq0, All}, All}, Joined → False, Frame → {True, True, False, False},
  FrameLabel → {"Time [s]"}, PlotLegends → {"data", "dark", "difference"}] // Rasterize
```

2/15/24 17:19:24 Out[]=

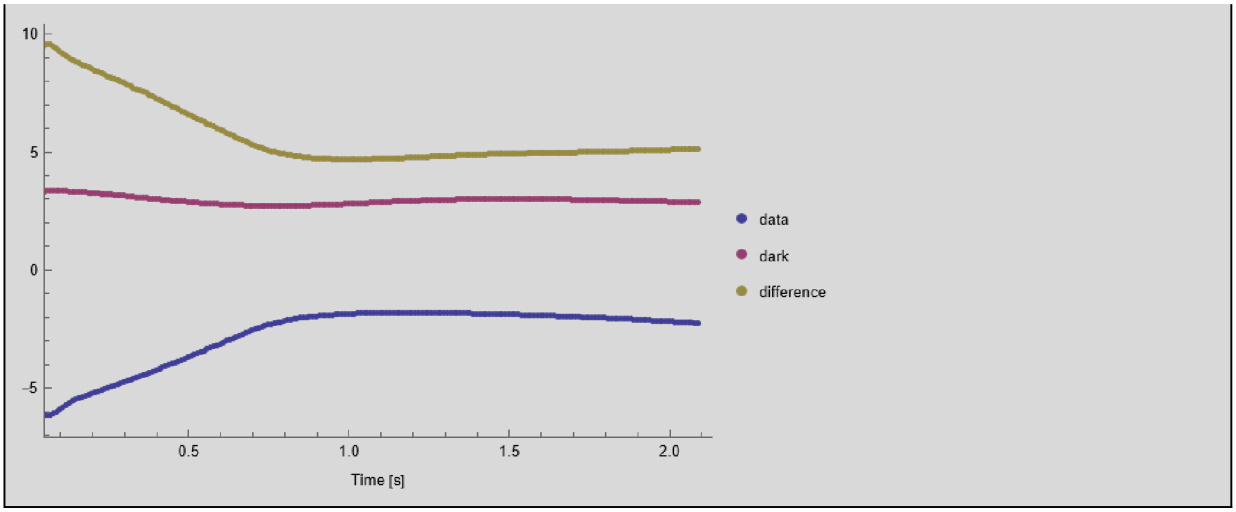

(*data[(...),2] is with Moving average removal in time domain*)

2/15/24 17:19:24 In[]:=

```
data[k, 2, n, o] =
  data[k, 1, n, o] - MovingAverageR[data[k, 1, n, o], MA, rate].DiagonalMatrix[{0, 1}];
s = 1;
ListPlot[{
  data[k, 1, n, o][[;; ;; s]],
  MovingAverageR[data[k, 1, n, o], MA, rate][[;; ;; s]],
  data[k, 2, n, o][[;; ;; s]]
 }, FrameLabel → {"Time [s]"}, PlotLegends → {"data", "Moving average", "difference"},
 PlotRange → {{0, 0.1}, All}, Joined → {False, True, False},
 PlotStyle → {Opacity@0.8, Opacity @@ {1, Red}, Opacity@1}]
```

2/15/24 17:19:24 Out[]=

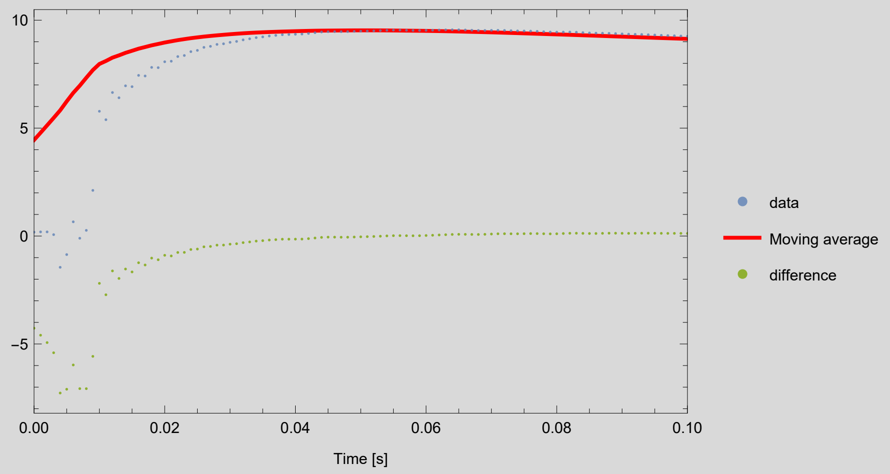

2/15/24 17:19:25 In[]:=

```
s = 1;
ListPlot[
 {{#[[1]], Abs@#[[2]]} & /@ fftR[data[k, 2, n, o][[tacq0 * rate ;; (tacq0 + tacq) * rate]][[;; ;; s]]]},
 PlotRange → {{0, 200}, 0.004 {0, 1}}]
```

2/15/24 17:19:25 Out[]=

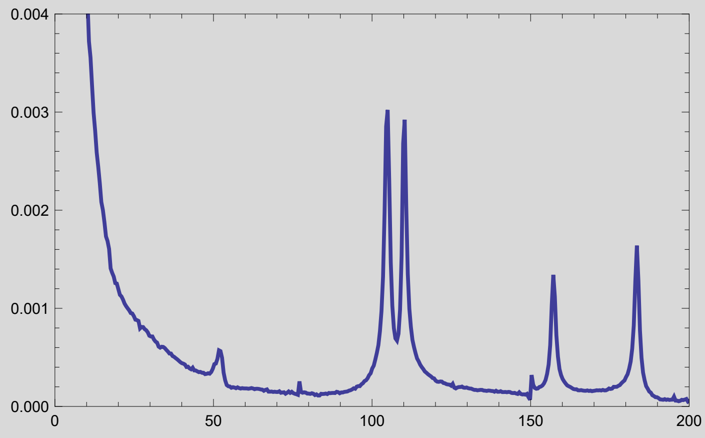

2/15/24 17:19:25 In[]:=

```
Clear[model, A, T, a, b, g, gmax, lines, τ]
gmax = 3;
lines = 50 {1, 3, 4};
model =
  Sum[A[i] E^(-t/τ[i]), {i, 0, 1}] + Re[Sum[a[i] E^(I (2 π lines[[i]] t + b[i])), {i, 1, 3}] + Sum[a[i] E^(-t/T[i]) E^(I (2 π ν[i] t + b[i])), {i, 4, 5}]] + Sum[g[i] t^i, {i, 0, gmax}];
parameters[k, 1, n, o] =
  {{A[0], -2.93}, {τ[0], 0.023}, A[1], τ[1], a[1], b[1], a[2], b[2], a[3], b[3], a[4],
    b[4], {ν[4], 0.7}, T[4], a[5], b[5], {ν[5], 0.7}, T[5]}~Join~(g /@ Range[0, gmax]);
fit[k, 1, n, o] = NonlinearModelFit[data[k, 2, n, o][[tacq0 * rate ;; (tacq0 + tacq) * rate]],
    model, parameters[k, 1, n, o], t, MaxIterations → Automatic] // Quiet;
fit150drop[k, 1, n, o] = NonlinearModelFit[data[k, 2, n, o][[0.15 * rate ;;]],
    model, parameters[k, 1, n, o], t, MaxIterations → Automatic] // Quiet;
{fit[k, 1, n, o]["RSquared"] // Quiet, fit150drop[k, 1, n, o]["RSquared"] // Quiet} // Column
s = 1;
{Show[{
     ListPlot[{data[k, 2, n, o][[tacq0 * rate ;; (tacq0 + tacq) * rate]][[ ;; ;; s]]},
      FrameLabel → {"Time [s]"}],
     Plot[fit[k, 1, n, o][t], {t, data[k, 2, n, o][[tacq0 * rate ;; (tacq0 + tacq) * rate]][[1, 1]],
       data[k, 2, n, o][[tacq0 * rate ;; -1 (*(tacq0+tacq)*rate*)]][[-1, 1]]},
      PlotStyle → {Red, Opacity@0.4}, FrameLabel → {"Time [s]"}]
    }] // Rasterize,
  Show[{
     ListPlot[{data[k, 2, n, o][[0.15 * rate ;;]][[ ;; ;; s]]}, FrameLabel → {"Time [s]"}],
     Plot[fit150drop[k, 1, n, o][t], {t, data[k, 2, n, o][[0.15 * rate ;;]][[1, 1]],
       data[k, 2, n, o][[tacq0 * rate ;; -1 (*(tacq0+tacq)*rate*)]][[-1, 1]]},
      PlotStyle → {Red, Opacity@0.4}, FrameLabel → {"Time [s]"}]
    }] // Rasterize} // Column
```

2/15/24 17:19:31 Out[]=

```
0.991054
0.993449
```

2/15/24 17:19:34 Out[]=

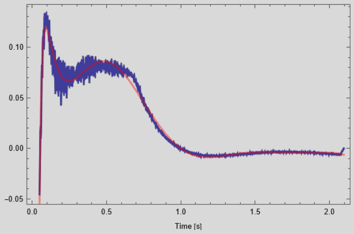

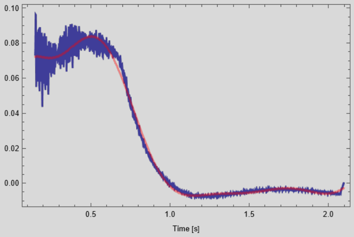

2/15/24 17:19:34 In[]:=

```
fit[k, 1, n, o]["ParameterTable"] // Quiet
```

2/15/24 17:19:40 Out[]=

	Estimate	Standard Error	t-Statistic	P-Value
A[0]	−5.24767	0.573698	−9.14709	1.38284×10^{-19}
τ[0]	0.0164416	0.000927594	17.725	1.81265×10^{-65}
A[1]	6.88751	18308.5	0.000376191	0.9997
τ[1]	1.20778	1284.86	0.000940008	0.99925
a[1]	−0.000174375	0.000139821	−1.24713	0.212494
b[1]	1.00848	0.80254	1.25661	0.20904
a[2]	0.0000430638	0.000139716	0.308224	0.757944
b[2]	1.00265	3.24472	0.309008	0.757347
a[3]	1.33462×10^{-6}	0.000139687	0.00955439	0.992378
b[3]	0.999576	104.644	0.00955215	0.99238
a[4]	−0.0280276	0.529753	−0.052907	0.957811
b[4]	0.62407	38.2335	0.0163226	0.986979
v[4]	0.970958	2.47952	0.39159	0.695402
T[4]	1.5336	39.4039	0.0389201	0.968958
a[5]	−1.53678	124.852	−0.0123088	0.99018
b[5]	−0.99372	99.3491	−0.0100023	0.99202
v[5]	0.435406	9.60234	0.0453437	0.963838
T[5]	0.277099	9.00976	0.0307554	0.975468
g[0]	−5.70222	18425.2	−0.000309479	0.999753
g[1]	3.08458	9590.14	0.000321641	0.999743
g[2]	−0.326808	1944.32	−0.000168083	0.999866
g[3]	−0.0589861	152.816	−0.000385994	0.999692

(*data[(...),3] is with baseline removal in time domain*)

```
(*////If we remove the fitting ////*)

data[k, 3, n, o] = Table[{data[k, 2, n, o][[tacq0*rate ;; (tacq0 + tacq) *rate]][[i, 1]],
     data[k, 2, n, o][[tacq0*rate ;; (tacq0 + tacq) *rate]][[i, 2]] -
      fit[k, 1, n, o][data[k, 2, n, o][[tacq0*rate ;; (tacq0 + tacq) *rate]][[i, 1]]]},
    {i, 1, Length@data[k, 2, n, o][[tacq0*rate ;; (tacq0 + tacq) *rate]]}] // Quiet;

(*////////////////////////////////*)


(*(*////If we remove the interpolation ////*)

data[k,3,n,o]=Table[{data[k,2,n,o][[tacq0*rate;;(tacq0+tacq)*rate]][[i,1]],
     data[k,2,n,o][[tacq0*rate;;(tacq0+tacq)*rate]][[i,2]]-
      interdat[phOrder][data[k,2,n,o][[tacq0*rate;;(tacq0+tacq)*rate]][[i,1]]]},
    {i,1,Length@data[k,2,n,o][[tacq0*rate;;(tacq0+tacq)*rate]]}]//Quiet;

(*////////////////////////////////*)*)

ListPlot[{
  {#[[1]], #[[2]]} & /@ data[k, 3, n, o][[;; ;; s]]
 }, PlotRange → All]

s = 1; ListPlot[{
  {#[[1]], Abs[#[[2]]]} & /@ fftR[data[k, 3, n, o]][[;; ;; s]]
 }, PlotRange → {{0, 200}, 0.004 {-0.5, 1}}]
```

2/15/24 17:19:41 Out[]=

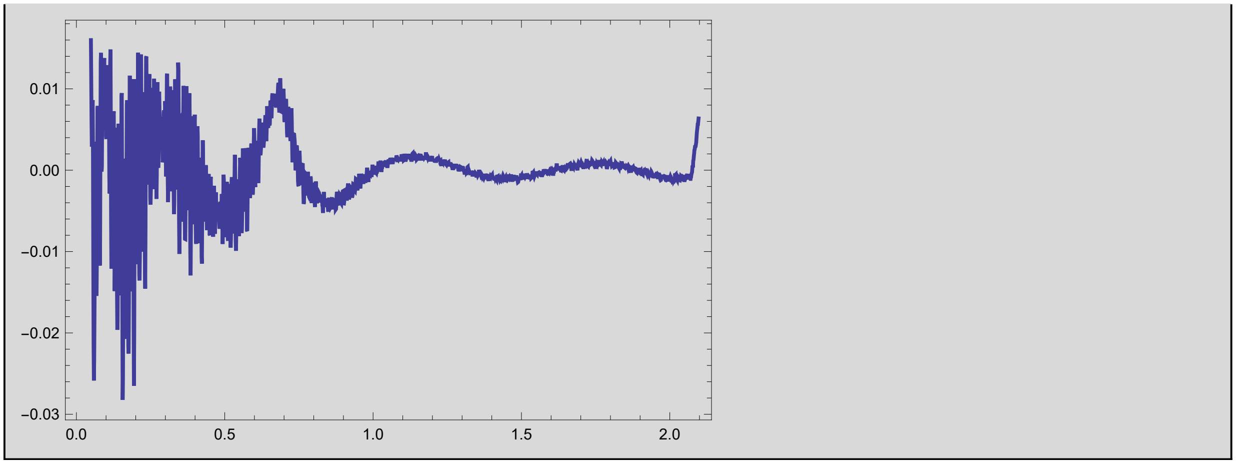

2/15/24 17:19:41 Out[]=

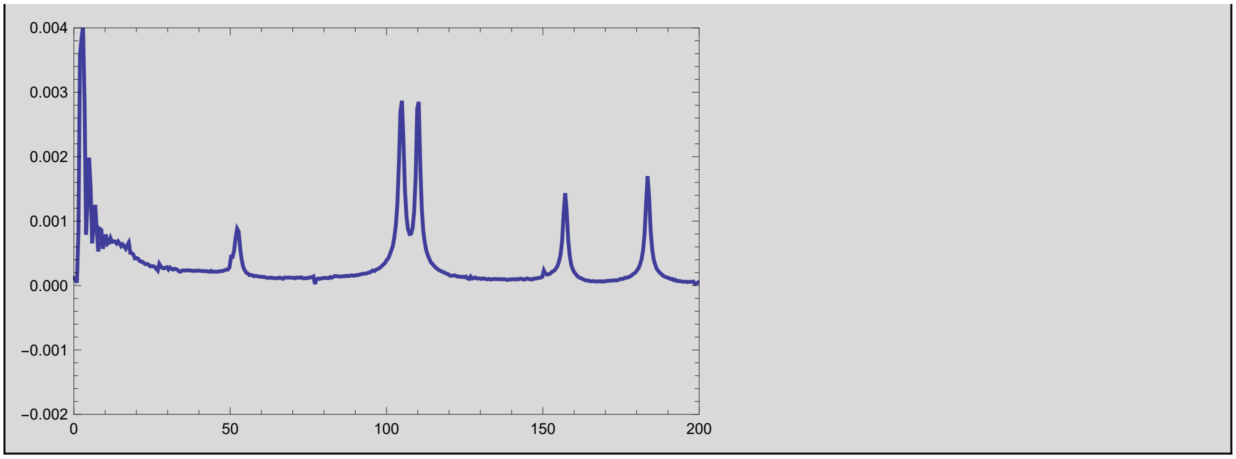

2/15/24 17:19:41 In[]:=

```
ListPlot[{
  {#⟦1⟧, #⟦2⟧} & /@
   (Table[{data[k, 2, n, o]⟦0.15*rate ;;⟧⟦i, 1⟧, data[k, 2, n, o]⟦0.15*rate ;;⟧⟦i, 2⟧ -
        fit150drop[k, 1, n, o][data[k, 2, n, o]⟦0.15*rate ;;⟧⟦i, 1⟧]},
      {i, 1, Length@data[k, 2, n, o]⟦0.15*rate ;;⟧}])⟦;; ;; s⟧
 }, PlotRange → All]

s = 1; ListPlot[{
  {#⟦1⟧, Abs[#⟦2⟧]} & /@
   fftR[(Table[{data[k, 2, n, o]⟦0.15*rate ;;⟧⟦i, 1⟧, data[k, 2, n, o]⟦0.15*rate ;;⟧⟦i, 2⟧ -
         fit150drop[k, 1, n, o][data[k, 2, n, o]⟦0.15*rate ;;⟧⟦i, 1⟧]},
       {i, 1, Length@data[k, 2, n, o]⟦0.15*rate ;;⟧}])]⟦;; ;; s⟧
 }, PlotRange → {{0, 200}, 0.004 {-0.5, 1}}]
```

2/15/24 17:19:42 Out[]=

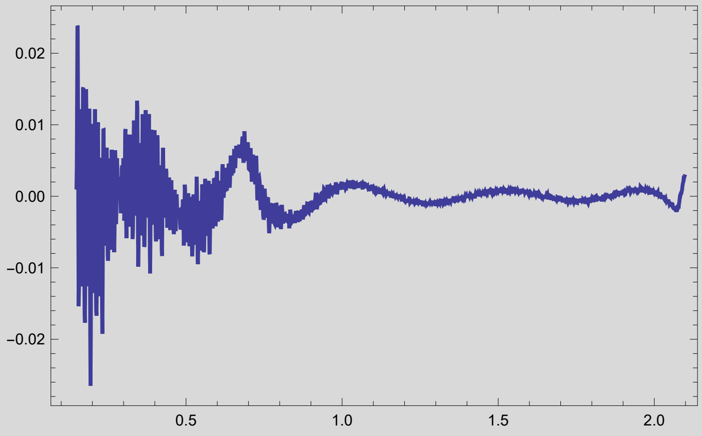

2/15/24 17:19:43 Out[]=

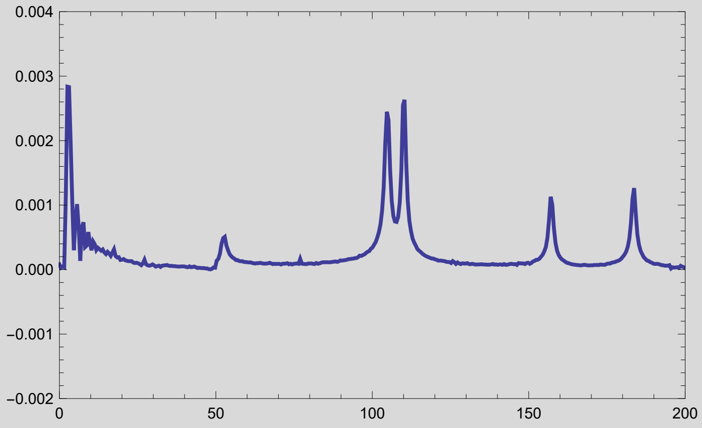

2/15/24 17:19:43 In[]:=

```
data[k, 3, n, o]
data[k, 4, n, o] = BandpassR[data[k, 3, n, o], ν1, ν2, order]
```

2/15/24 17:19:43 Out[]=

```
{{0.049, 0.0159882}, {0.05, 0.0115145}, {0.051, 0.00631066}, {0.052, 0.00288715}, {0.053, 0.0033677}, {0.054, 0.00779836},
 {0.055, 0.00861847}, {0.056, 0.0016613}, {0.057, -0.0107918}, {0.058, -0.0216213}, {0.059, -0.0258521},
 {0.06, -0.0233055}, {0.061, -0.0179589}, {0.062, -0.0110335}, {0.063, -0.00424963}, {0.064, 0.00201727},
 {0.065, 0.00347366}, {0.066, -0.00166503}, {0.067, -0.0103615}, {0.068, -0.0154485}, {0.069, -0.0111424},
 {0.07, -0.00117609}, {0.071, 0.00720627}, {0.072, 0.00789634}, {0.073, 0.00275919}, {0.074, 0.000103321},
 {0.075, 0.00268535}, {0.076, 0.00633588}, {0.077, 0.00362439}, {0.078, -0.00479023}, {0.079, -0.0117147}, … 1988 …,
 {2.068, -0.00101221}, {2.069, -0.00106913}, {2.07, -0.00106948}, {2.071, -0.000718213}, {2.072, -0.000724946},
 {2.073, -0.000506966}, {2.074, -0.000340576}, {2.075, -0.000239376}, {2.076, 0.00026132}, {2.077, 0.000574102},
 {2.078, 0.000336033}, {2.079, 0.0011523}, {2.08, 0.00128262}, {2.081, 0.00171669}, {2.082, 0.00202949},
 {2.083, 0.00249083}, {2.084, 0.00256734}, {2.085, 0.00289456}, {2.086, 0.0029074}, {2.087, 0.00293214},
 {2.088, 0.00315594}, {2.089, 0.00338821}, {2.09, 0.00396627}, {2.091, 0.00451334}, {2.092, 0.00490925},
 {2.093, 0.00519437}, {2.094, 0.00561883}, {2.095, 0.00563194}, {2.096, 0.00599883}, {2.097, 0.00635738}}
```

Full expression not available (original memory size: 197.7 kB)

2/15/24 17:19:52 Out[]=

```
{{0.049, 0.00747396}, {0.05, 0.0126864}, {0.051, 0.00144351}, {0.052, -0.00406733}, {0.053, -0.00243707},
 {0.054, -0.00171664}, {0.055, 0.00216621}, {0.056, -0.0030242}, {0.057, -0.0131109}, {0.058, -0.0205173},
 {0.059, -0.0221513}, {0.06, -0.0151617}, {0.061, -0.0059916}, {0.062, 0.0029246}, {0.063, 0.010455},
 {0.064, 0.0154432}, {0.065, 0.0165732}, {0.066, 0.011253}, {0.067, 0.00218631}, {0.068, -0.00390368},
 {0.069, -0.00170623}, {0.07, 0.0073737}, {0.071, 0.0149463}, {0.072, 0.0149774}, {0.073, 0.00811524},
 {0.074, 0.00160343}, {0.075, 0.0013995}, {0.076, 0.00384401}, {0.077, 0.00211898}, {0.078, -0.00554494}, … 1989 …,
 {2.068, -0.00014629}, {2.069, -0.000341343}, {2.07, -0.000204049}, {2.071, -5.45554×10^-6}, {2.072, 0.000188269},
 {2.073, 0.000207918}, {2.074, 0.000324047}, {2.075, 0.000401598}, {2.076, 0.000491717}, {2.077, 0.000802591},
 {2.078, 0.000523798}, {2.079, 0.000488574}, {2.08, 0.000905388}, {2.081, 0.000678657}, {2.082, 0.000683214},
 {2.083, 0.000785619}, {2.084, 0.000505179}, {2.085, 0.00035958}, {2.086, 0.000140904}, {2.087, -0.000187084},
 {2.088, -0.000340531}, {2.089, -0.000308669}, {2.09, -0.000190148}, {2.091, 0.0000863013}, {2.092, 0.000166576},
 {2.093, 0.0000259768}, {2.094, 9.24367×10^-6}, {2.095, -0.000166351}, {2.096, -0.000382954}, {2.097, -0.000289235}}
```

Full expression not available (original memory size: 197.7 kB)

2/15/24 17:19:52 In[]:=

```
s = 1; ListPlot[{
   {#[[1]], Abs[#[[2]]]} & /@ fftR[data[k, 3, n, o]][[;; ;; s]],
   {#[[1]], -0.003 + Abs[#[[2]]]} & /@ fftR[data[k, 4, n, o]][[;; ;; s]]
  }, PlotStyle → {Opacity@0.5, Automatic}, PlotRange → {{0, 200}, 0.004 {-1, 1}}] // Rasterize
```

2/15/24 17:19:53 Out[]=

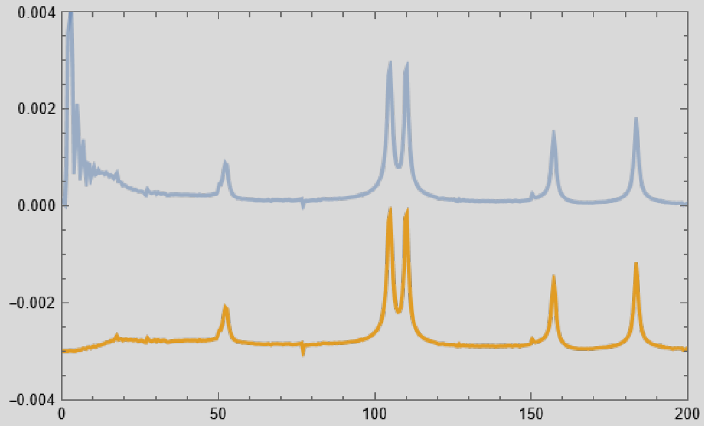

(*data[(...),5] is with zero filling and line broadening*)

2/15/24 17:19:53 In[]:=

```
teslaConv = 0.9 × 10^-3 (*Volts per pT*);
data[k, 5, n, o] = {#[[1]], 1/teslaConv e^(-LB #[[1]]) #[[2]]} & /@ Join[
    data[k, 4, n, o],
    Table[{data[k, 4, n, o][[-1, 1]] + i/rate, 0.}, {i, 1, ZF * rate}]
   ];
```

(*data[FT(...),1] is the complex frequency data of the time signal with zero filling and line broadening*)

2/15/24 17:19:53 In[]:=

```
Clear[phaseInter, interdat, phOrder];
phOrder = 2;
interdat[phOrder_] := Interpolation[
    {{52.3925, 0. °}, {Mean[{104.784, 110.115}], 165 °}, {157.171, 245 °}, {183.555, 315 °}}
    , InterpolationOrder → phOrder, Method → "Spline"] // Quiet;
Show[
 {Plot[interdat[phOrder][ν], {ν, 50, 200}, FrameLabel → {"Frequency (Hz)", "Phase (deg)"}] //
   Quiet, ListPlot[{{52.3925, 0. °}, {Mean[{104.784, 110.115}], 165 °},
    {157.171, 245 °}, {183.555, 315 °}}, Joined → False]}]
```

2/15/24 17:19:54 Out[]=

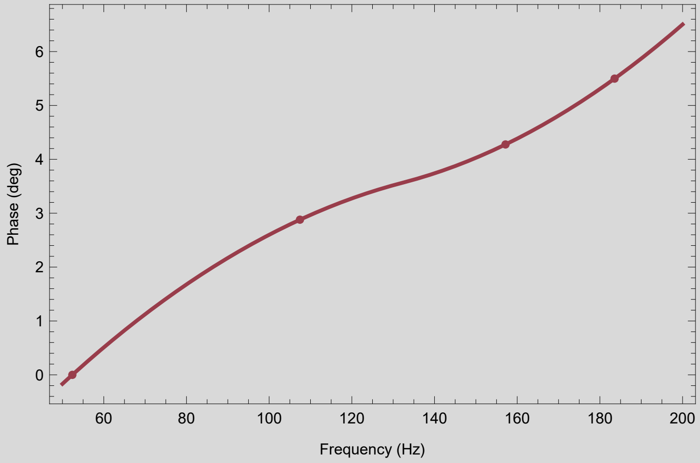

2/15/24 17:19:54 In[]:=

```
aux = {{52.3925, 48 °}, {Mean[{104.784, 110.115}], 203 °},
   {157.171, 88 ° + 360 °}, {183.555, 360 ° + 12 °}};
interdat150drop[phOrder_] :=
  Interpolation[{{52.3925, 48 °}, {Mean[{104.784, 110.115}], 203 °}, {157.171, 88 ° + 360 °},
     {183.555, 12 ° + 360 °}}, InterpolationOrder → phOrder, Method → "Spline"] // Quiet;
Show[{Plot[interdat150drop[phOrder][ν], {ν, 50, 200},
    FrameLabel → {"Frequency (Hz)", "Phase (deg)"}] // Quiet, ListPlot[aux, Joined → False]}]
```

2/15/24 17:19:54 Out[]=

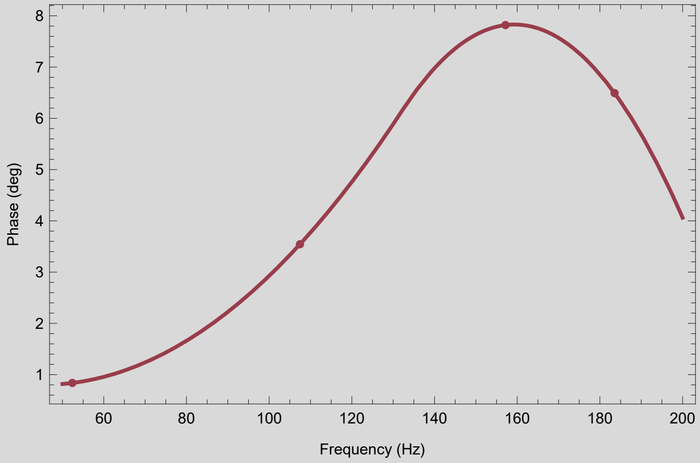

```
dataFT[k, 1, n, o] = {#⟦1⟧, e^(-i (interdat[phOrder][#⟦1⟧])) #⟦2⟧} & /@ fftR[data[k, 5, n, o]] // Quiet;
aux = {#⟦1⟧, e^(-i (interdat150drop[phOrder][#⟦1⟧])) #⟦2⟧} & /@ fftR[data[k, 5, n, o]⟦.1 * rate ;;⟧] // Quiet;
s = 1;
{ListPlot[{
    {#⟦1⟧, Re[#⟦2⟧]} & /@ dataFT[k, 1, n, o]⟦;; ;; s⟧,
    {#⟦1⟧, 4 + Im[#⟦2⟧]} & /@ dataFT[k, 1, n, o]⟦;; ;; s⟧
   }, PlotRange → {{0, 200}, 6 {-0.5, 1}},
   FrameLabel → {"Frequency (Hz)", "Signal (pT/Hz)"} & /@ dataFT[k, 1, n, o]⟦;; ;; s⟧],
  ListPlot[{
    {#⟦1⟧, Re[#⟦2⟧]} & /@ aux,
    {#⟦1⟧, 4 + Im[#⟦2⟧]} & /@ aux
   }, PlotRange → {{0, 200}, 6 {-0.5, 1}},
   FrameLabel → {"Frequency (Hz)", "Signal (pT/Hz)"} & /@ dataFT[k, 1, n, o]⟦;; ;; s⟧]} // Row
```

2/15/24 17:19:55 Out[]=

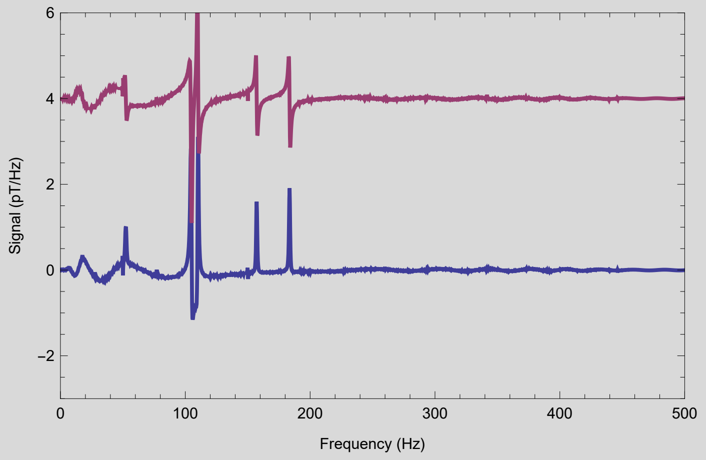

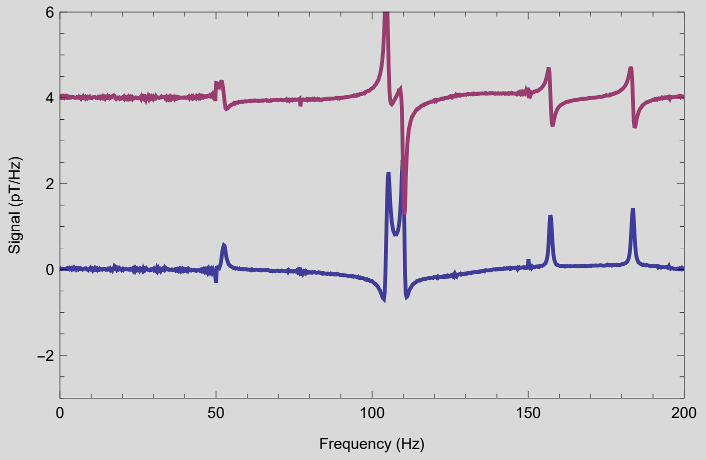

2/15/24 17:19:55 In[]:=

```
aux = {#[[1]], ⅇ^(-ⅈ (interdat[phOrder][#[[1]]])) #[[2]]} & /@ fftR[data[k, 5, n, o]] // Quiet;
s = 1; ListPlot[{
  {#[[1]], Re[#[[2]]]} & /@ aux[[ ;; ;; s]],
  {#[[1]], 4 + Im[#[[2]]]} & /@ aux[[ ;; ;; s]]
 }, PlotRange → {{0, 200}, 6 {-0.5, 1}},
 FrameLabel → {"Frequency (Hz)", "Signal (pT/Hz)"} & /@ dataFT[k, 1, n, o][[ ;; ;; s]]]
```

2/15/24 17:19:55 Out[]=

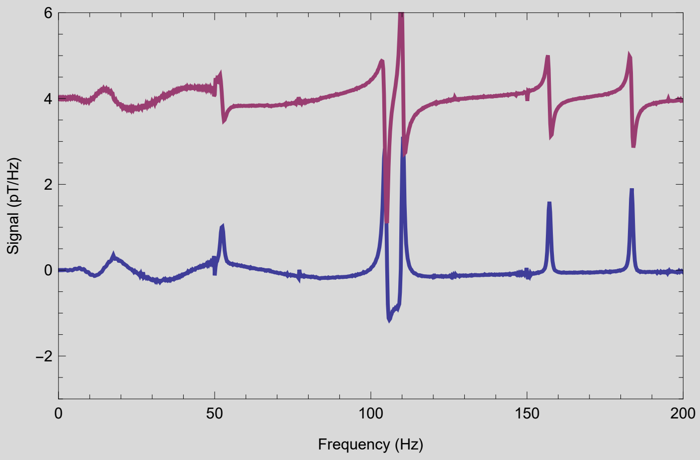


```
dataFTnoPhase[k, 1, n, o] = {#[[1]], ⅇ^(-ⅈ 0) #[[2]]} & /@ fftR[data[k, 5, n, o]] // Quiet;
```

2/15/24 17:19:55 In[]:=

```
Clear[a, ν, A, f, b, ϕ, g, gmax, model];
phOrder = 2;
dataFit[k, 1, n, o] = Join[
   Select[dataFT[k, 1, n, o], 0 < #[[1]] < 46 &],
   Select[dataFT[k, 1, n, o], 62 < #[[1]] < 87 &],
   (*Select[dataFT[k,1,n,o],78<#[[1]]<87&],*)
   Select[dataFT[k, 1, n, o], 125 < #[[1]] < 140 &],
   Select[dataFT[k, 1, n, o], 168 < #[[1]] < 173 &],
   Select[dataFT[k, 1, n, o], 200 < #[[1]] < 240 &]
  ];
dataFitnoPhase[k, 1, n, o] = Join[
   Select[dataFTnoPhase[k, 1, n, o], 0 < #[[1]] < 46 &],
   Select[dataFTnoPhase[k, 1, n, o], 62 < #[[1]] < 87 &],
   (*Select[dataFTnoPhase[k,1,n,o],78<#[[1]]<87&],*)
   Select[dataFTnoPhase[k, 1, n, o], 125 < #[[1]] < 140 &],
   Select[dataFTnoPhase[k, 1, n, o], 168 < #[[1]] < 173 &],
   Select[dataFTnoPhase[k, 1, n, o], 200 < #[[1]] < 240 &]
  ];
```

```
interdat[k, 2, 1, 1, 1][phOrder_] := Interpolation[
    {#[[1]], Re[#[[2]]]} & /@ dataFit[k, 1, 1, 1][[ ;; ;; 10]]
    , InterpolationOrder → phOrder, Method → "Spline"] // Quiet;
interdatnoPhase[k, 2, 1, 1, 1][phOrder_] := Interpolation[
    {#[[1]], Re[#[[2]]]} & /@ dataFitnoPhase[k, 1, 1, 1][[ ;; ;; 10]]
    , InterpolationOrder → phOrder, Method → "Spline"] // Quiet;




s = 1; Show[{
  ListPlot[{{#[[1]], Re[#[[2]]]} & /@ dataFit[k, 1, n, o][[ ;; ;; s]]},
   PlotRange → {{0, 250}, 0.8 {-0.5, 1}}],
  Plot[interdat[k, 2, 1, n, o][phOrder][ν],
    {ν, 0, 250}, FrameLabel → {"Frequency (Hz)", "Phase (deg)"}] // Quiet,
  ListPlot[{#[[1]], Re[#[[2]]]} & /@ dataFit[k, 1, n, o][[ ;; ;; 10]],
   PlotStyle → White, Joined → False]
 }]
```

2/15/24 17:19:56 Out[]=

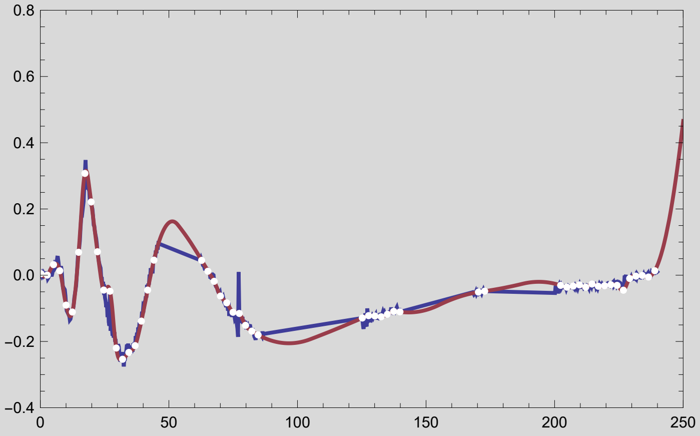

```
interdat[k, 2, 2, 1, 1][phOrder_] := Interpolation[
    {#[[1]], Im[#[[2]]]} & /@ dataFit[k, 1, 1, 1][[;; ;; 10]]
    , InterpolationOrder → phOrder, Method → "Spline"] // Quiet;
interdatnoPhase[k, 2, 2, 1, 1][phOrder_] := Interpolation[
    {#[[1]], Im[#[[2]]]} & /@ dataFitnoPhase[k, 1, 1, 1][[;; ;; 10]]
    , InterpolationOrder → phOrder, Method → "Spline"] // Quiet;


s = 1; Show[{
   ListPlot[{{#[[1]], Im[#[[2]]]} & /@ dataFit[k, 1, n, o][[;; ;; s]]},
    PlotRange → {{0, 250}, 0.6 {-0.5, 1}}],
   (*Plot[fit[k,2,2,n,o][ν],{ν,0,400},PlotStyle→Red],*)
   Plot[interdat[k, 2, 2, n, o][phOrder][ν],
     {ν, 0, 250}, FrameLabel → {"Frequency (Hz)", "Phase (deg)"}] // Quiet,
   ListPlot[
    {#[[1]], Im[#[[2]]]} & /@ dataFit[k, 1, n, o][[;; ;; 10]], PlotStyle → White, Joined → False]
  }] // Rasterize
```

2/15/24 17:19:57 Out[]=

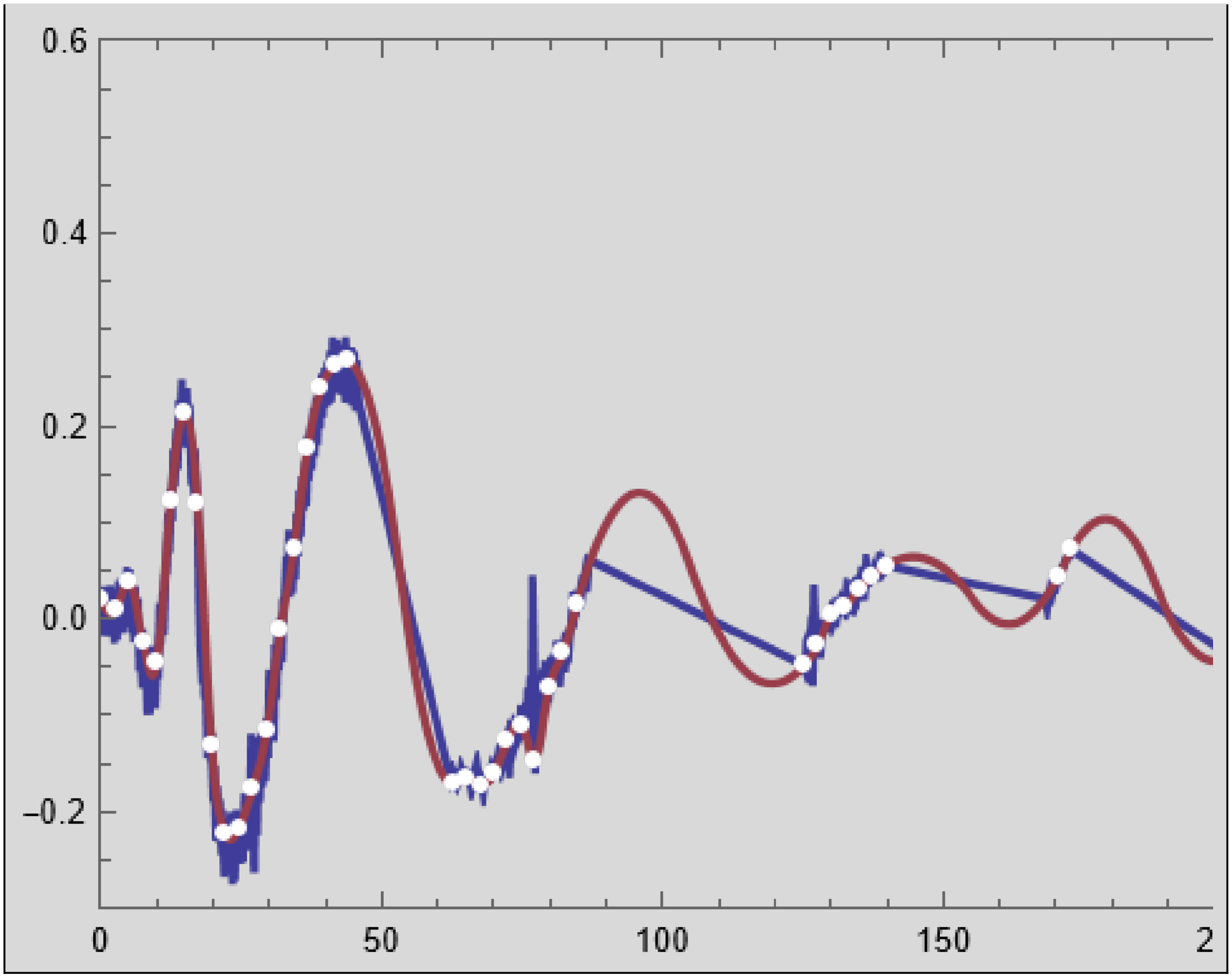

```
dataFT[k, 2, n, o] = Table[{dataFT[k, 1, n, o][[i, 1]],
      dataFT[k, 1, n, o][[i, 2]] - (interdat[k, 2, 1, n, o][phOrder][dataFT[k, 1, n, o][[i, 1]]] +
         ⅈ interdat[k, 2, 2, n, o][phOrder][dataFT[k, 1, n, o][[i, 1]]])},
     {i, 1, Length@dataFT[k, 1, n, o]}] // Quiet;

(*////////////////////////////////*)

β = 0 °;
s = 1;
range = {{0, 200}, 2 {-1, 2}};
plot[k, 1, n, o] = GraphicsRow[{
   ListPlot[{
     {#[[1]], Re[ⅇ^(-ⅈ β) #[[2]]]} & /@ dataFT[k, 1, n, o][[ ;; ;; s]],
     {#[[1]], Im[ⅇ^(-ⅈ β) #[[2]]]} & /@ dataFT[k, 1, n, o][[ ;; ;; s]],
     {#[[1]], Abs[ⅇ^(-ⅈ β) #[[2]]]} & /@ dataFT[k, 1, n, o][[ ;; ;; s]]
    }
    ,
    PlotRange → range, PlotLabel → "dataFT[k,1,n,o]",
    FrameLabel → {"Frequency (Hz)", "Signal (pT/Hz)"},
    Frame → {True, True, False, False}],
   ListPlot[{
     {#[[1]], Re[ⅇ^(-ⅈ β) #[[2]]]} & /@ dataFT[k, 2, n, o][[ ;; ;; s]],
     {#[[1]], Im[ⅇ^(-ⅈ β) #[[2]]]} & /@ dataFT[k, 2, n, o][[ ;; ;; s]],
     {#[[1]], Abs[ⅇ^(-ⅈ β) #[[2]]]} & /@ dataFT[k, 2, n, o][[ ;; ;; s]]
    }
    ,
    PlotRange → range, PlotLabel → "dataFT[k,2,n,o]",
    FrameLabel → {"Frequency (Hz)", "Signal (pT/Hz)"},
    Frame → {True, True, False, False}]
  }]
```

2/15/24 17:20:05 Out[]=

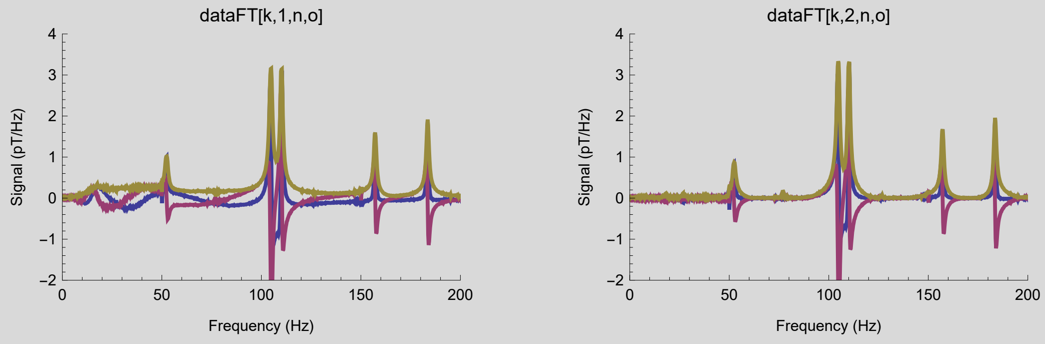

(*The groupings are 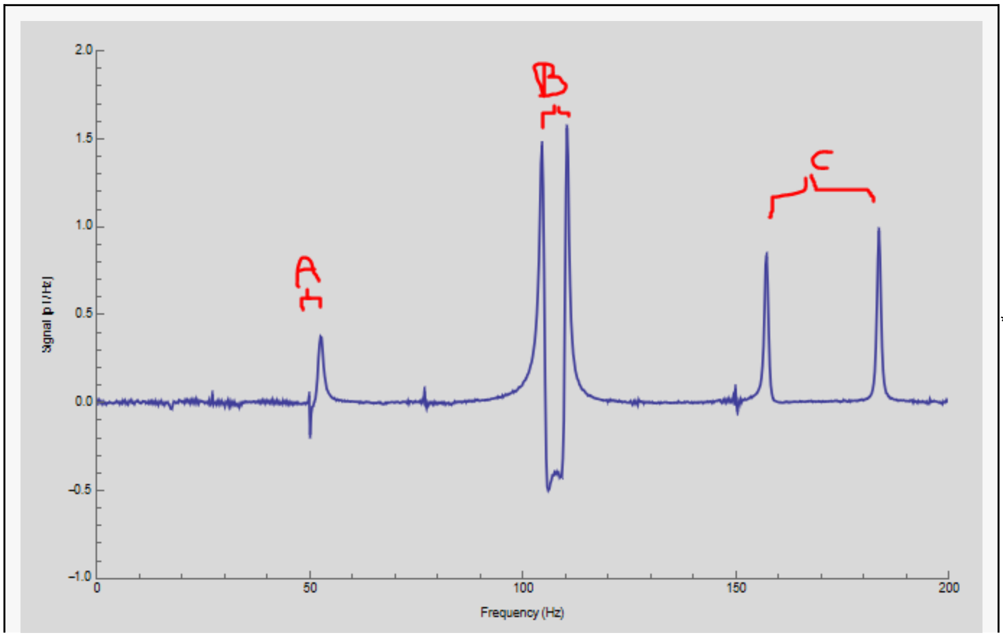 *)

2/15/24 17:20:05 In[]:=

```
Manipulate[ListPlot[{{#[[1]], Re[e^(-i α °) #[[2]]]} & /@ dataFT[k, 2, n, o][[ ;; ;; s]]},
    PlotRange → {{0, 200}, 4 {-0.5, 1}},
    FrameLabel → {"Frequency (Hz)", "Signal (pT/Hz)"}], {α, -360, 360}] // Quiet;
```

(*dataFT[k,3,n,o] it's only dropping initial points and converting to field units, but no zero filling, no phasing, no baseline correction *)

*dataFT[k,4,n,o] it's dropping initial points, removing moving average and removing time-domain fit, and converting to field units (i.e. no zero filling, no phasing, no frequency-baseline correction, no bandpass filter *)

dataFT[k,5,n,o] it's everything except phase correction)

2/15/24 17:20:05 In[]:=

```
dataFT[k, 3, n, o] =
  fftR@({#[[1]], 1/teslaConv #[[2]]} & /@ data[k, 1, n, o][[tacq0 * rate ;; (tacq0 + tacq) * rate]]);
dataFT[k, 4, n, o] = fftR@({#[[1]], 1/teslaConv #[[2]]} & /@ data[k, 3, n, o]);

dataFT[k, 5, n, o] = Table[{dataFTnoPhase[k, 1, n, o][[i, 1]], dataFTnoPhase[k, 1, n, o][[i, 2]] -
      (interdatnoPhase[k, 2, 1, n, o][phOrder][dataFTnoPhase[k, 1, n, o][[i, 1]]] +
       i interdatnoPhase[k, 2, 2, n, o][phOrder][dataFTnoPhase[k, 1, n, o][[i, 1]]])},
    {i, 1, Length@dataFTnoPhase[k, 1, n, o]}] // Quiet;
```

1/23/24 15:08:39 In[]:=

```
β = 0 °;
s = 1;
range = {{0, 200}, 1 {-1, 2}};
l = 1; p = 2;
rate = 1000;
tacq0 = 0.05; tacq = 2.048;
MA = 0.025;
ZF = tacq; LB = 0.;
k = 3; kmax = 3;
n = 1;
o = 1;
ν1 = 20;
ν2 = 1000;
order = 3;
```

1/23/24 15:08:41 In[]:=

```
Table[dataFT[κ, μ, 1, ω] = {Re@#[[1]], #[[2]]} & /@ (aux[1][[ω, μ, κ - 2]]),
  {ω, 1, 1}, {μ, 1, 2}, {κ, 3, 3}];
```

1/23/24 15:08:42 In[]:=

```
ListPlot[{
   {#[[1]], Re[e^(i β) #[[2]]]} & /@ dataFT[k, 2, n, o][[ ;; ;; s]] (*,
   {#[[1]],Im[e^(i β)#[[2]]]}&/@dataFT[k,2,n,o][[;;;;s]],
   {#[[1]],Abs[e^(i β)#[[2]]]}&/@dataFT[k,2,n,o][[;;;;s]]*)
  }
  ,
  PlotRange → range, FrameLabel → {"Frequency (Hz)", "Signal (pT/Hz)"},
  Frame → {True, True, False, False}] // Rasterize
```

1/23/24 15:08:43 Out[]=

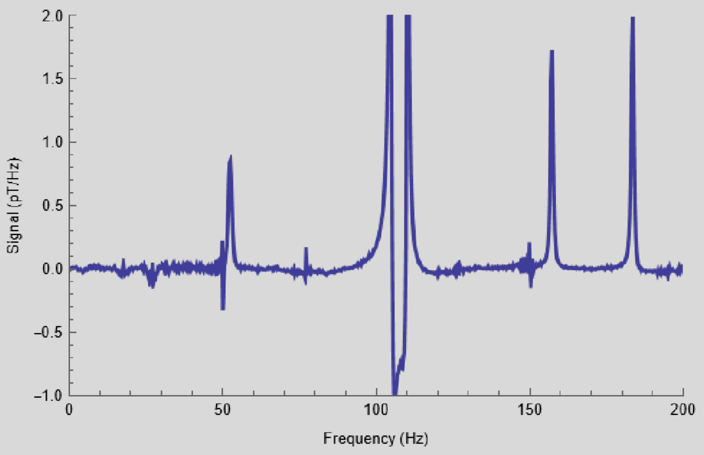

(*dataFit[k,m,n,o,r] where r determines the frequency range. r=1 is group A ranging 48-60 Hz. r=2 is group B ranging 90-120 Hz.r=3 ranges 151-190 Hz. I do all of them on the same cell*)

1/23/24 15:08:44 In[]:=

```
m = 2; r = 1;
Clear[a, ν, A, f, b, ϕ, g, gmax, model];
```

```
dataFit[k, m, n, o, r] = Join[
   Select[dataFT[k, m, n, o], 48 < #[[1]] < 60 &]
  ];
gmax = 0; lines = 50 {1};
model = (*∑_{i=0}^{0} A[i] e^(-v/τ[i]) +*) ∑_{i=1}^{1} a[i] e^(i ϕ[i]) ComplexLorentzian[v, 50(*v0[i]*), Γ[i]] +
    ∑_{i=2}^{2} a[i] e^(i ϕ[i]) ComplexLorentzian[v, v0[i], Γ[i]] + ∑_{i=0}^{gmax} g[i] v^i;
parameters[k, 3, 1, n, o, r] = {a[1], ϕ[1], (*{v0[1],105},*)
     Γ[1], a[2], ϕ[2], {v0[2], 52.4}, Γ[2]} ~ Join ~ (g /@ Range[0, gmax]);
fit[k, 3, 1, n, o, r] = NonlinearModelFit[
     {#[[1]], Re[#[[2]]]} & /@ dataFit[k, m, n, o, r],
     Re@model,
     parameters[k, 3, 1, n, o, r], v, MaxIterations → 1000] // Quiet;
fit[k, 3, 1, n, o, r]["RSquared"] // Quiet
fit[k, 3, 1, n, o, r]["ParameterTable"] // Quiet
s = 1; Show[{
   ListPlot[{{#[[1]], Re[e^(i β) #[[2]]]} & /@ dataFT[k, m, n, o][[;; ;; s]]},
    PlotRange → {{0, 200}, 2 {-0.5, 1}}, FrameLabel → {"Frequency (Hz)", "Signal (pT/Hz)"}],
   Plot[fit[k, 3, 1, n, o, r][v],
    {v, dataFit[k, m, n, o, r][[1, 1]], dataFit[k, m, n, o, r][[-1, 1]]},
    PlotRange → {{dataFit[k, m, n, o, r][[1, 1]], dataFit[k, m, n, o, r][[-1, 1]]}, All},
    PlotStyle → {Red, Opacity@0.5}]
  }] // Rasterize



r = 2;
Clear[a, v, A, f, b, ϕ, g, gmax, model];
dataFit[k, m, n, o, r] = Join[
   Select[dataFT[k, m, n, o], 90 < #[[1]] < 120 &]
  ];
gmax = 0; lines = 50 {1};
model = (*∑_{i=0}^{0} A[i] e^(-v/τ[i]) +*) ∑_{i=1}^{1} a[i] e^(i ϕ[i]) ComplexLorentzian[v, v0[i], Γ[i]] +
    ∑_{i=2}^{2} a[i] e^(i ϕ[i]) ComplexLorentzian[v, v0[i], Γ[i]] + ∑_{i=0}^{gmax} g[i] v^i;
parameters[k, 3, 1, n, o, r] = {a[1], ϕ[1], {v0[1], 105},
     Γ[1], a[2], ϕ[2], {v0[2], 110}, Γ[2]} ~ Join ~ (g /@ Range[0, gmax]);
fit[k, 3, 1, n, o, r] = NonlinearModelFit[
     {#[[1]], Re[#[[2]]]} & /@ dataFit[k, m, n, o, r],
     Re@model,
     parameters[k, 3, 1, n, o, r], v, MaxIterations → 1000] // Quiet;
fit[k, 3, 1, n, o, r]["RSquared"] // Quiet
fit[k, 3, 1, n, o, r]["ParameterTable"] // Quiet
s = 1; Show[{
   ListPlot[{{#[[1]], Re[e^(i β) #[[2]]]} & /@ dataFT[k, m, n, o][[;; ;; s]]},
    PlotRange → {{0, 200}, 2 {-0.5, 1}}, FrameLabel → {"Frequency (Hz)", "Signal (pT/Hz)"}],
   Plot[fit[k, 3, 1, n, o, r][v],
    {v, dataFit[k, m, n, o, r][[1, 1]], dataFit[k, m, n, o, r][[-1, 1]]},
    PlotRange → {{dataFit[k, m, n, o, r][[1, 1]], dataFit[k, m, n, o, r][[-1, 1]]}, All},
    PlotStyle → {Red, Opacity@0.5}]
```

```
  }] // Rasterize


r = 3;
Clear[a, ν, A, f, b, ϕ, g, gmax, model];
dataFit[k, m, n, o, r] = Join[
   Select[dataFT[k, m, n, o], 151 < #〚1〛 < 190 &]
  ];
gmax = 0; lines = 50 {1};
model = (*∑_{i=0}^{0} A[i] ⅇ^(-ν/τ[i]) + *) ∑_{i=1}^{1} a[i] ⅇ^(ⅈ ϕ[i]) ComplexLorentzian[ν, ν0[i], Γ[i]] +
   ∑_{i=2}^{2} a[i] ⅇ^(ⅈ ϕ[i]) ComplexLorentzian[ν, ν0[i], Γ[i]] + ∑_{i=0}^{gmax} g[i] ν^i;
parameters[k, 3, 1, n, o, r] = {a[1], ϕ[1], {ν0[1], 157},
    Γ[1], a[2], ϕ[2], {ν0[2], 183}, Γ[2]} ~ Join ~ (g /@ Range[0, gmax]);
fit[k, 3, 1, n, o, r] = NonlinearModelFit[
    {#〚1〛, Re[#〚2〛]} & /@ dataFit[k, m, n, o, r],
    Re@model,
    parameters[k, 3, 1, n, o, r], ν, MaxIterations → 1000] // Quiet;
fit[k, 3, 1, n, o, r]["RSquared"] // Quiet
fit[k, 3, 1, n, o, r]["ParameterTable"] // Quiet
s = 1; Show[{
   ListPlot[{{#〚1〛, Re[ⅇ^(ⅈ β) #〚2〛]} & /@ dataFT[k, m, n, o]〚 ;; ;; s〛},
    PlotRange → {{0, 200}, 2 {-0.5, 1}}, FrameLabel → {"Frequency (Hz)", "Signal (pT/Hz)"}],
   Plot[fit[k, 3, 1, n, o, r][ν],
    {ν, dataFit[k, m, n, o, r]〚1, 1〛, dataFit[k, m, n, o, r]〚-1, 1〛},
    PlotRange → {{dataFit[k, m, n, o, r]〚1, 1〛, dataFit[k, m, n, o, r]〚-1, 1〛}, All},
    PlotStyle → {Red, Opacity@0.5}]
  }] // Rasterize
```

1/23/24 15:08:44 Out[]=

0.992165

1/23/24 15:08:44 Out[]=

	Estimate	Standard Error	t-Statistic	P-Value
a[1]	0.105344	0.0228842	4.60335	0.0000397722
ϕ[1]	4.4008	0.333948	13.1781	2.4983×10^{-16}
Γ[1]	0.132559	0.0560231	2.36614	0.0227822
a[2]	1.91937	0.0540584	35.5055	2.06323×10^{-32}
ϕ[2]	0.0170666	0.0251427	0.67879	0.501085
ν0[2]	52.349	0.0198231	2640.8	7.30696×10^{-109}
Γ[2]	1.29027	0.0422328	30.5513	7.85071×10^{-30}
g[0]	−0.0223064	0.00560845	−3.97728	0.000276923

1/23/24 15:08:45 Out[]=

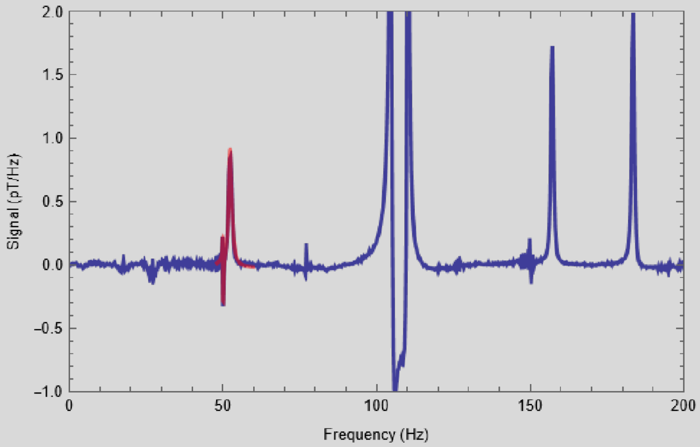

1/23/24 15:08:45 Out[]=

0.994146

1/23/24 15:08:45 Out[]=

	Estimate	Standard Error	t-Statistic	P-Value
a[1]	−7.0967	0.104218	−68.0946	3.50905×10^{-94}
ϕ[1]	−5.43437	0.0140974	−385.488	1.95917×10^{-179}
ν0[1]	104.79	0.0107174	9777.61	0.
Γ[1]	−1.15789	0.0218701	−52.944	4.19467×10^{-82}
a[2]	6.07326	0.0948793	64.0104	3.38898×10^{-91}
ϕ[2]	0.534823	0.0151024	35.4131	2.37748×10^{-63}
ν0[2]	110.108	0.00974156	11302.9	0.
Γ[2]	0.981354	0.0198365	49.472	6.94724×10^{-79}
g[0]	−0.0887099	0.00739059	−12.0031	5.89315×10^{-22}

1/23/24 15:08:46 Out[]=

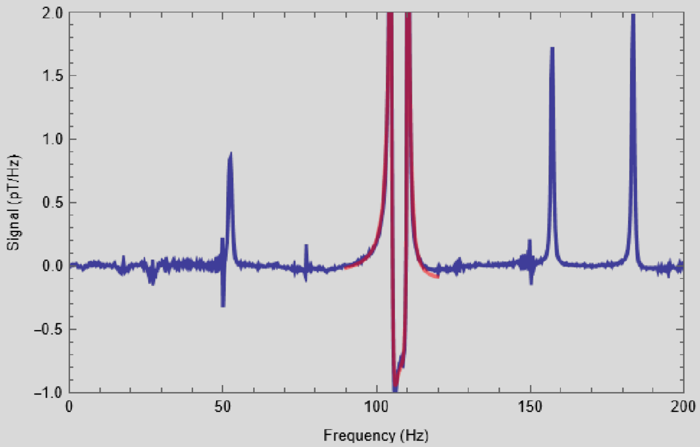

1/23/24 15:08:46 Out[]=

0.995668

1/23/24 15:08:46 Out[]=

	Estimate	Standard Error	t-Statistic	P-Value
a[1]	2.77238	0.0342735	80.89	3.08445×10^{-126}
ϕ[1]	−0.0134829	0.0124361	−1.08418	0.280013
v0[1]	157.187	0.00837451	18 769.8	0.
Γ[1]	0.995063	0.016571	60.0483	2.71962×10^{-107}
a[2]	3.11349	0.034075	91.3716	4.55082×10^{-134}
ϕ[2]	−0.101594	0.0107661	−9.43646	6.6499×10^{-17}
v0[2]	183.554	0.00712369	25 766.7	0.
Γ[2]	0.969852	0.0142416	68.0998	2.99029×10^{-115}
g[0]	−0.0206756	0.00247463	−8.35502	3.94207×10^{-14}

1/23/24 15:08:47 Out[]=

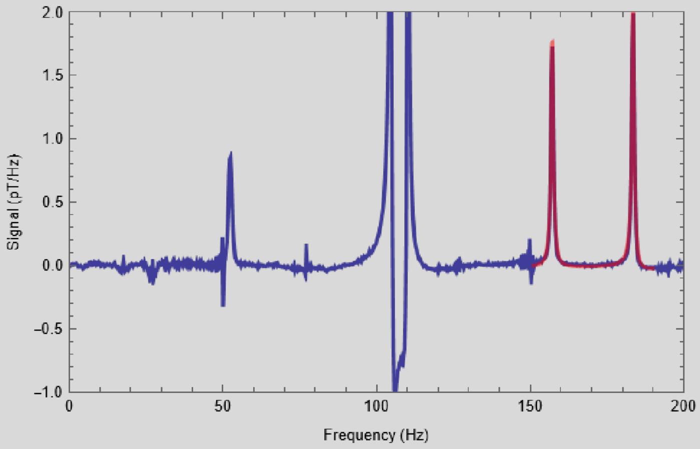

1/23/24 15:08:48 In[]:=

```
Table[fit[k, 3, 1, n, o, r]["ParameterTable"] // Quiet, {r, 1, 3}] // Row
{(fit[k, 3, 1, n, o, 1]["ParameterTable"])[[1, 1, 7, 1 ;; 3]] // Quiet,
  (fit[k, 3, 1, n, o, 2]["ParameterTable"])[[1, 1, 4, 1 ;; 3]] // Quiet,
  (fit[k, 3, 1, n, o, 2]["ParameterTable"])[[1, 1, 8, 1 ;; 3]] // Quiet,
  (fit[k, 3, 1, n, o, 3]["ParameterTable"])[[1, 1, 4, 1 ;; 3]] // Quiet,
  (fit[k, 3, 1, n, o, 3]["ParameterTable"])[[1, 1, 8, 1 ;; 3]] // Quiet
 } // Column
```

1/23/24 15:08:49 Out[]=

	Estimate	Standard Error	t-Statistic	P-Value
a[1]	0.105344	0.0228842	4.60335	0.0000397722
ϕ[1]	4.4008	0.333948	13.1781	2.4983×10^{-16}
Γ[1]	0.132559	0.0560231	2.36614	0.0227822
a[2]	1.91937	0.0540584	35.5055	2.06323×10^{-32}
ϕ[2]	0.0170666	0.0251427	0.67879	0.501085
ν0[2]	52.349	0.0198231	2640.8	7.30696×10^{-109}
Γ[2]	1.29027	0.0422328	30.5513	7.85071×10^{-30}
g[0]	−0.0223064	0.00560845	−3.97728	0.000276923

	Estimate	Standard Error	t-Statistic	P-Value
a[1]	−7.0967	0.104218	−68.0946	3.50905×10^{-94}
ϕ[1]	−5.43437	0.0140974	−385.488	1.95917×10^{-179}
ν0[1]	104.79	0.0107174	9777.61	0.
Γ[1]	−1.15789	0.0218701	−52.944	4.19467×10^{-82}
a[2]	6.07326	0.0948793	64.0104	3.38898×10^{-91}
ϕ[2]	0.534823	0.0151024	35.4131	2.37748×10^{-63}
ν0[2]	110.108	0.00974156	11302.9	0.
Γ[2]	0.981354	0.0198365	49.472	6.94724×10^{-79}
g[0]	−0.0887099	0.00739059	−12.0031	5.89315×10^{-22}

	Estimate	Standard Error	t-Statistic	P-Value
a[1]	2.77238	0.0342735	80.89	3.08445×10^{-126}
ϕ[1]	−0.0134829	0.0124361	−1.08418	0.280013
ν0[1]	157.187	0.00837451	18769.8	0.
Γ[1]	0.995063	0.016571	60.0483	2.71962×10^{-107}
a[2]	3.11349	0.034075	91.3716	4.55082×10^{-134}
ϕ[2]	−0.101594	0.0107661	−9.43646	6.6499×10^{-17}
ν0[2]	183.554	0.00712369	25766.7	0.
Γ[2]	0.969852	0.0142416	68.0998	2.99029×10^{-115}
g[0]	−0.0206756	0.00247463	−8.35502	3.94207×10^{-14}

1/23/24 15:08:51 Out[]=

```
{ν0[2], 52.349, 0.0198231}
{ν0[1], 104.79, 0.0107174}
{ν0[2], 110.108, 0.00974156}
{ν0[1], 157.187, 0.00837451}
{ν0[2], 183.554, 0.00712369}
```

1/23/24 15:08:52 In[]:=

```
col = {Orange, Red, Yellow}; s = 1;
plotReviewer1 = Show[{
    ListPlot[{{#[[1]], Re[e^(i β) #[[2]]]} & /@ dataFT[k, 2, n, o][[;; ;; s]]},
     PlotRange → {{0, 200}, 4 {-0.5, 1}}, FrameLabel → {"Frequency (Hz)", "Signal (pT/Hz)"}],
    Table[Plot[
      fit[k, 3, 1, n, o, r][ν]
      , {ν, dataFit[k, m, n, o, r][[1, 1]], dataFit[k, m, n, o, r][[-1, 1]]},
      PlotRange → {{dataFit[k, m, n, o, r][[1, 1]], dataFit[k, m, n, o, r][[-1, 1]]}, All},
      PlotStyle → {col[[r]], Opacity@0.3}
     ], {r, 1, 3}]}];
plotReviewer1 // Rasterize
Export[FileNameJoin[{outputFolder, "plotReviewer1.pdf"}], plotReviewer1];
Export[FileNameJoin[{outputFolder, "plotReviewer1.png"}], plotReviewer1];
```

1/23/24 15:08:53 Out[]=

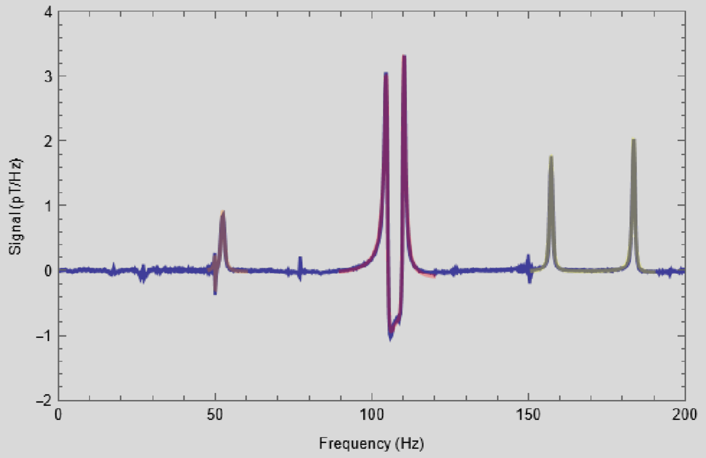

1/24/24 12:34:57 In[]:=

```
Jcoup[k, 3, 1, 1] // MatrixForm
```

1/24/24 12:34:57 Out[]//MatrixForm=

52.3766	0.0195332
104.779	0.011004
110.102	0.0100829
157.169	0.00896677
183.544	0.00789176

For different partitions

2/21/24 11:38:53 In[]:=

```
Clear[data];
datafolderMain = "T:\\Projects\\ZULF NMR\\Roman\\ZULF\\Papers\\Quadrupole
    project\\Phase IV Data\\Experiment 1 - Thermal spectra\\Partitions";
l = 1;
p = 2;
```

Remove 0.05 s initial data ⇒ 5000*0.05=250 points. m stores is all the aux variables I need data[i,m]. Take every l element. s is less

points for the plots. 5000*1.024=5120 points less for 3.072 s
p is channel. data[k,m,n,o] n is for folders and o for scans

2/21/24 11:38:55 In[]:=

```
β = 0 °;
s = 1;
range = {{0, 200}, 1 {-1, 2}};
rate = 1000;
tacq0 = 0.05; tacq = 2.048;
MA = 0.025;
ZF = tacq; LB = 0.;
k = 3; kmax = 3;
n = 1;
o = 1;
ν1 = 20;
ν2 = 1000;
order = 3;
```

2/21/24 11:38:56 In[]:=

```
datafolder = {
   FileNameJoin[{datafolderMain, "00001 partitions of 36000 scans"}],
   FileNameJoin[{datafolderMain, "00002 partitions of 18000 scans"}],
   FileNameJoin[{datafolderMain, "00003 partitions of 12000 scans"}],
   FileNameJoin[{datafolderMain, "00004 partitions of 09000 scans"}],
   FileNameJoin[{datafolderMain, "00012 partitions of 03000 scans"}],
   FileNameJoin[{datafolderMain, "00036 partitions of 01000 scans"}],
   FileNameJoin[{datafolderMain, "00060 partitions of 00600 scans"}],
   FileNameJoin[{datafolderMain, "00360 partitions of 00100 scans"}],
   FileNameJoin[{datafolderMain, "01000 partitions of 00036 scans"}],
   FileNameJoin[{datafolderMain, "02250 partitions of 00016 scans"}],
   FileNameJoin[{datafolderMain, "03600 partitions of 00010 scans"}],
   FileNameJoin[{datafolderMain, "04500 partitions of 00008 scans"}],
   FileNameJoin[{datafolderMain, "09000 partitions of 00004 scans"}],
   FileNameJoin[{datafolderMain, "18000 partitions of 00002 scans"}],
   FileNameJoin[{datafolderMain, "36000 partitions of 00001 scans"}]
  };
outfiles = {
   FileNameJoin[{datafolderMain, "00001 partitions of 36000 scans loop.h5"}],
   FileNameJoin[{datafolderMain, "00002 partitions of 18000 scans loop.h5"}],
   FileNameJoin[{datafolderMain, "00003 partitions of 12000 scans loop.h5"}],
   FileNameJoin[{datafolderMain, "00004 partitions of 09000 scans loop.h5"}],
   FileNameJoin[{datafolderMain, "00012 partitions of 03000 scans loop.h5"}],
   FileNameJoin[{datafolderMain, "00036 partitions of 01000 scans loop.h5"}],
   FileNameJoin[{datafolderMain, "00060 partitions of 00600 scans loop.h5"}],
   FileNameJoin[{datafolderMain, "00360 partitions of 00100 scans loop.h5"}],
   FileNameJoin[{datafolderMain, "01000 partitions of 00036 scans loop.h5"}],
   FileNameJoin[{datafolderMain, "02250 partitions of 00016 scans loop.h5"}],
   FileNameJoin[{datafolderMain, "03600 partitions of 00010 scans loop.h5"}],
   FileNameJoin[{datafolderMain, "04500 partitions of 00008 scans loop.h5"}],
   FileNameJoin[{datafolderMain, "09000 partitions of 00004 scans loop.h5"}],
   FileNameJoin[{datafolderMain, "18000 partitions of 00002 scans loop.h5"}],
```

```
    FileNameJoin[{datafolderMain, "36000 partitions of 00001 scans loop.h5"}]
   };
outfilesReviewers = {
    FileNameJoin[
     {datafolderMain, "Answer to Reviewers v2", "00001 partitions of 36000 scans loop.h5"}],
    FileNameJoin[
     {datafolderMain, "Answer to Reviewers v2", "00002 partitions of 18000 scans loop.h5"}],
    FileNameJoin[
     {datafolderMain, "Answer to Reviewers v2", "00003 partitions of 12000 scans loop.h5"}],
    FileNameJoin[
     {datafolderMain, "Answer to Reviewers v2", "00004 partitions of 09000 scans loop.h5"}],
    FileNameJoin[
     {datafolderMain, "Answer to Reviewers v2", "00012 partitions of 03000 scans loop.h5"}],
    FileNameJoin[
     {datafolderMain, "Answer to Reviewers v2", "00036 partitions of 01000 scans loop.h5"}],
    FileNameJoin[
     {datafolderMain, "Answer to Reviewers v2", "00060 partitions of 00600 scans loop.h5"}],
    FileNameJoin[
     {datafolderMain, "Answer to Reviewers v2", "00360 partitions of 00100 scans loop.h5"}],
    FileNameJoin[
     {datafolderMain, "Answer to Reviewers v2", "01000 partitions of 00036 scans loop.h5"}],
    FileNameJoin[
     {datafolderMain, "Answer to Reviewers v2", "02250 partitions of 00016 scans loop.h5"}],
    FileNameJoin[
     {datafolderMain, "Answer to Reviewers v2", "03600 partitions of 00010 scans loop.h5"}],
    FileNameJoin[
     {datafolderMain, "Answer to Reviewers v2", "04500 partitions of 00008 scans loop.h5"}],
    FileNameJoin[
     {datafolderMain, "Answer to Reviewers v2", "09000 partitions of 00004 scans loop.h5"}],
    FileNameJoin[
     {datafolderMain, "Answer to Reviewers v2", "18000 partitions of 00002 scans loop.h5"}],
    FileNameJoin[
     {datafolderMain, "Answer to Reviewers v2", "36000 partitions of 00001 scans loop.h5"}]
   };

outfilesReviewersEVD = Table[{
     FileNameJoin[{datafolderMain, "Answer to Reviewers v2EVD [" <> ToString@v <> "]",
       "00001 partitions of 36000 scans loop.h5"}],
     FileNameJoin[{datafolderMain, "Answer to Reviewers v2EVD [" <> ToString@v <> "]",
       "00002 partitions of 18000 scans loop.h5"}],
     FileNameJoin[{datafolderMain, "Answer to Reviewers v2EVD [" <> ToString@v <> "]",
       "00003 partitions of 12000 scans loop.h5"}],
     FileNameJoin[{datafolderMain, "Answer to Reviewers v2EVD [" <> ToString@v <> "]",
       "00004 partitions of 09000 scans loop.h5"}],
     FileNameJoin[{datafolderMain, "Answer to Reviewers v2EVD [" <> ToString@v <> "]",
       "00012 partitions of 03000 scans loop.h5"}],
     FileNameJoin[{datafolderMain, "Answer to Reviewers v2EVD [" <> ToString@v <> "]",
       "00036 partitions of 01000 scans loop.h5"}],
     FileNameJoin[{datafolderMain, "Answer to Reviewers v2EVD [" <> ToString@v <> "]",
       "00060 partitions of 00600 scans loop.h5"}],
     FileNameJoin[{datafolderMain, "Answer to Reviewers v2EVD [" <> ToString@v <> "]",
```

```
    "00360 partitions of 00100 scans loop.h5"}],
   FileNameJoin[{datafolderMain, "Answer to Reviewers v2EVD [" <> ToString@v <> "]",
    "01000 partitions of 00036 scans loop.h5"}],
   FileNameJoin[{datafolderMain, "Answer to Reviewers v2EVD [" <> ToString@v <> "]",
    "02250 partitions of 00016 scans loop.h5"}],
   FileNameJoin[{datafolderMain, "Answer to Reviewers v2EVD [" <> ToString@v <> "]",
    "03600 partitions of 00010 scans loop.h5"}],
   FileNameJoin[{datafolderMain, "Answer to Reviewers v2EVD [" <> ToString@v <> "]",
    "04500 partitions of 00008 scans loop.h5"}],
   FileNameJoin[{datafolderMain, "Answer to Reviewers v2EVD [" <> ToString@v <> "]",
    "09000 partitions of 00004 scans loop.h5"}],
   FileNameJoin[{datafolderMain, "Answer to Reviewers v2EVD [" <> ToString@v <> "]",
    "18000 partitions of 00002 scans loop.h5"}],
   FileNameJoin[{datafolderMain, "Answer to Reviewers v2EVD [" <> ToString@v <> "]",
    "36000 partitions of 00001 scans loop.h5"}]
  }, {v, 3}];
nmax = 8; mmax = 7; mmaxEVD = 6; vmax = 3; mmaxTot = mmax + vmax * mmaxEVD;
omax = {1, 2, 3, 4, 12, 36, 60, 360, 1000, 2250, 3600, 4500, 9000, 18000, 36000};
```

Preliminary: Read original files from partitions folders

(*There is a bug for scans over 10000 due to IntegerString o, 10, 4*)

2/15/24 17:20:07 In[]:=

```
Clear@data
SetSharedFunction[data];
```

2/15/24 17:20:07 In[]:=

```
n = 1; m = 1; nmax
Print[
  "Expected compiling time = " <> ToString[NumberForm[4/20 * Sum[omax[[n]] / 60, {n, 1, nmax}], 3] // N] <> " min"];
Monitor[
  (*Parallel*)Table[
   data[1, 1, n, o] = ToExpression@ReplaceStringR@
      Import[FileNameJoin[{datafolder[[n]], "Scan-" <> IntegerString[o, 10, 4] <> ".lvm"}],
       "Table", NumberPoint → ",", "FieldSeparators" → "\t"][[23 ;; -2 ;; 1, {1, 3}]];
   data[2, 1, n, o] = ToExpression@ReplaceStringR@
      Import[FileNameJoin[{datafolder[[n]], "Scan-" <> IntegerString[o, 10, 4] <> ".lvm"}],
       "Table", NumberPoint → ",", "FieldSeparators" → "\t"][[23 ;; -2 ;; 1, {1, 5}]];
   data[3, 1, n, o] = data[2, 1, n, o] - data[1, 1, n, o].DiagonalMatrix[{0, 1}];
   , {n, nmax}, {o, omax[[n]]}];
 , "{n,o} = " <> ToString@{n, o} <> " of " <> ToString@omax[[n]] <> " scans"];
```

2/15/24 17:20:07 Out[]=

```
2
```

```
Expected compiling time = 0.01 min
```

1st loop: export processed files

2/15/24 17:20:08 In[]:=

```
q = 1; (*Downsize at export*)
np = 850; (*Take until ~207 Hz*)
teslaConv = 0.9×10^-3 (*Volts per pT*);
n = 1; Print["nmax=" <> ToString@nmax <> "   mmax  =  " <> ToString@mmax]

phOrder = 2;
interdatph[phOrder_] := Interpolation[
    {{52.3925, 0. °}, {Mean[{104.784, 110.115}], 165 °}, {157.171, 245 °}, {183.555, 315 °}}
    , InterpolationOrder → phOrder, Method → "Spline"] // Quiet;
interdat150drop[phOrder_] :=
  Interpolation[{{52.3925, 48 °}, {Mean[{104.784, 110.115}], 203 °}, {157.171, 88 ° + 360 °},
     {183.555, 12 ° + 360 °}}, InterpolationOrder → phOrder, Method → "Spline"] // Quiet;
(*Eventhough most probably I will not use this*)


{fit[k, 1, 1, 1], fit150drop[k, 1, 1, 1], interdat[k, 2, 1, 1, 1][phOrder],
  interdat[k, 2, 2, 1, 1][phOrder], interdatnoPhase[k, 2, 1, 1, 1][phOrder],
  interdatnoPhase[k, 2, 2, 1, 1][phOrder]} // Column
```

```
dropRange = tacq0 * rate ;; (tacq0 + tacq) * rate;
Print["Expected compiling time = " <>
  ToString[NumberForm[5.4 / (Sum[omax[[n]], {n, 1, 2}]) * Sum[omax[[n]] / 60, {n, 1, nmax}], 3] // N] <> " min"];
Clear@aux;
Monitor[
  Table[
    Table[
      data[k, 2, n, o] =
       data[k, 1, n, o] - MovingAverageR[data[k, 1, n, o], MA, rate].DiagonalMatrix[{0, 1}];
      aux = data[k, 2, n, o][[dropRange]];
      aux[[ ;; , 2]] = aux[[ ;; , 2]] - fit[k, 1, 1, 1] /@ (aux[[ ;; , 1]]);
      data[k, 3, n, o] = aux; Clear@aux;

      aux = data[k, 2, n, o][[0.15 * rate ;;]];
      aux[[ ;; , 2]] = aux[[ ;; , 2]] - fit150drop[k, 1, 1, 1] /@ (aux[[ ;; , 1]]);
      data150drop[k, 3, n, o] = aux; Clear@aux;
      data[k, 4, n, o] = BandpassR[data[k, 3, n, o], ν1, ν2, order];
      data150drop[k, 4, n, o] = BandpassR[data150drop[k, 3, n, o], ν1, ν2, order];
      data[k, 5, n, o] = {#[[1]], 1/teslaConv e^(-LB #[[1]]) #[[2]]} & /@ Join[
         data[k, 4, n, o],
         Table[{data[k, 4, n, o][[-1, 1]] + i/rate, 0.}, {i, 1, ZF * rate}]
       ];
```

```
datanoZF[k, 5, n, o] = {#[[1]], 1/teslaConv ⅇ^(-LB #[[1]]) #[[2]]} & /@ data[k, 4, n, o];
data150drop[k, 5, n, o] = {#[[1]], 1/teslaConv ⅇ^(-LB #[[1]]) #[[2]]} & /@ Join[
   data150drop[k, 4, n, o],
   Table[{data150drop[k, 4, n, o][[-1, 1]] + i/rate, 0.}, {i, 1, ZF * rate}]
  ];
dataFT[k, 1, n, o] =
  {#[[1]], ⅇ^(-ⅈ (interdatph[phOrder][#[[1]]])) #[[2]]} & /@ fftR[data[k, 5, n, o]] // Quiet;
dataFTnoPhase[k, 1, n, o] = {#[[1]], ⅇ^(-ⅈ 0) #[[2]]} & /@ fftR[data[k, 5, n, o]] // Quiet;
dataFTnoZF[k, 1, n, o] =
  {#[[1]], ⅇ^(-ⅈ (interdatph[phOrder][#[[1]]])) #[[2]]} & /@ fftR[datanoZF[k, 5, n, o]] // Quiet;

(*/////////Using Interpolation/////////*)
aux = dataFT[k, 1, n, o]; (*much faster this way*)
aux[[;; , 2]] = aux[[;; , 2]] - (interdat[k, 2, 1, 1, 1][phOrder][aux[[;; , 1]]] +
     ⅈ interdat[k, 2, 2, 1, 1][phOrder][aux[[;; , 1]]]) // Quiet;
dataFT[k, 2, n, o] = aux;
Clear@aux;
(*///////// Answers to Reviewers/////////*)
dataFT[k, 3, n, o] =
  (fftR@({#[[1]], 1/teslaConv #[[2]]} & /@ data[k, 1, n, o][[dropRange]]));
dataFT[k, 4, n, o] = (fftR@({#[[1]], 1/teslaConv #[[2]]} & /@ data[k, 3, n, o]));
aux = dataFTnoPhase[k, 1, n, o]; (*much faster this way*)
aux[[;; , 2]] = aux[[;; , 2]] - (interdatnoPhase[k, 2, 1, 1, 1][phOrder][aux[[;; , 1]]] +
     ⅈ interdatnoPhase[k, 2, 2, 1, 1][phOrder][aux[[;; , 1]]]) // Quiet;
dataFT[k, 5, n, o] = aux;
Clear@aux;

aux = dataFTnoZF[k, 1, n, o]; (*much faster this way*)
aux[[;; , 2]] = aux[[;; , 2]] - (interdat[k, 2, 1, 1, 1][phOrder][aux[[;; , 1]]] +
     ⅈ interdat[k, 2, 2, 1, 1][phOrder][aux[[;; , 1]]]) // Quiet;
dataFT[k, 6, n, o] = aux;
Clear@aux;

aux = dataFTnoZF[k, 1, n, o]; (*much faster this way*)
aux[[;; , 2]] = aux[[;; , 2]] - (interdat[k, 2, 1, 1, 1][phOrder][aux[[;; , 1]]] +
     ⅈ interdat[k, 2, 2, 1, 1][phOrder][aux[[;; , 1]]]) // Quiet;
dataFT[k, 6, n, o] = aux;
Clear@aux;

dataFT[k, 7, n, o] = {#[[1]], #[[2]]} & /@ fftR[data150drop[k, 5, n, o]] // Quiet;
(*/////////////////////////*)

, {o, omax[[n]]}];
```

```
    , {n, 1, nmax}];
  , "{n,o} = " <> ToString@{n, o} <> " of " <> ToString@omax[[n]] <> " scans"];
```

Expected compiling time = 0.09 min

2/15/24 17:20:14 In[]:=

```
Monitor[Table[Export[outfilesReviewers[[n]], Table[dataFT[k, m, n, o][[1 ;; np ;; q]],
      {o, omax[[n]]}, {m, mmax}, {k, 3, 3}], "ComplexKeys" → {"Re", "Im"}], {n, nmax}],
  "{n,o} = " <> ToString@{n, o} <> " of " <> ToString@omax[[n]] <> " scans"];
```

Read EVD (a co-author) files from partitions folders

```
Clear[v, datafolderMainEVD, datafolderEVD];
v = 1; (*v is EVD's folder*)
datafolderMainEVD = {
   "T:\\Projects\\ZULF NMR\\Roman\\ZULF\\Papers\\Quadrupole project\\Phase
     IV Data\\Erik Processing\\EVD Partitioned Data\\Spline and MA v2",
   "T:\\Projects\\ZULF NMR\\Roman\\ZULF\\Papers\\Quadrupole
     project\\Phase IV Data\\Erik Processing\\EVD Partitioned Data\\Spline v2",
   "T:\\Projects\\ZULF NMR\\Roman\\ZULF\\Papers\\Quadrupole
     project\\Phase IV Data\\Erik Processing\\EVD Partitioned Data\\MA v2"
  };
datafolderEVD = Table[{
     FileNameJoin[{datafolderMainEVD[[v]], "1 partitions of 36000 scans"}],
     FileNameJoin[{datafolderMainEVD[[v]], "2 partitions of 18000 scans"}],
     FileNameJoin[{datafolderMainEVD[[v]], "3 partitions of 12000 scans"}],
     FileNameJoin[{datafolderMainEVD[[v]], "4 partitions of 9000 scans"}],
     FileNameJoin[{datafolderMainEVD[[v]], "12 partitions of 3000 scans"}],
     FileNameJoin[{datafolderMainEVD[[v]], "36 partitions of 1000 scans"}],
     FileNameJoin[{datafolderMainEVD[[v]], "60 partitions of 600 scans"}],
     FileNameJoin[{datafolderMainEVD[[v]], "360 partitions of 100 scans"}],
     FileNameJoin[{datafolderMainEVD[[v]], "1000 partitions of 36 scans"}],
     FileNameJoin[{datafolderMainEVD[[v]], "2250 partitions of 16 scans"}],
     FileNameJoin[{datafolderMainEVD[[v]], "3600 partitions of 10 scans"}],
     FileNameJoin[{datafolderMainEVD[[v]], "4500 partitions of 8 scans"}],
     FileNameJoin[{datafolderMainEVD[[v]], "9000 partitions of 4 scans"}],
     FileNameJoin[{datafolderMainEVD[[v]], "18000 partitions of 2 scans"}],
     FileNameJoin[{datafolderMainEVD[[v]], "36000 partitions of 1 scans"}]
    }, {v, 1, 3}];
```

2/15/24 17:20:15 In[]:=

```
Monitor[Table[dataEVD[v][k, 1, n, o] =
      Import[FileNameJoin[{datafolderEVD[[v, n]], "scan " <> ToString@o <> ".txt"}], "Table"],
     {n, nmax}, {o, omax[[n]]}, {v, 1, 3}];, "{n,o,v} = " <>
    ToString@{n, o, v} <> " of " <> ToString@omax[[n]] <> " scans"];
```

2/15/24 17:20:16 In[]:=

```
aux = ;; ;
{dataEVD[1][k, 1, n, o][[aux]] // ListPlot,
  dataEVD[2][k, 1, n, o][[aux]] // ListPlot, dataEVD[3][k, 1, n, o][[aux]] // ListPlot} // Row
```

2/15/24 17:20:17 Out[]=

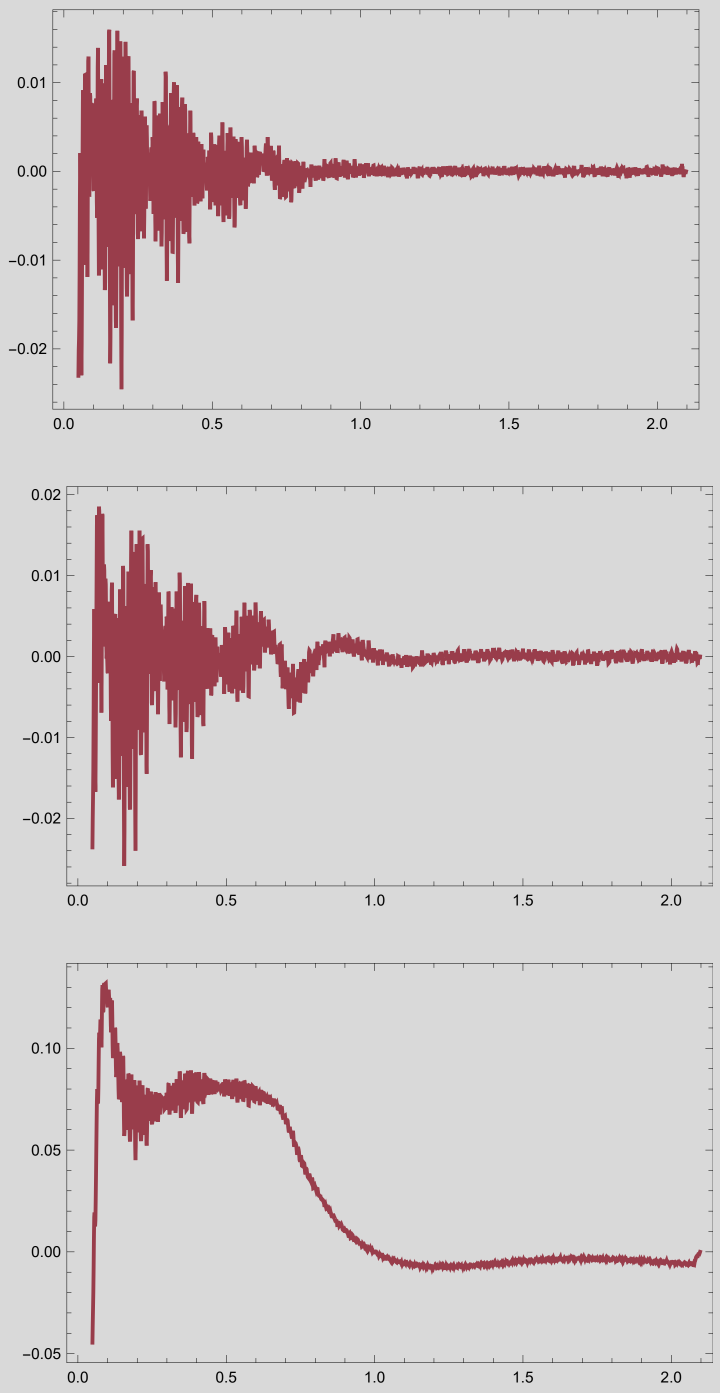

2/15/24 17:20:17 In[]:=

```
Manipulate[dataEVD[v][k, 1, n, o][[ ;; 10]] // MatrixForm, {n, 1, nmax, 1}, {o, 1, omax[[n]], 1}]
```

2/15/24 17:20:17 In[]:=

```
dp = 1 (*dropped points*);
Manipulate[ListPlot[{#[[1]], Re[ⅇ^(-ⅈ ϕ) #[[2]]]} & /@ fftR[dataEVD[v][k, 1, n, o][[dp ;;]]],
  PlotRange → {{0, 200}, 0.002 {-1, 1}}], {n, 1, nmax, 1},
 {o, 1, omax[[n]], 1}, {v, 1, vmax, 1}, {ϕ, 0 °, 360 °, 1 °}]
```

2/15/24 17:20:17 In[]:=

```
Clear[phaseInter, interdat, phOrder, interdatEVD, interdatEVD150drop];
phOrder = 2;
Table[interdatEVD[v][phOrder_] := Interpolation[
      {{52.3925, 0. °}, {Mean[{104.784, 110.115}], 147 °}, {157.171, 245 °}, {183.555, 315 °}}
      , InterpolationOrder → phOrder, Method → "Spline"] // Quiet;, {v, 1, 3, 1}];
Table[Show[{Plot[interdatEVD[v][phOrder][ν],
      {ν, 50, 200}, FrameLabel → {"Frequency (Hz)", "Phase (deg)"}] // Quiet,
    ListPlot[{{52.3925, 0. °}, {Mean[{104.784, 110.115}], 147 °}, {157.171, 245 °},
      {183.555, 315 °}}, Joined → False]}], {v, 1, 3, 1}] // Row
Table[interdatEVD150drop[v][phOrder_] := Interpolation[
      {{52.3925, 0. °}, {Mean[{104.784, 110.115}], 197 °}, {157.171, 95 °}, {183.555, 24 °}}
      , InterpolationOrder → phOrder, Method → "Spline"] // Quiet;, {v, 1, 3, 1}];
Table[Show[{Plot[interdatEVD150drop[v][phOrder][ν],
      {ν, 50, 200}, FrameLabel → {"Frequency (Hz)", "Phase (deg)"}] // Quiet,
    ListPlot[{{52.3925, 0. °}, {Mean[{104.784, 110.115}], 197 °}, {157.171, 95 °},
      {183.555, 24 °}}, Joined → False]}], {v, 1, 3, 1}] // Row
```

1st loop: export processed files for EVD files

```
q = 1;(*Downsize at export*)
np = 850;(*Take until ~207 Hz*)
teslaConv = 0.9×10^-3 (*Volts per pT conversion from the QuSpin*);
n = 1; Print["nmax=" <> ToString@nmax <> "  mmax = " <> ToString@mmax]

phOrder = 2;
```

nmax=2 mmax = 7

```
Clear@aux;
Print["Expected compiling time = " <>
  ToString[NumberForm[4.6 / (3 Sum[omax[[n]], {n, 1, 2}]) * vmax Sum[omax[[n]], {n, 1, nmax}] / 60, 3] // N] <> " min"];
Monitor[
  Table[
    Table[
      dataEVD[v][k, 3, n, o] = dataEVD[v][k, 1, n, o];
      dataEVD150drop[v][k, 3, n, o] = dataEVD[v][k, 1, n, o][[100 ;;]];

       (*No Bandpass filter*)
```

```
      dataEVD[v][k, 5, n, o] = {#[[1]], 1/teslaConv e^(-LB #[[1]]) #[[2]]} & /@ Join[
         dataEVD[v][k, 3, n, o],
         Table[{dataEVD[v][k, 3, n, o][[-1, 1]] + i/rate, 0.}, {i, 1, ZF*rate}]
        ];
      dataEVDnoZF[v][k, 5, n, o] = {#[[1]], 1/teslaConv e^(-LB #[[1]]) #[[2]]} & /@ dataEVD[v][k, 3, n, o];
      dataEVD150drop[v][k, 5, n, o] = {#[[1]], 1/teslaConv e^(-LB #[[1]]) #[[2]]} & /@ Join[
         dataEVD150drop[v][k, 3, n, o],
         Table[{dataEVD150drop[v][k, 3, n, o][[-1, 1]] + i/rate, 0.}, {i, 1, ZF*rate}]
        ];
      dataEVD150dropnoZF[v][k, 5, n, o] =
       {#[[1]], 1/teslaConv e^(-LB #[[1]]) #[[2]]} & /@ dataEVD150drop[v][k, 3, n, o];

      dataEVDFT[v][k, 1, n, o] =
       {#[[1]], e^(-i (interdatEVD[v][phOrder][#[[1]]])) #[[2]]} & /@ fftR[dataEVD[v][k, 5, n, o]] // Quiet;
      dataEVDFT[v][k, 2, n, o] = {#[[1]], e^(-i 0.) #[[2]]} & /@ fftR[dataEVD[v][k, 5, n, o]] // Quiet;
      dataEVDFT[v][k, 3, n, o] = {#[[1]], e^(-i (interdatEVD150drop[v][phOrder][#[[1]]])) #[[2]]} & /@
         fftR[dataEVD150drop[v][k, 5, n, o]] // Quiet;
      dataEVDFT[v][k, 4, n, o] =
       {#[[1]], e^(-i 0.) #[[2]]} & /@ fftR[dataEVD150drop[v][k, 5, n, o]] // Quiet;
      dataEVDFT[v][k, 5, n, o] = {#[[1]], e^(-i (interdatEVD150drop[v][phOrder][#[[1]]])) #[[2]]} & /@
         fftR[dataEVD150dropnoZF[v][k, 5, n, o]] // Quiet;
      dataEVDFT[v][k, 6, n, o] =
       {#[[1]], e^(-i 0.) #[[2]]} & /@ fftR[dataEVDnoZF[v][k, 5, n, o]] // Quiet;
      , {o, omax[[n]]}];
    , {n, nmax}, {v, vmax}];
  , "{n,o,v} = " <> ToString@{n, o, v} <> " of " <> ToString@omax[[n]] <> " scans"];
```

Expected compiling time = 0.0767 min

2/15/24 17:20:24 In[]:=

```
(*/////////////////////////*)
(*Export the data for future processing*)
Monitor[
  Table[
   Export[outfilesReviewersEVD[[v, n]],
    Table[dataEVDFT[v][k, m, n, o][[1 ;; np ;; q]], {o, omax[[n]]}, {m, mmaxEVD}, {k, 3, 3}],
    "ComplexKeys" → {"Re", "Im"}], {n, nmax}, {v, vmax}],
  "{n,o,v} = " <> ToString@{n, o, v} <> " of " <> ToString@omax[[n]] <> " scans"];
```

Check I have all the Fourier data

2/15/24 17:25:36 In[]:=

```
{Manipulate[ListPlot[{#[[1]], Re@#[[2]]} & /@ dataFT[k, m, n, o], PlotRange → range],
   {m, 1, mmax, 1}, {n, 1, nmax, 1}, {o, 1, omax[[n]], 1}],
  Manipulate[ListPlot[{#[[1]], Re@#[[2]]} & /@ dataEVDFT[v][k, m, n, o], PlotRange → range],
   {m, 1, mmaxEVD, 1}, {n, 1, nmax, 1}, {o, 1, omax[[n]], 1}, {v, 1, vmax, 1}]} // Row
```

2nd loop: import processed files and obtain *J*-couplings values

Preliminary

(*for some reason if there is only one value of k=1,2,3. Mathematica exports it as k=1*)

(*Only if I import dataFT[k,m,n,o] from a checkpoint*)

```
Clear[Jcoup, JcoupRatio, dataFT];
ClearR[dataFT]
Clear@aux;
Clear@q; q = 1;
Monitor[
  Table[
   aux[n] =
    Import[outfilesReviewers[[n]], {"Datasets", "Dataset1"}, "ComplexKeys" → {"Re", "Im"}];
   Table[
    dataFT[k, m, n, o] = {Re@#[[1]], #[[2]]} & /@ (aux[n][[o, m, k - 2]][[;; ;; q]]);
    , {o, omax[[n]]}, {m, mmax}, {k, 3, 3}];
   , {n, nmax}], "{m,n,o} = " <> ToString@{m, n, o} <> " of " <> ToString@omax[[n]] <> " scans"];
Clear@aux;
```

```
Clear@q; q = 1;
Monitor[
  Table[
   aux[n, v] = Import[outfilesReviewersEVD[[v, n]],
     {"Datasets", "Dataset1"}, "ComplexKeys" → {"Re", "Im"}];
   Table[
    dataFT[k, mmax + (v - 1) mmaxEVD + m, n, o] =
      {Re@#[[1]], #[[2]]} & /@ (aux[n, v][[o, m, k - 2]][[;; ;; q]]);
    , {o, omax[[n]]}, {m, mmaxEVD}, {k, 3, 3}];
   , {n, nmax}, {v, vmax}], "n = " <> ToString@n <> " of " <> ToString@omax[[n]] <> " scans"];
Clear@aux;
```

```
{Manipulate[ListPlot[{#[[1]], Re@#[[2]]} & /@ dataFT[k, m, n, o], PlotRange → range],
    {m, 1, mmaxTot, 1}, {n, 1, nmax, 1}, {o, 1, omax[[n]], 1}],
   Manipulate[ListPlot[{#[[1]], Re@#[[2]]} & /@ dataEVDFT[v][k, m, n, o], PlotRange → range],
    {m, 1, mmaxEVD, 1}, {n, 1, nmax, 1}, {o, 1, omax[[n]], 1}, {v, 1, 3, 1}]} // Row;
```

```
β = 0 °;
s = 1;
range = {{0, 200}, 4 {-1, 1}};
l = 1; p = 2;
rate = 1000;
tacq0 = 0.05; tacq = 2.048;
MA = 0.025;
ZF = tacq; LB = 0.;
k = 3; kmax = 3;
n = 5;
o = 1;
ν1 = 20; ν2 = 1000; order = 3;

Clear@functionPlot;
functionPlot[m_, n_] :=
  ListPlot[{
    {#[[1]], Re[ⅇ^(ⅈ β) #[[2]]]} & /@ dataFT[k, m, n, o][[;; ;; s]],
    {#[[1]], Im[ⅇ^(ⅈ β) #[[2]]]} & /@ dataFT[k, m, n, o][[;; ;; s]],
    {#[[1]], Abs[ⅇ^(ⅈ β) #[[2]]]} & /@ dataFT[k, m, n, o][[;; ;; s]]
   }
   ,
   PlotRange → range, PlotLabel → "dataFT[k," <> ToString@m <> "," <> ToString@n <> ",1]",
   FrameLabel → {"Frequency (Hz)", "Signal (pT/Hz)"},
   Frame → {True, True, False, False},
   PlotLegends → Placed[{"Real", "Imaginary", "Magnitude"}, {Right, Bottom}]
   (*,Placed[{"Real","Imaginary","Magnitude"},{Below,Left}]*)];
```

2/21/24 11:48:17 In[]:=

```
Manipulate[functionPlot[m, n] // Rasterize, {m, 1, mmaxTot, 1}, {n, 1, nmax, 1}]
```

2/21/24 11:50:07 In[]:=

```
Export[FileNameJoin[{datafolderMain, "Answer to Reviewers v2",
    "Fig7[k,m=" <> ToString@Range[mmaxTot] <> "," <> ToString[1] <> "] new plot Range.pdf"}],
  Table[functionPlot[m, 1], {m, mmaxTot}] // Column, ImageSize → Large];
```

2/21/24 11:50:22 In[]:=

```
Export[FileNameJoin[{datafolderMain, "Answer to Reviewers v2",
    "Fig7[k,m=" <> ToString@Range[mmaxTot] <> "," <> ToString[1] <> "] new plot Range.gif"}],
  Manipulate[functionPlot[m, 1], {m, 1, mmaxTot, 1}], "DisplayDurations" → 0.5];
```

(*dataFit[k,m,n,o,r] where r determines the frequency rang. r=1 is group A ranging 48-60 Hz. r=2 is group B ranging 90-120 Hz.r=3 ranges 151-190 Hz. I do all of them on the same cell*)

```
n = nmax
rangeGroup = {{51, 60}, {90, 120}, {151, 190}};
Clear[a, ν, A, f, b, ϕ, g, model];
gmax = 2; lines = 50 {1};

model[1] = Sum[a[i] E^(I ϕ[i]) ComplexLorentzian[ν, ν0[i], Γ[i]], {i, 2, 2}] + Sum[g[i] ν^i, {i, 0, gmax}];
parameters[k, 3, 1, 1] = {(*a[1],ϕ[1],(*{ν0[1],105},*)Γ[1],*){a[2], 0.8836},
    {ϕ[2], 0.29}, {ν0[2], 52.4021}, {Γ[2], 1.34}}~Join~(g /@ Range[0, gmax]);

model[2] = Sum[a[i] E^(I ϕ[i]) ComplexLorentzian[ν, ν0[i], Γ[i]], {i, 1, 1}] +
   Sum[a[i] E^(I ϕ[i]) ComplexLorentzian[ν, ν0[i], Γ[i]], {i, 2, 2}] + Sum[g[i] ν^i, {i, 0, gmax}];
parameters[k, 3, 1, 2] = {{a[1], 3.52}, {ϕ[1], -13.4}, {ν0[1], 104.7841}, {Γ[1], 1.17},
    {a[2], 3.03}, {ϕ[2], 0.67}, {ν0[2], 110.114}, {Γ[2], 0.98}}~Join~(g /@ Range[0, gmax]);

model[3] = Sum[a[i] E^(I ϕ[i]) ComplexLorentzian[ν, ν0[i], Γ[i]], {i, 1, 1}] +
   Sum[a[i] E^(I ϕ[i]) ComplexLorentzian[ν, ν0[i], Γ[i]], {i, 2, 2}] + Sum[g[i] ν^i, {i, 0, gmax}];
parameters[k, 3, 1, 3] = {{a[1], 1.35}, {ϕ[1], -0.11}, {ν0[1], 157.171}, {Γ[1], 0.97},
    {a[2], 1.55}, {ϕ[2], 0}, {ν0[2], 183.554}, {Γ[2], 0.98}}~Join~(g /@ Range[0, gmax]);
```

2/16/24 13:41:10 Out[]=

```
8
```

```
Monitor[
  Table[
dataFit[k, m, n, o, r] = Join[
      Select[dataFT[k, m, n, o], rangeGroup[[r, 1]] < #[[1]] < rangeGroup[[r, 2]] &]
     ];
fit[k, 3, m, n, o, r] = NonlinearModelFit[
       {#[[1]], Re[#[[2]]]} & /@ dataFit[k, m, n, o, r],
       Re@model[r],
       parameters[k, 3, 1, r], ν, MaxIterations → 1000] // Quiet;
    , {n, nmax}, {o, omax[[n]]}, {r, 1, 3}, {m, mmaxTot}];,
  "{m,n,o,r} = " <> ToString@{m, n, o, r} <> " of " <> ToString@omax[[n]] <> " scans"];
```

2/16/24 13:48:25 In[]:=

```
{fit[k, 3, 1, nmax, omax[[nmax]], 3]["ParameterTable"],
   fit[k, 3, 1, nmax, omax[[nmax]], 3]["ParameterTable"],
   fit[k, 3, 1, nmax, omax[[nmax]], 3]["ParameterTable"],
   fit[k, 3, 1, nmax, omax[[nmax]], 3]["ParameterTable"],
   fit[k, 3, 1, nmax, omax[[nmax]], 3]["ParameterTable"]
  } // Quiet // Row
```

2/16/24 13:48:26 Out[]=

	Estimate	Standard Error	t-Statistic	P-Value
a[1]	2.743	0.168338	16.2946	6.3878×10^{-35}
ϕ[1]	0.0811986	0.0633382	1.28198	0.201839
ν0[1]	157.147	0.0405257	3877.7	0.
Γ[1]	1.02432	0.0798067	12.8351	7.24395×10^{-26}
a[2]	3.08071	0.165707	18.5913	1.17291×10^{-40}
ϕ[2]	−0.165684	0.0567474	−2.91967	0.00404854
ν0[2]	183.599	0.036175	5075.3	0.
Γ[2]	1.02358	0.0704226	14.5349	2.30555×10^{-30}
g[0]	3.45661	2.87414	1.20266	0.231017
g[1]	−0.0424003	0.0338231	−1.25359	0.211954
g[2]	0.000127424	0.0000991336	1.28538	0.200653

	Estimate	Standard Error	t-Statistic	P-Value
a[1]	2.743	0.168338	16.2946	6.3878×10^{-35}
ϕ[1]	0.0811986	0.0633382	1.28198	0.201839
ν0[1]	157.147	0.0405257	3877.7	0.
Γ[1]	1.02432	0.0798067	12.8351	7.24395×10^{-26}
a[2]	3.08071	0.165707	18.5913	1.17291×10^{-40}
ϕ[2]	−0.165684	0.0567474	−2.91967	0.00404854
ν0[2]	183.599	0.036175	5075.3	0.
Γ[2]	1.02358	0.0704226	14.5349	2.30555×10^{-30}
g[0]	3.45661	2.87414	1.20266	0.231017
g[1]	−0.0424003	0.0338231	−1.25359	0.211954
g[2]	0.000127424	0.0000991336	1.28538	0.200653

	Estimate	Standard Error	t-Statistic	P-Value
a[1]	2.743	0.168338	16.2946	6.3878×10^{-35}
ϕ[1]	0.0811986	0.0633382	1.28198	0.201839
ν0[1]	157.147	0.0405257	3877.7	0.
Γ[1]	1.02432	0.0798067	12.8351	7.24395×10^{-26}
a[2]	3.08071	0.165707	18.5913	1.17291×10^{-40}
ϕ[2]	−0.165684	0.0567474	−2.91967	0.00404854
ν0[2]	183.599	0.036175	5075.3	0.
Γ[2]	1.02358	0.0704226	14.5349	2.30555×10^{-30}
g[0]	3.45661	2.87414	1.20266	0.231017
g[1]	−0.0424003	0.0338231	−1.25359	0.211954
g[2]	0.000127424	0.0000991336	1.28538	0.200653

	Estimate	Standard Error	t-Statistic	P-Value
a[1]	2.743	0.168338	16.2946	6.3878×10^{-35}
ϕ[1]	0.0811986	0.0633382	1.28198	0.201839
ν0[1]	157.147	0.0405257	3877.7	0.
Γ[1]	1.02432	0.0798067	12.8351	7.24395×10^{-26}
a[2]	3.08071	0.165707	18.5913	1.17291×10^{-40}
ϕ[2]	−0.165684	0.0567474	−2.91967	0.00404854
ν0[2]	183.599	0.036175	5075.3	0.
Γ[2]	1.02358	0.0704226	14.5349	2.30555×10^{-30}
g[0]	3.45661	2.87414	1.20266	0.231017
g[1]	−0.0424003	0.0338231	−1.25359	0.211954
g[2]	0.000127424	0.0000991336	1.28538	0.200653

	Estimate	Standard Error	t-Statistic	P-Value
a[1]	2.743	0.168338	16.2946	6.3878×10^{-35}
ϕ[1]	0.0811986	0.0633382	1.28198	0.201839
ν0[1]	157.147	0.0405257	3877.7	0.
Γ[1]	1.02432	0.0798067	12.8351	7.24395×10^{-26}
a[2]	3.08071	0.165707	18.5913	1.17291×10^{-40}
ϕ[2]	−0.165684	0.0567474	−2.91967	0.00404854
ν0[2]	183.599	0.036175	5075.3	0.
Γ[2]	1.02358	0.0704226	14.5349	2.30555×10^{-30}
g[0]	3.45661	2.87414	1.20266	0.231017
g[1]	−0.0424003	0.0338231	−1.25359	0.211954
g[2]	0.000127424	0.0000991336	1.28538	0.200653

```
np = -1; q = 1;
Clear[aux, aux2]
aux2 = {5, 9, 9} + gmax;
Print["Expected compiling time = " <> ToString[
   NumberForm[vmax * 4/(3 * 3 * 7 * Sum[omax[[n]], {n, 1, 2}]) (3 * mmaxTot * Sum[omax[[n]], {n, 1, nmax}]) / 60, 3] // N] <> " min"];
Monitor[
  Table[
    aux[r] = ReplacePart[
      ConstantArray[ConstantArray[0., {aux2[[r]], 4}], {omax[[nmax]], nmax}],
      Flatten@{Table[{o, n} → fit[k, 3, m, n, o, r]["ParameterTable"][[1, 1, 2 ;;]][[
            All, 2 ;;]] // Quiet, {n, nmax}, {o, omax[[n]]}]}
     ];
    Export[
     FileNameJoin[{datafolderMain, "Answer to Reviewers v2", "fits", "fitBaseline[2][" <>
        ToString@m <> "," <> ToString@r <> "].h5"}], aux[r], {"Datasets", "Dataset1"}];
    Clear@aux;, {r, 1, 3}, {m, mmaxTot}];
  , "{m,n,o,r} = " <> ToString@{m, n, o, r} <> " of " <> ToString@omax[[n]] <> " scans"];
Clear@aux2
```

Expected compiling time = 114. min

2/15/24 19:10:08 In[]:=

```
m = 1
r = 1
```

2/15/24 19:10:08 Out[]=

```
1
```

2/15/24 19:10:08 Out[]=

```
1
```

2/16/24 15:03:22 In[]:=

```
fit[k, 3, 1, nmax, omax[[nmax]], 3]["ParameterTable"][[1, 1, 2 ;;]][[All, 2 ;;]] // Quiet //
 MatrixForm
```

2/16/24 15:03:22 Out[]//MatrixForm=

2.743	0.168338	16.2946	6.3878×10^{-35}
0.0811986	0.0633382	1.28198	0.201839
157.147	0.0405257	3877.7	0.
1.02432	0.0798067	12.8351	7.24395×10^{-26}
3.08071	0.165707	18.5913	1.17291×10^{-40}
-0.165684	0.0567474	-2.91967	0.00404854
183.599	0.036175	5075.3	0.
1.02358	0.0704226	14.5349	2.30555×10^{-30}
3.45661	2.87414	1.20266	0.231017
-0.0424003	0.0338231	-1.25359	0.211954
0.000127424	0.0000991336	1.28538	0.200653

```
Table[{o, n} → fit[k, 3, 1, n, o, 1]["ParameterTable"][[1, 1, 2 ;;]][[All, 2 ;;]] // Quiet,
 {n, nmax}, {o, omax⟦n⟧}]
```

2/15/24 19:02:13 In[]:=

```
Manipulate[fit[k, 3, 1, 8, o, 3]["BestFitParameters"], {o, 1, 360, 1}]
```

""	"Estimate"	"Standard Error"	"t-Statistic"	"P-Value"
a[2]	0.883932	0.0252562	34.9985	4.41408×10^{-27}
ϕ[2]	0.290547	0.0282952	10.2684	1.18118×10^{-11}
ν0[2]	52.3926	0.0212779	2462.3	5.07502×10^{-86}
Γ[2]	1.3466	0.0394078	34.1709	9.31111×10^{-27}
g[0]	-0.0155997	0.00304157	-5.12883	0.0000136622

""	"Estimate"	"Standard Error"	"t-Statistic"	"P-Value"
a[1]	3.52489	0.0494985	71.2121	2.39552×10^{-96}
ϕ[1]	-13.4033	0.013451	-996.453	1.94629×10^{-226}
ν0[1]	104.784	0.0103384	10135.4	$2.819939732050266\times10^{-341}$
Γ[1]	1.17132	0.0211218	55.4553	2.59013×10^{-84}
a[2]	3.03362	0.044571	68.0627	3.6968×10^{-94}
ϕ[2]	0.673194	0.0144345	46.6377	4.23002×10^{-76}
ν0[2]	110.115	0.00932849	11804.2	$8.019215114261086\times10^{-349}$
Γ[2]	0.989302	0.0188814	52.3956	1.31227×10^{-81}
g[0]	-0.0214491	0.00348722	-6.15077	1.17361×10^{-8}

""	"Estimate"	"Standard Error"	"t-Statistic"	"P-Value"
a[1]	1.34866	0.017153	78.6254	2.03431×10^{-124}
ϕ[1]	-0.118191	0.0130153	-9.0809	5.55901×10^{-16}
ν0[1]	157.171	0.00849985	18491.	$1.031116110735593\times10^{-481}$
Γ[1]	0.967969	0.0166771	58.0419	3.7258×10^{-105}
a[2]	1.55478	0.0174658	89.0183	2.17891×10^{-132}
ϕ[2]	-0.00407703	0.0112267	-0.363154	0.716998
ν0[2]	183.555	0.00747885	24543.2	$2.786097520249636\times10^{-500}$
Γ[2]	0.98106	0.0148417	66.1018	2.32043×10^{-113}
g[0]	0.000440871	0.00126761	0.347797	0.728477

```
o = 1;
col = {Orange, Red, Yellow}; s = 1;
Clear@Fig1;
Monitor[
  Table[Fig1[k, m, o] = Table[
      Show[{
        ListPlot[{
          {#[[1]], Re[e^(i β) #[[2]]]} & /@ dataFT[k, m, n, o][[;; ;; s]]
         }, PlotRange → {{0, 200}, 4 {-0.5, 1}},
         FrameLabel → {"Frequency (Hz)", "Signal (pT/Hz)"}],
        Table[Plot[
          fit[k, 3, m, n, o, r][ν]
          , {ν, rangeGroup[[r, 1]], rangeGroup[[r, 2]]}, PlotStyle → {col[[r]], Opacity@0.3}
         ], {r, 1, 3}]}]
      , {n, nmax}], {m, mmaxTot}];,
  "{m,n,o} = " <> ToString@{m, n, o} <> " of " <> ToString@omax[[n]] <> " scans"];
```

2/15/24 17:22:23 In[]:=

```
np = -1; q = 1;
Table[
  Export[FileNameJoin[
     {datafolderMain, "Answer to Reviewers v2", "Fig1[k," <> ToString@m <> ",1].pdf"}],
    Fig1[k, m, 1][[1 ;; nmax ;; q]] // GraphicsColumn] // Quiet;
  (*Export[FileNameJoin[
     {datafolderMain,"Answer to Reviewers v2","Fig1[k,"<>ToString@m<>",1][1].pdf"}],
    ArrayReshape[Fig1[k,m,1][[1;;4;;q]],{2,2}]//GraphicsGrid]//Quiet;
  Export[FileNameJoin[
     {datafolderMain,"Answer to Reviewers v2","Fig1[k,"<>ToString@m<>",1][2].pdf"}],
    ArrayReshape[Fig1[k,m,1][[5;;nmax;;q]],{2,2}]//GraphicsGrid]//Quiet;*)
  (*Export[FileNameJoin[
     {datafolderMain,"Answer to Reviewers v2","Fig1[k,"<>ToString@m<>",1][3].pdf"}],
    ArrayReshape[Fig1[k,m,1][[9;;12;;q]],{2,2}]//GraphicsGrid]//Quiet;
  Export[FileNameJoin[
     {datafolderMain,"Answer to Reviewers v2","Fig1[k,"<>ToString@m<>",1][4].pdf"}],
    ArrayReshape[Fig1[k,m,1][[13;;15;;q]],{2,2}]//GraphicsGrid]//Quiet;*)
  , {m, mmaxTot}];
```

2/15/24 17:46:51 In[]:=

```
Export[FileNameJoin[
   {datafolderMain, "Answer to Reviewers v2", "Fig1[k," <> ToString@m <> ",1].gif"}],
  Manipulate[Fig1[k, m, 1][[1]], {m, 1, mmaxTot, 1}], "DisplayDurations" → 0.5];
```

Import the fitting parameters

2/16/24 15:04:37 In[]:=

```
Clear[aux, fit, q];
q = 1;
Monitor[Table[
   Table[aux[r] = Import[FileNameJoin[{datafolderMain, "Answer to Reviewers v2",
        "fits", "fit[" <> ToString@m <> "," <> ToString@r <> "].h5"}],
      {"Datasets", "Dataset1"}, "ComplexKeys" → {"Re", "Im"}], {r, 1, 3}];
   Table[fit[k, 3, m, n, o, r] = {#⟦1⟧, #⟦2⟧} & /@ (aux[r]⟦o, n⟧⟦ ;;  ;; q⟧),
    {n, nmax}, {o, omax⟦n⟧}, {r, 1, 3}];
   Clear@aux;, {m, mmaxTot}], "m = " <> ToString@m <> " of " <> ToString@mmaxTot <> " scans"];
```

2/16/24 15:07:15 In[]:=

```
Clear[aux, q];
q = 1;
Monitor[Table[
   Table[aux[r] = Import[FileNameJoin[{datafolderMain, "Answer to Reviewers v2",
        "fits", "fitBaseline[1][" <> ToString@m <> "," <> ToString@r <> "].h5"}],
      {"Datasets", "Dataset1"}, "ComplexKeys" → {"Re", "Im"}], {r, 1, 3}];
   Table[fitBaseline[1][k, 3, m, n, o, r] = {#⟦1⟧, #⟦2⟧} & /@ (aux[r]⟦o, n⟧⟦ ;;  ;; q⟧),
    {n, nmax}, {o, omax⟦n⟧}, {r, 1, 3}];
   Clear@aux;, {m, mmaxTot}], "m = " <> ToString@m <> " of " <> ToString@mmaxTot <> " scans"];
```

2/16/24 15:07:30 In[]:=

```
Clear[aux, q];
q = 1;
Monitor[Table[
   Table[aux[r] = Import[FileNameJoin[{datafolderMain, "Answer to Reviewers v2",
        "fits", "fitBaseline[2][" <> ToString@m <> "," <> ToString@r <> "].h5"}],
      {"Datasets", "Dataset1"}, "ComplexKeys" → {"Re", "Im"}], {r, 1, 3}];
   Table[fitBaseline[2][k, 3, m, n, o, r] = {#⟦1⟧, #⟦2⟧} & /@ (aux[r]⟦o, n⟧⟦ ;;  ;; q⟧),
    {n, nmax}, {o, omax⟦n⟧}, {r, 1, 3}];
   Clear@aux;, {m, mmaxTot}], "m = " <> ToString@m <> " of " <> ToString@mmaxTot <> " scans"];
```

2/16/24 15:07:45 In[]:=

```
Manipulate[{fit[k, 3, m, n, o, r] // MatrixForm, fitBaseline[1][k, 3, m, n, o, r] // MatrixForm,
   fitBaseline[2][k, 3, m, n, o, r] // MatrixForm} // Row,
 {m, 1, mmaxTot, 1}, {n, 1, nmax, 1}, {o, 1, omax⟦n⟧, 1}, {{r, 3}, 1, 3, 1}]
```

2/16/24 15:10:08 In[]:=

```
Clear@aux
fitMan = Manipulate[
  aux[n] = {
    {Mean[fit[k, 3, #, n, 1, 1]⟦3, 1⟧ & /@ Range[mmaxTot]],
     STD[fit[k, 3, #, n, 1, 1]⟦3, 1⟧ & /@ Range[mmaxTot]]},
    {Mean[fit[k, 3, #, n, 1, 2]⟦3, 1⟧ & /@ Range[mmaxTot]],
     STD[fit[k, 3, #, n, 1, 2]⟦3, 1⟧ & /@ Range[mmaxTot]]},
    {Mean[fit[k, 3, #, n, 1, 2]⟦7, 1⟧ & /@ Range[mmaxTot]],
     STD[fit[k, 3, #, n, 1, 2]⟦7, 1⟧ & /@ Range[mmaxTot]]},
```

```
    {Mean[fit[k, 3, #, n, 1, 3][[3, 1]] & /@ Range[mmaxTot]],
     STD[fit[k, 3, #, n, 1, 3][[3, 1]] & /@ Range[mmaxTot]]},
    {Mean[fit[k, 3, #, n, 1, 3][[7, 1]] & /@ Range[mmaxTot]],
     STD[fit[k, 3, #, n, 1, 3][[7, 1]] & /@ Range[mmaxTot]]}
  };
 auxBaseline[1][n] = {
    {Mean[fitBaseline[1][k, 3, #, n, 1, 1][[3, 1]] & /@ Range[mmaxTot]],
     STD[fitBaseline[1][k, 3, #, n, 1, 1][[3, 1]] & /@ Range[mmaxTot]]},
    {Mean[fitBaseline[1][k, 3, #, n, 1, 2][[3, 1]] & /@ Range[mmaxTot]],
     STD[fitBaseline[1][k, 3, #, n, 1, 2][[3, 1]] & /@ Range[mmaxTot]]},
    {Mean[fitBaseline[1][k, 3, #, n, 1, 2][[7, 1]] & /@ Range[mmaxTot]],
     STD[fitBaseline[1][k, 3, #, n, 1, 2][[7, 1]] & /@ Range[mmaxTot]]},
    {Mean[fitBaseline[1][k, 3, #, n, 1, 3][[3, 1]] & /@ Range[mmaxTot]],
     STD[fitBaseline[1][k, 3, #, n, 1, 3][[3, 1]] & /@ Range[mmaxTot]]},
    {Mean[fitBaseline[1][k, 3, #, n, 1, 3][[7, 1]] & /@ Range[mmaxTot]],
     STD[fitBaseline[1][k, 3, #, n, 1, 3][[7, 1]] & /@ Range[mmaxTot]]}
  };
 auxBaseline[2][n] = {
    {Mean[fitBaseline[2][k, 3, #, n, 1, 1][[3, 1]] & /@ Range[mmaxTot]],
     STD[fitBaseline[2][k, 3, #, n, 1, 1][[3, 1]] & /@ Range[mmaxTot]]},
    {Mean[fitBaseline[2][k, 3, #, n, 1, 2][[3, 1]] & /@ Range[mmaxTot]],
     STD[fitBaseline[2][k, 3, #, n, 1, 2][[3, 1]] & /@ Range[mmaxTot]]},
    {Mean[fitBaseline[2][k, 3, #, n, 1, 2][[7, 1]] & /@ Range[mmaxTot]],
     STD[fitBaseline[2][k, 3, #, n, 1, 2][[7, 1]] & /@ Range[mmaxTot]]},
    {Mean[fitBaseline[2][k, 3, #, n, 1, 3][[3, 1]] & /@ Range[mmaxTot]],
     STD[fitBaseline[2][k, 3, #, n, 1, 3][[3, 1]] & /@ Range[mmaxTot]]},
    {Mean[fitBaseline[2][k, 3, #, n, 1, 3][[7, 1]] & /@ Range[mmaxTot]],
     STD[fitBaseline[2][k, 3, #, n, 1, 3][[7, 1]] & /@ Range[mmaxTot]]}
  };
 {ListPlot[
     MapThread[{#1, Around[#2, #3]} &,
      {
       {1, 2, 3, 4, 5},
       #[[1]] & /@ aux[n],
       1000 #[[2]] & /@ aux[n]
      }]
     , Joined → False,
     PlotMarkers → {Automatic, Medium},
     PlotLabel → "w/o baseline in fitting model. Error bars × 1000",
     Axes → True,
     FrameLabel → {None, "Frequency (Hz)"},
     ImagePadding → All,
     Frame → True,
     PlotRange → {All, {-50, 250}}
     (*AspectRatio→aspRat,*)
    ],
    ListPlot[
     MapThread[{#1, Around[#2, #3]} &,
      {
       {1, 2, 3, 4, 5},
```

```
      #⟦1⟧ & /@ auxBaseline[1][n],
      1000 #⟦2⟧ & /@ auxBaseline[1][n]
     }]
    , Joined → False,
    PlotMarkers → {Automatic, Medium},
    PlotLabel → "w/ 1st degree baseline in fitting model. Error bars × 1000",
    Axes → True,
    FrameLabel → {None, "Frequency (Hz)"},
    ImagePadding → All,
    Frame → True,
    PlotRange → {All, {-50, 250}}
    (*AspectRatio→aspRat,*)
   ],
   ListPlot[
    MapThread[{#1, Around[#2, #3]} &,
     {
      {1, 2, 3, 4, 5},
      #⟦1⟧ & /@ auxBaseline[2][n],
      1000 #⟦2⟧ & /@ auxBaseline[2][n]
     }]
    , Joined → False,
    PlotMarkers → {Automatic, Medium},
    PlotLabel → "w/ 2nd degree baseline in fitting model. Error bars × 1000",
    Axes → True,
    FrameLabel → {None, "Frequency (Hz)"},
    ImagePadding → All,
    Frame → True,
    PlotRange → {All, {-50, 250}}
    (*AspectRatio→aspRat,*)
   ]
  } // Row, {n, 1, nmax, 1}]
```

Export *J*-couplings and *J*-couplings ratios

2/10/24 20:05:37 In[]:=

```
n = nmax
```

2/10/24 20:05:37 Out[]=

```
8
```

2/10/24 20:05:37 In[]:=

```
Clear[Jcoup, JcoupRatio, aux];
Monitor[
  Table[
    (*aux[k,3,m,n,o,r]=
     Thread[{fit[k,3,m,n,o,r]["BestFitParameters"]⟦All,2⟧,fit[k,3,m,n,o,r][
         "ParameterErrors"]}]//Quiet(*in case I already have the variable*)*)
    aux[k, 3, m, n, o, r] = fit[k, 3, m, n, o, r](*in case I import the data*)
    , {m, mmaxTot}, {n, nmax}, {o, omax⟦n⟧}, {r, 1, 3}];,
  "{m,n,o,r} = " <> ToString@{m, n, o, r} <> " of " <> ToString@omax⟦n⟧ <> " scans"];
```

2/10/24 20:05:38 In[]:=

```
Monitor[
  Table[
    Jcoup[k, m, n, o] = {
      aux[k, 3, m, n, o, 1][[3]],
      aux[k, 3, m, n, o, 2][[3]],
      aux[k, 3, m, n, o, 2][[7]],
      aux[k, 3, m, n, o, 3][[3]],
      aux[k, 3, m, n, o, 3][[7]]
     };

JcoupRatio[k, m, n, o] = {
       ((2/3) Jcoup[k, m, n, o][[3, 1]])/(Jcoup[k, m, n, o][[1, 1]])
        {1, Sqrt[((Jcoup[k, m, n, o][[3, 2]])/(Jcoup[k, m, n, o][[3, 1]]))^2 + ((Jcoup[k, m, n, o][[1, 2]])/(Jcoup[k, m, n, o][[1, 1]]))^2]},
       ((2/3) Jcoup[k, m, n, o][[3, 1]])/((1/2) Jcoup[k, m, n, o][[2, 1]])
        {1, Sqrt[((Jcoup[k, m, n, o][[3, 2]])/(Jcoup[k, m, n, o][[3, 1]]))^2 + ((Jcoup[k, m, n, o][[2, 2]])/(Jcoup[k, m, n, o][[2, 1]]))^2]},
       ((2/3) Jcoup[k, m, n, o][[3, 1]])/((1/3) Jcoup[k, m, n, o][[4, 1]])
        {1, Sqrt[((Jcoup[k, m, n, o][[3, 2]])/(Jcoup[k, m, n, o][[3, 1]]))^2 + ((Jcoup[k, m, n, o][[4, 2]])/(Jcoup[k, m, n, o][[4, 1]]))^2]},
       ((2/5) Jcoup[k, m, n, o][[5, 1]])/(Jcoup[k, m, n, o][[1, 1]])
        {1, Sqrt[((Jcoup[k, m, n, o][[5, 2]])/(Jcoup[k, m, n, o][[5, 1]]))^2 + ((Jcoup[k, m, n, o][[1, 2]])/(Jcoup[k, m, n, o][[1, 1]]))^2]},
       ((2/5) Jcoup[k, m, n, o][[5, 1]])/((1/2) Jcoup[k, m, n, o][[2, 1]])
        {1, Sqrt[((Jcoup[k, m, n, o][[5, 2]])/(Jcoup[k, m, n, o][[5, 1]]))^2 + ((Jcoup[k, m, n, o][[2, 2]])/(Jcoup[k, m, n, o][[2, 1]]))^2]},
       ((2/5) Jcoup[k, m, n, o][[5, 1]])/((1/3) Jcoup[k, m, n, o][[4, 1]])
        {1, Sqrt[((Jcoup[k, m, n, o][[5, 2]])/(Jcoup[k, m, n, o][[5, 1]]))^2 + ((Jcoup[k, m, n, o][[4, 2]])/(Jcoup[k, m, n, o][[4, 1]]))^2]}
     };
    , {m, mmaxTot}, {n, nmax}, {o, omax[[n]]}];
  , "{n,o} = " <> ToString@{n, o} <> " of " <> ToString@omax[[n]] <> " scans"];
Jcoup[k, 5, n, o] // MatrixForm
JcoupRatio[k, 5, n, o] // MatrixForm
```

2/10/24 20:05:39 Out[]//MatrixForm=

52.5013	0.0557209
104.74	0.0198183
110.072	0.021677
157.15	0.0443495
183.557	0.0332822

2/10/24 20:05:39 Out[]//MatrixForm=

1.39771	0.00150874
1.40122	0.000382677
1.40085	0.000482077
1.39849	0.00150576
1.40201	0.000367418
1.40164	0.000470165

2/10/24 20:05:39 In[]:=

```
Manipulate[ListPlot[
  {
   MapThread[{#1, Around[#2, #3]} &,
     {
      {1, 1, 2, 3, 3, 4},
      #[[1]] & /@ JcoupRatio[k, m, n, o],
      #[[2]] & /@ JcoupRatio[k, m, n, o]
     }
    ][[{2, 3, 5, 6}]]
  }
  , Joined → False,
  PlotMarkers → {Automatic, Medium},
  FrameLabel → {None, "J couplings ratio"},
  PlotLabel → "{m,n,o} = " <> ToString[{m, n, o}],
  FrameTicks → {{All, None},
    {{(*{1,"(2/3(3/2 × 1J15NH))/(1J14NH)"},*){1, "(2/3 (3/2 × 1J15NH))/(1/2 (2×1J14NH))"}, {2, "(2/3 (3/2 × 1J15NH))/(1/3 (3×1J14NH))"}(*,
      {4,"(2/5(5/2 × 1J15NH))/(1J14NH)"}*), {3, "(2/5 (5/2 × 1J15NH))/(1/2 (2×1J14NH))"}, {4, "(2/5 (5/2 × 1J15NH))/(1/3 (3×1J14NH))"}}, None}},
  Axes → True,
  ImagePadding → All,
  Frame → True,
  (*AspectRatio→aspRat,*)
  PlotRange → {All, {1.40, 1.402}},
  ImageSize → Large], {n, 1, nmax, 1}, {m, 1, mmaxTot, 1}, {o, 1, omax[[n]], 1}]
```

2/10/24 20:05:39 In[]:=

```
np = -1; q = 1;
jcoupnum = 5;
```

2/10/24 20:05:39 In[]:=

```
Table[
  Clear@aux;
  aux[1] = ReplacePart[ConstantArray[ConstantArray[0., {jcoupnum, 2}], {omax⟦nmax⟧, nmax}],
    Flatten@{Table[{o, n} → Jcoup[k, m, n, o]⟦1 ;; np ;; q⟧, {n, nmax}, {o, omax⟦n⟧}]}];
  aux[2] = ReplacePart[ConstantArray[ConstantArray[0., {jcoupnum + 1, 2}], {omax⟦nmax⟧, nmax}],
    Flatten@{Table[{o, n} → JcoupRatio[k, m, n, o]⟦1 ;; np ;; q⟧, {n, nmax}, {o, omax⟦n⟧}]}];
  Export[FileNameJoin[{datafolderMain, "Answer to Reviewers v2",
     "Jcoup[k," <> ToString@m <> ",n,o].h5"}], aux[1], {"Datasets", "Dataset1"}];
  Export[FileNameJoin[{datafolderMain, "Answer to Reviewers v2",
     "JcoupRatio[k," <> ToString@m <> ",n,o].h5"}], aux[2], {"Datasets", "Dataset1"}];
  Clear@aux;
  , {m, mmaxTot}];
```

Import *J*-couplings and finalize analysis

Preliminary

2/22/24 14:10:29 In[]:=

```
Clear[data];
datafolderMain = "T:\\Projects\\ZULF NMR\\Roman\\ZULF\\Papers\\Quadrupole
    project\\Phase IV Data\\Experiment 1 - Thermal spectra\\Partitions";
l = 1;
p = 2;
```

Remove 0.05 s initial data ⇒ 5000*0.05=250 points. m stores is all the aux variables I need data[i,m]. Take every l element. s is less points for the plots. 5000*1.024=5120 points less for 3.072 s

p is channel. data[k,m,n,o] n is for folders and o for scans

2/22/24 14:10:29 In[]:=

```
β = 0 °;
s = 1;
range = {{0, 200}, 1 {-1, 2}};
rate = 1000;
tacq0 = 0.05; tacq = 2.048;
MA = 0.025;
ZF = tacq; LB = 0.;
k = 3; kmax = 3;
n = 1;
o = 1;
ν1 = 20;
ν2 = 1000;
order = 3;
```

2/22/24 14:10:29 In[]:=

```
datafolder = {
   FileNameJoin[{datafolderMain, "00001 partitions of 36000 scans"}],
   FileNameJoin[{datafolderMain, "00002 partitions of 18000 scans"}],
   FileNameJoin[{datafolderMain, "00003 partitions of 12000 scans"}],
```

```
    FileNameJoin[{datafolderMain, "00004 partitions of 09000 scans"}],
    FileNameJoin[{datafolderMain, "00012 partitions of 03000 scans"}],
    FileNameJoin[{datafolderMain, "00036 partitions of 01000 scans"}],
    FileNameJoin[{datafolderMain, "00060 partitions of 00600 scans"}],
    FileNameJoin[{datafolderMain, "00360 partitions of 00100 scans"}],
    FileNameJoin[{datafolderMain, "01000 partitions of 00036 scans"}],
    FileNameJoin[{datafolderMain, "02250 partitions of 00016 scans"}],
    FileNameJoin[{datafolderMain, "03600 partitions of 00010 scans"}],
    FileNameJoin[{datafolderMain, "04500 partitions of 00008 scans"}],
    FileNameJoin[{datafolderMain, "09000 partitions of 00004 scans"}],
    FileNameJoin[{datafolderMain, "18000 partitions of 00002 scans"}],
    FileNameJoin[{datafolderMain, "36000 partitions of 00001 scans"}]
   };
outfiles = {
    FileNameJoin[{datafolderMain, "00001 partitions of 36000 scans loop.h5"}],
    FileNameJoin[{datafolderMain, "00002 partitions of 18000 scans loop.h5"}],
    FileNameJoin[{datafolderMain, "00003 partitions of 12000 scans loop.h5"}],
    FileNameJoin[{datafolderMain, "00004 partitions of 09000 scans loop.h5"}],
    FileNameJoin[{datafolderMain, "00012 partitions of 03000 scans loop.h5"}],
    FileNameJoin[{datafolderMain, "00036 partitions of 01000 scans loop.h5"}],
    FileNameJoin[{datafolderMain, "00060 partitions of 00600 scans loop.h5"}],
    FileNameJoin[{datafolderMain, "00360 partitions of 00100 scans loop.h5"}],
    FileNameJoin[{datafolderMain, "01000 partitions of 00036 scans loop.h5"}],
    FileNameJoin[{datafolderMain, "02250 partitions of 00016 scans loop.h5"}],
    FileNameJoin[{datafolderMain, "03600 partitions of 00010 scans loop.h5"}],
    FileNameJoin[{datafolderMain, "04500 partitions of 00008 scans loop.h5"}],
    FileNameJoin[{datafolderMain, "09000 partitions of 00004 scans loop.h5"}],
    FileNameJoin[{datafolderMain, "18000 partitions of 00002 scans loop.h5"}],
    FileNameJoin[{datafolderMain, "36000 partitions of 00001 scans loop.h5"}]
   };
outfilesReviewers = {
    FileNameJoin[
     {datafolderMain, "Answer to Reviewers v2", "00001 partitions of 36000 scans loop.h5"}],
    FileNameJoin[
     {datafolderMain, "Answer to Reviewers v2", "00002 partitions of 18000 scans loop.h5"}],
    FileNameJoin[
     {datafolderMain, "Answer to Reviewers v2", "00003 partitions of 12000 scans loop.h5"}],
    FileNameJoin[
     {datafolderMain, "Answer to Reviewers v2", "00004 partitions of 09000 scans loop.h5"}],
    FileNameJoin[
     {datafolderMain, "Answer to Reviewers v2", "00012 partitions of 03000 scans loop.h5"}],
    FileNameJoin[
     {datafolderMain, "Answer to Reviewers v2", "00036 partitions of 01000 scans loop.h5"}],
    FileNameJoin[
     {datafolderMain, "Answer to Reviewers v2", "00060 partitions of 00600 scans loop.h5"}],
    FileNameJoin[
     {datafolderMain, "Answer to Reviewers v2", "00360 partitions of 00100 scans loop.h5"}],
    FileNameJoin[
     {datafolderMain, "Answer to Reviewers v2", "01000 partitions of 00036 scans loop.h5"}],
    FileNameJoin[
     {datafolderMain, "Answer to Reviewers v2", "02250 partitions of 00016 scans loop.h5"}],
```

```
    FileNameJoin[
     {datafolderMain, "Answer to Reviewers v2", "03600 partitions of 00010 scans loop.h5"}],
    FileNameJoin[
     {datafolderMain, "Answer to Reviewers v2", "04500 partitions of 00008 scans loop.h5"}],
    FileNameJoin[
     {datafolderMain, "Answer to Reviewers v2", "09000 partitions of 00004 scans loop.h5"}],
    FileNameJoin[
     {datafolderMain, "Answer to Reviewers v2", "18000 partitions of 00002 scans loop.h5"}],
    FileNameJoin[
     {datafolderMain, "Answer to Reviewers v2", "36000 partitions of 00001 scans loop.h5"}]
   };

outfilesReviewersEVD = Table[{
     FileNameJoin[{datafolderMain, "Answer to Reviewers v2EVD [" <> ToString@v <> "]",
       "00001 partitions of 36000 scans loop.h5"}],
     FileNameJoin[{datafolderMain, "Answer to Reviewers v2EVD [" <> ToString@v <> "]",
       "00002 partitions of 18000 scans loop.h5"}],
     FileNameJoin[{datafolderMain, "Answer to Reviewers v2EVD [" <> ToString@v <> "]",
       "00003 partitions of 12000 scans loop.h5"}],
     FileNameJoin[{datafolderMain, "Answer to Reviewers v2EVD [" <> ToString@v <> "]",
       "00004 partitions of 09000 scans loop.h5"}],
     FileNameJoin[{datafolderMain, "Answer to Reviewers v2EVD [" <> ToString@v <> "]",
       "00012 partitions of 03000 scans loop.h5"}],
     FileNameJoin[{datafolderMain, "Answer to Reviewers v2EVD [" <> ToString@v <> "]",
       "00036 partitions of 01000 scans loop.h5"}],
     FileNameJoin[{datafolderMain, "Answer to Reviewers v2EVD [" <> ToString@v <> "]",
       "00060 partitions of 00600 scans loop.h5"}],
     FileNameJoin[{datafolderMain, "Answer to Reviewers v2EVD [" <> ToString@v <> "]",
       "00360 partitions of 00100 scans loop.h5"}],
     FileNameJoin[{datafolderMain, "Answer to Reviewers v2EVD [" <> ToString@v <> "]",
       "01000 partitions of 00036 scans loop.h5"}],
     FileNameJoin[{datafolderMain, "Answer to Reviewers v2EVD [" <> ToString@v <> "]",
       "02250 partitions of 00016 scans loop.h5"}],
     FileNameJoin[{datafolderMain, "Answer to Reviewers v2EVD [" <> ToString@v <> "]",
       "03600 partitions of 00010 scans loop.h5"}],
     FileNameJoin[{datafolderMain, "Answer to Reviewers v2EVD [" <> ToString@v <> "]",
       "04500 partitions of 00008 scans loop.h5"}],
     FileNameJoin[{datafolderMain, "Answer to Reviewers v2EVD [" <> ToString@v <> "]",
       "09000 partitions of 00004 scans loop.h5"}],
     FileNameJoin[{datafolderMain, "Answer to Reviewers v2EVD [" <> ToString@v <> "]",
       "18000 partitions of 00002 scans loop.h5"}],
     FileNameJoin[{datafolderMain, "Answer to Reviewers v2EVD [" <> ToString@v <> "]",
       "36000 partitions of 00001 scans loop.h5"}]
    }, {v, 3}];
nmax = 8; mmax = 7; mmaxEVD = 6; vmax = 3; mmaxTot = mmax + vmax * mmaxEVD;
omax = {1, 2, 3, 4, 12, 36, 60, 360, 1000, 2250, 3600, 4500, 9000, 18 000, 36 000};
```

Import *J*-couplings and *J*-couplings ratios

2/22/24 14:10:32 In[]:=

```
mSelection = {1, 2, 5, 6, 7} ~ Join ~ Range[8, 13]
mSelection // Length
mmaxTot = mSelection // Length
```

2/22/24 14:10:32 Out[]=

```
{1, 2, 5, 6, 7, 8, 9, 10, 11, 12, 13}
```

2/22/24 14:10:32 Out[]=

```
11
```

2/22/24 14:10:32 Out[]=

```
11
```

2/22/24 14:10:32 In[]:=

```
Clear@aux; nmax
Clear[Jcoup, JcoupRatio];
Clear@q; q = 1;
Print["Expected compiling time = " <> ToString[
    NumberForm[5/(11 Sum[omax[[n]], {n, 1, 1}]) ((mSelection // Length) * Sum[omax[[n]], {n, 1, nmax}]) / 60, 3] // N] <> " min"];
Monitor[
  Table[
    aux[1] = Import[FileNameJoin[{datafolderMain,
        "Answer to Reviewers v2", "Jcoup[k," <> ToString[mSelection[[m]]] <> ",n,o].h5"}],
      {"Datasets", "Dataset1"}, "ComplexKeys" → {"Re", "Im"}];
    aux[2] = Import[FileNameJoin[{datafolderMain, "Answer to Reviewers v2",
        "JcoupRatio[k," <> ToString[mSelection[[m]]] <> ",n,o].h5"}],
      {"Datasets", "Dataset1"}, "ComplexKeys" → {"Re", "Im"}];
    Jcoup[k, m, n, o] = {#[[1]], #[[2]]} & /@ (aux[1][[o, n]][[;; ;; q]]);
    JcoupRatio[k, m, n, o] = {#[[1]], #[[2]]} & /@ (aux[2][[o, n]][[;; ;; q]]);
    Clear@aux;
    , {m, mSelection // Length}, {n, nmax}, {o, omax[[n]]}];
  , "{m,n,o} = " <> ToString@{m, n, o} <> " of " <> ToString@omax[[n]] <> " scans"];
```

2/22/24 14:10:32 Out[]=

```
8
```

Expected compiling time = 39.8 min

2/22/24 14:41:01 In[]:=

```
aux[m_, n_, o_] := Show[{ListPlot[
      {
       MapThread[{#1, Around[#2, #3]} &,
          {
           {1, 1, 2, 3, 3, 4},
           #[[1]] & /@ JcoupRatio[k, m, n, o],
           #[[2]] & /@ JcoupRatio[k, m, n, o]
          }
         ][[{2, 3, 5, 6}]]
      }
      , Joined → False,
      PlotMarkers → {Automatic, Medium},
      FrameLabel → {None, "J couplings ratio"},
      PlotLabel → "{m,n,o} = " <> ToString[{m, n, o}],
      FrameTicks → {{All, None},
        {{(*{1,"(2/3(3/2 × 1J15NH))/(1J14NH)"},*){1, "(2/3 (3/2 × 1 J15NH))/(1/2 (2×1J14NH))"}, {2, "(2/3 (3/2 × 1 J15NH))/(1/3 (3×1J14NH))"}(*,{4,
           "(2/5(5/2 × 1J15NH))/(1J14NH)"}*), {3, "(2/5 (5/2 × 1 J15NH))/(1/2 (2×1J14NH))"}, {4, "(2/5 (5/2 × 1 J15NH))/(1/3 (3×1J14NH))"}}, None}},
      Axes → True,
      ImagePadding → All,
      Frame → True,
      (*AspectRatio→aspRat,*)
      PlotRange → {All, {1.40, 1.403}},
      ImageSize → Large]},
    Graphics[{Dashed, Blue, Line[{{1, Abs[-27.126 / 19.338]}, {6, Abs[-27.126 / 19.338]}}]}],
    Graphics[
     Text[Style["γ15 N/γ14 N", FontSize → 14, Blue], {2, 0.0001 + Abs[-27.126 / 19.338]}]]];

Manipulate[aux[m, n, o], {n, 1, nmax, 1}, {m, 1, mmaxTot, 1}, {o, 1, omax[[n]], 1}]
```

2/22/24 14:41:02 In[]:=

```
Export["J coup ratio 1.gif", Manipulate[aux[m, 1, 1] // Rasterize
   (*,{n,1,nmax,1}*), {m, 1, mmaxTot, 1}(*,{o,1,omax[[n]],1}*)]]
 Export["J coup ratio 2.gif", Manipulate[aux[2, n, 1] // Rasterize, {n, 1, nmax, 1}(*,{m,1,mmaxTo
Export["J coup ratio 3.gif", Manipulate[aux[2, nmax, o] // Rasterize(*,{n,1,nmax,1},{m,1,mmaxTot
```

2/22/24 14:42:19 In[]:=

```
Manipulate[{Jcoup[k, m, n, o] // MatrixForm,
   JcoupRatio[k, m, n, o] // MatrixForm} // Column,
 {m, 1, mmaxTot, 1}, {n, 1, nmax, 1}, {o, 1, omax[[n]], 1}]
```

We continue...

2/22/24 14:42:19 In[]:=

```
nmax
Clear@aux; jcoupnum = 5;
Clear@Fig2;
```

2/22/24 14:42:19 Out[]=

```
8
```

```
JcoupRatio[k, 3, 1, 1] (*check we have imported it correctly*)
```

2/22/24 14:42:19 Out[]=

```
{{1.40033, 0.000700771}, {1.40108, 0.000230916}, {1.40086, 0.00019383},
 {1.40103, 0.000690545}, {1.40178, 0.000196578}, {1.40156, 0.000151261}}
```

```
plotRange[j_] := {Jcoup[k, 1, 1, 1]〚j, 1〛 + 0.1 {-1, 1}, {-0.1, 1.1}};
frameticks[j_] := {Round[Range[Jcoup[k, 1, 1, 1]〚j, 1〛
      (*-0.2*), Jcoup[k, 1, 1, 1]〚j, 1〛(*+0.2*), 0.2], 0.01], None};
frTicks = {{All, None},
   {{(*{1,"2/3(3/2 × 1J15NH)/1J14NH"},*){1, "2/3 (3/2 × 1J15NH)/1/2 (2×1J14NH)"}, {2, "2/3 (3/2 × 1J15NH)/1/3 (3×1J14NH)"}(*,
     {4,"2/5(5/2 × 1J15NH)/1J14NH"}*), {3, "2/5 (5/2 × 1J15NH)/1/2 (2×1J14NH)"}, {4, "2/5 (5/2 × 1J15NH)/1/3 (3×1J14NH)"}}, None}};
(*We specificaly ignore the 52 Hz peak because it is too close to 50 Hz*)
pltRange = {All, {1.40095, 1.4014}};
pltRangeZoomOut = {All, {1.400, 1.403}};
aspRat = 1;
errγRat = Abs[-2.71261804 / 1.9337792] Sqrt[(0.0001/-2.71261804)^2 + (0.0001/1.9337792)^2];
Clear@graphicsLines;
Table[graphicsLines[m] = {
      Graphics[{Dashed, Table[Line[{{1, JcoupRatio[k, m, 1, 1]〚j, 1〛},
            {6, JcoupRatio[k, m, 1, 1]〚j, 1〛}}], {j, 1, 6}]〚{2, 3, 5, 6}〛}],
      Graphics[{Dashed, Red, Line[{{1, 1.4012}, {6, 1.4012}}]}],
      Graphics[Text[Style["Berkeley", FontSize → 14, Red], {2, 0.00001 + 1.4012}]],
      Graphics[{Dashed, Blue, Line[{{1, Abs[-27.126 / 19.338]}, {6, Abs[-27.126 / 19.338]}}]}],
      Graphics[{Opacity@0.02, Blue,
        Rectangle[{1, Abs[-27.126 / 19.338] - errγRat}, {6, Abs[-27.126 / 19.338] + errγRat}]}],
      Graphics[
       Text[Style["γ15 N/γ14 N", FontSize → 14, Blue], {2, 0.00001 + Abs[-27.126 / 19.338]}]]
     };, {m, mmaxTot}];
pltLeg = Placed[
   {"Mean and Standard Error", "Cumulative Distribution Function Fit"}, {Center, Bottom}];
markerSize = 16;
pltMark = {Style["▼", 1.2 markerSize], Style["●", markerSize],
   Style["■", markerSize], Style["◆", markerSize], Style["▲", markerSize],
   Style["○", markerSize], Style["□", markerSize], Style["◇", markerSize] };
frLabel = {None, "Ratio of (15N/14N)-1H J couplings"};
frameticks[1] = {{52.30, 52.40, 52.50}, None};
frameticks[2] = {{104.69, 104.79, 104.89}, None};
frameticks[3] = {{110.01, 110.11, 110.21}, None};
frameticks[4] = {{157.09, 157.19, 157.29}, None};
frameticks[5] = {{183.45, 183.55, 183.65}, None};
```

2/22/24 14:42:19 In[]:=

```
Table[Table[JcoupRatio[k, m, 1, 1][[j, 1]], {j, 1, 6}], {m, 1, 8}] // MatrixForm
```

2/22/24 14:42:19 Out[]//MatrixForm=

1.40141	1.40107	1.40107	1.40173	1.40138	1.40138
1.40094	1.40108	1.40103	1.4012	1.40134	1.40129
1.40033	1.40108	1.40086	1.40103	1.40178	1.40156
1.39901	1.40038	1.40019	1.39992	1.40129	1.4011
1.40061	1.40125	1.40135	1.40062	1.40126	1.40136
1.39981	1.40095	1.40114	1.39985	1.40099	1.40119
1.40046	1.4011	1.40114	1.40066	1.4013	1.40134
1.40038	1.40105	1.40099	1.40083	1.4015	1.40144

2/22/24 14:42:19 In[]:=

```
Clear[JcoupArray]
Print["Expected compiling time = " <>
  ToString[NumberForm[8.86/(25*2) (mmaxTot*nmax) / 60, 3] // N] <> " min"];
Monitor[
  Table[
   JcoupArray[k, m, n] = Table[Thread[{Sort@Table[Jcoup[k, m, n, o][[j, 1]], {o, omax[[n]]}],
        Table[i/omax[[n]], {i, 1, omax[[n]]}]}], {j, 1, jcoupnum}];
   Table[
    Fig2[k, 1, m, n, j] = ListPlot[{{#[[1]], #[[2]]} & /@ JcoupArray[k, m, n][[j]][[;; ;; s]]},
      PlotRange -> {{frameticks[j][[1, -1]], frameticks[j][[1, 1]]}, {-0.1, 1.1}},
      FrameLabel -> {"Frequency (Hz)", "1/n"}, Joined -> False,
      FrameTicks -> {{{0, 0.5, 1}, None}, frameticks[j]}, PlotMarkers -> {Automatic, Tiny}];
    Fig2ZoomOut[k, 1, m, n, j] = ListPlot[{{#[[1]], #[[2]]} & /@ JcoupArray[k, m, n][[j]][[;; ;; s]]},
      PlotRange -> {{frameticks[j][[1, -1]] - 1, frameticks[j][[1, 1]] + 1}, {-0.1, 1.1}},
      FrameLabel -> {"Frequency (Hz)", "1/n"}, Joined -> False,
      FrameTicks -> {{{0, 0.5, 1}, None}, Automatic}, PlotMarkers -> {Automatic, Tiny}];
    , {j, 1, jcoupnum}];
   , {n, nmax}, {m, mmaxTot}],
  "{m,n} = " <> ToString@{m, n} <> " of " <> ToString@omax[[n]] <> " scans"];
```

Expected compiling time = 0.26 min

2/22/24 14:42:35 In[]:=

```
Manipulate[{Fig2[k, 1, m, n, j], Fig2ZoomOut[k, 1, m, n, j]} // Row,
 {{n, nmax}, 1, nmax, 1}, {m, 1, mmaxTot, 1}, {j, 1, 5, 1}]
```

2/22/24 14:42:36 In[]:=

```
{Jcoup[k, mmaxTot, 4, omax[[4]]] // MatrixForm, JcoupArray[k, mmaxTot, 4] // MatrixForm} // Row
```

2/22/24 14:42:36 Out[]=

$$\begin{pmatrix} 52.4191 & 0.0356531 \\ 104.815 & 0.0110518 \\ 110.077 & 0.00845474 \\ 157.182 & 0.00918314 \\ 183.533 & 0.0129665 \end{pmatrix} \begin{pmatrix} \begin{pmatrix} 52.4039 \\ \frac{1}{4} \end{pmatrix} & \begin{pmatrix} 52.4191 \\ \frac{1}{2} \end{pmatrix} & \begin{pmatrix} 52.4526 \\ \frac{3}{4} \end{pmatrix} & \begin{pmatrix} 52.456 \\ 1 \end{pmatrix} \\ \begin{pmatrix} 104.815 \\ \frac{1}{4} \end{pmatrix} & \begin{pmatrix} 104.819 \\ \frac{1}{2} \end{pmatrix} & \begin{pmatrix} 104.82 \\ \frac{3}{4} \end{pmatrix} & \begin{pmatrix} 104.827 \\ 1 \end{pmatrix} \\ \begin{pmatrix} 110.077 \\ \frac{1}{4} \end{pmatrix} & \begin{pmatrix} 110.078 \\ \frac{1}{2} \end{pmatrix} & \begin{pmatrix} 110.078 \\ \frac{3}{4} \end{pmatrix} & \begin{pmatrix} 110.079 \\ 1 \end{pmatrix} \\ \begin{pmatrix} 157.172 \\ \frac{1}{4} \end{pmatrix} & \begin{pmatrix} 157.181 \\ \frac{1}{2} \end{pmatrix} & \begin{pmatrix} 157.182 \\ \frac{3}{4} \end{pmatrix} & \begin{pmatrix} 157.182 \\ 1 \end{pmatrix} \\ \begin{pmatrix} 183.517 \\ \frac{1}{4} \end{pmatrix} & \begin{pmatrix} 183.523 \\ \frac{1}{2} \end{pmatrix} & \begin{pmatrix} 183.526 \\ \frac{3}{4} \end{pmatrix} & \begin{pmatrix} 183.533 \\ 1 \end{pmatrix} \end{pmatrix}$$

Printed by Wolfram Mathematica Student Edition

2/22/24 14:42:36 In[]:=

```
Table[
  JcoupStat[k, m, 1] = {#[[1]], #[[2]]} & /@ Jcoup[k, m, 1, 1];
  JcoupRatioStat[k, m, 1] = {
    ((2/3) Jcoup[k, m, 1, 1][[3, 1]]) / (Jcoup[k, m, 1, 1][[1, 1]])
     {1, Sqrt[((Jcoup[k, m, 1, 1][[3, 2]] / Jcoup[k, m, 1, 1][[3, 1]])^2 + (Jcoup[k, m, 1, 1][[1, 2]] / Jcoup[k, m, 1, 1][[1, 1]])^2)]},
    ((2/3) Jcoup[k, m, 1, 1][[3, 1]]) / ((1/2) Jcoup[k, m, 1, 1][[2, 1]])
     {1, Sqrt[(Jcoup[k, m, 1, 1][[3, 2]] / Jcoup[k, m, 1, 1][[3, 1]])^2 + (Jcoup[k, m, 1, 1][[2, 2]] / Jcoup[k, m, 1, 1][[2, 1]])^2]},
    ((2/3) Jcoup[k, m, 1, 1][[3, 1]]) / ((1/3) Jcoup[k, m, 1, 1][[4, 1]])
     {1, Sqrt[(Jcoup[k, m, 1, 1][[3, 2]] / Jcoup[k, m, 1, 1][[3, 1]])^2 + (Jcoup[k, m, 1, 1][[4, 2]] / Jcoup[k, m, 1, 1][[4, 1]])^2]},
    ((2/5) Jcoup[k, m, 1, 1][[5, 1]]) / (Jcoup[k, m, 1, 1][[1, 1]])
     {1, Sqrt[((Jcoup[k, m, 1, 1][[5, 2]] / Jcoup[k, m, 1, 1][[5, 1]])^2 + (Jcoup[k, m, 1, 1][[1, 2]] / Jcoup[k, m, 1, 1][[1, 1]])^2)]},
    ((2/5) Jcoup[k, m, 1, 1][[5, 1]]) / ((1/2) Jcoup[k, m, 1, 1][[2, 1]])
     {1, Sqrt[(Jcoup[k, m, 1, 1][[5, 2]] / Jcoup[k, m, 1, 1][[5, 1]])^2 + (Jcoup[k, m, 1, 1][[2, 2]] / Jcoup[k, m, 1, 1][[2, 1]])^2]},
    ((2/5) Jcoup[k, m, 1, 1][[5, 1]]) / ((1/3) Jcoup[k, m, 1, 1][[4, 1]])
     {1, Sqrt[(Jcoup[k, m, 1, 1][[5, 2]] / Jcoup[k, m, 1, 1][[5, 1]])^2 + (Jcoup[k, m, 1, 1][[4, 2]] / Jcoup[k, m, 1, 1][[4, 1]])^2]}};, {m, mmaxTot}];
```

2/22/24 14:42:36 In[]:=

```
Monitor[
  Table[
    JcoupStat[k, m, n] = Table[{Mean@JcoupArray[k, m, n][[j, All, 1]],
       SEM@JcoupArray[k, m, n][[j, All, 1]]}, {j, 1, jcoupnum}];
    JcoupRatioStat[k, m, n] = {
      ((2/3) JcoupStat[k, m, n][[3, 1]])/(JcoupStat[k, m, n][[1, 1]])
       {1, Sqrt[(JcoupStat[k, m, n][[3, 2]]/JcoupStat[k, m, n][[3, 1]])^2 + (JcoupStat[k, m, n][[1, 2]]/JcoupStat[k, m, n][[1, 1]])^2]},
      ((2/3) JcoupStat[k, m, n][[3, 1]])/((1/2) JcoupStat[k, m, n][[2, 1]])
       {1, Sqrt[(JcoupStat[k, m, n][[3, 2]]/JcoupStat[k, m, n][[3, 1]])^2 + (JcoupStat[k, m, n][[2, 2]]/JcoupStat[k, m, n][[2, 1]])^2]},
      ((2/3) JcoupStat[k, m, n][[3, 1]])/((1/3) JcoupStat[k, m, n][[4, 1]])
       {1, Sqrt[(JcoupStat[k, m, n][[3, 2]]/JcoupStat[k, m, n][[3, 1]])^2 + (JcoupStat[k, m, n][[4, 2]]/JcoupStat[k, m, n][[4, 1]])^2]},
      ((2/5) JcoupStat[k, m, n][[5, 1]])/(JcoupStat[k, m, n][[1, 1]])
       {1, Sqrt[(JcoupStat[k, m, n][[5, 2]]/JcoupStat[k, m, n][[5, 1]])^2 + (JcoupStat[k, m, n][[1, 2]]/JcoupStat[k, m, n][[1, 1]])^2]},
      ((2/5) JcoupStat[k, m, n][[5, 1]])/((1/2) JcoupStat[k, m, n][[2, 1]])
       {1, Sqrt[(JcoupStat[k, m, n][[5, 2]]/JcoupStat[k, m, n][[5, 1]])^2 + (JcoupStat[k, m, n][[2, 2]]/JcoupStat[k, m, n][[2, 1]])^2]},
      ((2/5) JcoupStat[k, m, n][[5, 1]])/((1/3) JcoupStat[k, m, n][[4, 1]])
       {1, Sqrt[(JcoupStat[k, m, n][[5, 2]]/JcoupStat[k, m, n][[5, 1]])^2 + (JcoupStat[k, m, n][[4, 2]]/JcoupStat[k, m, n][[4, 1]])^2]}
     };
    , {n, 2, nmax}, {m, mmaxTot}];
  , "{m,n,o} = " <> ToString@{m, n, o} <> " of " <> ToString@omax[[n]] <> " scans"];
```

2/22/24 14:42:36 In[]:=

```
np = -1; q = 1;
jcoupnum = 5;
Monitor[Table[
   Clear@aux;
   aux[1] = Table[JcoupStat[k, m, n], {n, nmax}];
   aux[2] = Table[JcoupRatioStat[k, m, n], {n, nmax}];
   Export[FileNameJoin[{datafolderMain, "Answer to Reviewers v2",
      "JcoupStat[k," <> ToString[m] <> "].h5"}], aux[1], {"Datasets", "Dataset1"}];
   Export[FileNameJoin[{datafolderMain, "Answer to Reviewers v2",
      "JcoupRatioStat[k," <> ToString[m] <> "].h5"}], aux[2], {"Datasets", "Dataset1"}];
   Clear@aux;, {m, mmaxTot}], "{m} = " <> ToString@{m}];
Clear@aux;
```

2/22/24 14:42:37 In[]:=

```
Manipulate[JcoupStat[k, m, n] // MatrixForm, {m, 1, mmaxTot, 1}, {n, 1, nmax, 1}]
```

2/22/24 14:42:37 In[]:=

```
Clear@Fig3;
Table[Fig3[k, 1, m] = ListLogLogPlot[
    (Table[Thread@{ConstantArray[36000/omax[[n]], jcoupnum], 10^3 JcoupStat[k, m, n][[All, 2]]},
       {n, nmax}])^T, Joined → False,
    FrameLabel → {"Number of averaged scans", "Standard Error (mHz)"},
    PlotLegends → {"52 Hz", "105 Hz", "110 Hz", "157 Hz", "183 Hz"},
    Axes → True,
    ImagePadding → All,
    Frame → True,
    PlotRange → {{0.5, 1×10^5}, All}], {m, mmaxTot}];
```

2/22/24 14:42:37 In[]:=

```
Table[Fig3[k, 1, m] // Rasterize, {m, 4(*mmaxTot*)}] // Column
```

2/22/24 14:42:39 Out[]=

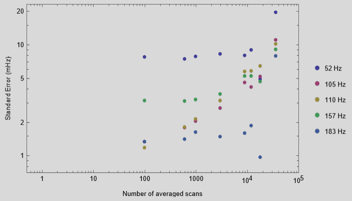

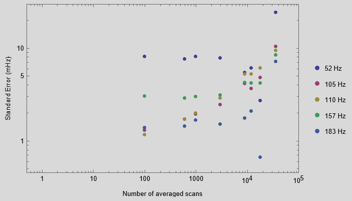

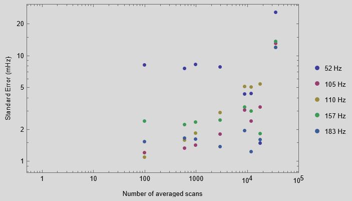

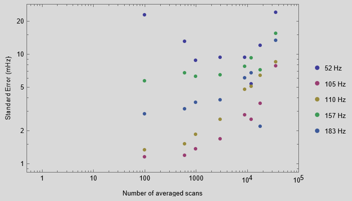

2/22/24 14:42:39 In[]:=

```
np = -1; q = 1;
Table[Export[FileNameJoin[{datafolderMain, "Answer to Reviewers v2",
      "Fig3[k,1," <> ToString[m] <> "].pdf"}], Fig3[k, 1, m][[1 ;; np ;; q]]];, {m, mmaxTot}];
```

2/22/24 14:42:54 In[]:=

```
Clear[a, g, μ, σ, gmax, rangeGroup, fit];
gmax = 0;
model = (*a[1]*)CDF[NormalDistribution[μ, σ], ν] (*+∑_{i=0}^{gmax} g[i] ν^i*)
```

2/22/24 14:42:54 Out[]=

$$\frac{1}{2}\,\text{Erfc}\left[\frac{\mu-\nu}{\sqrt{2}\,\sigma}\right]$$

2/22/24 14:42:54 In[]:=

```
JcoupArray[k, 2, 4] // MatrixForm
```

2/22/24 14:42:54 Out[]//MatrixForm=

(52.3891, 1/4)	(52.3895, 1/2)	(52.3975, 3/4)	(52.4123, 1)
(104.777, 1/4)	(104.786, 1/2)	(104.787, 3/4)	(104.797, 1)
(110.096, 1/4)	(110.113, 1/2)	(110.115, 3/4)	(110.12, 1)
(157.174, 1/4)	(157.19, 1/2)	(157.191, 3/4)	(157.193, 1)
(183.551, 1/4)	(183.553, 1/2)	(183.553, 3/4)	(183.559, 1)

2/22/24 14:42:54 In[]:=

```
Print["Expected compiling time = " <>
   ToString[NumberForm[34/(25 * (5 - 4) 5) (mmaxTot * (nmax - 4) * 5) / 60, 3] // N] <> " min"];
Monitor[Table[
     dataFit[k, 4, m, n, j] = Join[
       Select[JcoupArray[k, m, n][[j]],
        (Jcoup[k, m, 1, 1][[j, 1]] - 1) < #[[1]] < (Jcoup[k, m, 1, 1][[j, 1]] + 1) &]];
parameters[k, 4, m, n, j] = {(*a[1],*){μ, Jcoup[k, m, 1, 1][[j, 1]]}, {σ, 0.002}}
     (*~Join~(g/@Range[0,gmax])*);
fit[k, 4, m, n, j] = NonlinearModelFit[
        {#[[1]], Re[#[[2]]]} & /@ dataFit[k, 4, m, n, j],
        Re@model,
        parameters[k, 4, m, n, j], ν, MaxIterations → 1000] // Quiet;
s = 1;
     Table[
      Fig2[k, 2, m, i, j] = Fig2[k, 1, m, i, j];
      Fig2ZoomOut[k, 2, m, i, j] = Fig2ZoomOut[k, 1, m, i, j], {i, 1, 3}, {m, mmaxTot}];
     Fig2[k, 2, m, n, j] = Show[{
        Fig2[k, 1, m, n, j],
        Plot[fit[k, 4, m, n, j][ν], {ν, Jcoup[k, 2, 1, 1][[j, 1]] - 0.2,
          Jcoup[k, 2, 1, 1][[j, 1]] + 0.2}, PlotRange → All, PlotStyle → {Red, Opacity@0.5}]
       }];
     Fig2ZoomOut[k, 2, m, n, j] = Show[{
        Fig2ZoomOut[k, 1, m, n, j],
        Plot[fit[k, 4, m, n, j][ν], {ν, Jcoup[k, 2, 1, 1][[j, 1]] - 0.2,
          Jcoup[k, 2, 1, 1][[j, 1]] + 0.2}, PlotRange → All, PlotStyle → {Red, Opacity@0.5}]
       }];
     , {j, 1, jcoupnum}, {m, mmaxTot}, {n, 4, nmax}],
    "{m,n,j} = " <> ToString@{m, n, j} <> " of " <> ToString@omax[[n]] <> " scans"];;
```

```
Expected compiling time = 0.997 min
```

2/22/24 14:43:23 In[]:=

```
{Manipulate[fit[k, 4, m, n, j]["ParameterTable"], {m, 1, mmaxTot, 1},
   {n, 5, nmax, 1}, {j, 1, 5, 1}], Jcoup[k, 1, 1, 1] // MatrixForm} // Row
```

2/22/24 14:43:23 In[]:=

```
Manipulate[Table[Fig2[k, 2, m, nmax, j], {j, 5}] // Row, {m, 1, mmaxTot, 1}]
```

2/22/24 14:43:24 In[]:=

```
Table[fit[k, 4, m, 5, 1]["ParameterTable"] // MatrixForm, {m, mmaxTot}] // Quiet // Row
Table[fit[k, 4, m, nmax, 1]["RSquared"] // MatrixForm, {m, mmaxTot}]
```

2/22/24 14:43:24 Out[]=

	Estimate	Standard Error	t-Statistic	P-Value
μ	52.3697	0.00183075	28605.5	6.70811×10^{-41}
σ	0.0332158	0.00276608	12.0082	2.9026×10^{-7}

	Estimate	Standard Error	t-Statistic	P-Value
μ	52.3928	0.00208702	25104.1	2.47548×10^{-40}
σ	0.0322975	0.00309725	10.4278	1.08134×10^{-6}

	Estimate	Standard Error	t-Statistic	P-Value
μ	52.4064	0.00153032	34245.4	1.10937×10^{-41}
σ	0.0286033	0.00258945	11.0461	6.34086×10^{-7}

	Estimate	Standard Error	t-Statistic	P-Value
μ	52.4459	0.00186809	28074.6	8.09023×10^{-41}
σ	0.0234805	0.00363956	6.45146	0.0000733418

	Estimate	Standard Error	t-Statistic	P-Value
μ	52.4124	0.00228303	22957.4	6.05173×10^{-40}
σ	0.0431446	0.00421432	10.2376	1.28119×10^{-6}

	Estimate	Standard Error	t-Statistic	P-Value
μ	52.4453	0.00179391	29235.3	5.39545×10^{-41}
σ	0.00154942	0.00248182	0.624305	0.546406

	Estimate	Standard Error	t-Statistic	P-Value
μ	52.4484	0.000774149	67749.8	1.20787×10^{-44}
σ	0.0161705	0.00133183	12.1416	2.61613×10^{-7}

	Estimate	Standard Error	t-Statistic	P-Value
μ	52.3886	0.00316298	16563.1	1.58377×10^{-38}
σ	0.049651	0.00482658	10.287	1.2257×10^{-6}

	Estimate	Standard Error	t-Statistic	P-Value
μ	52.3773	0.00221095	23690.	4.42032×10^{-40}
σ	0.0534819	0.00364134	14.6874	4.27975×10^{-8}

	Estimate	Standard Error	t-Statistic	P-Value
μ	52.4749	3.83348×10^{27}	1.36886×10^{-26}	1
σ	0.002	4.70806×10^{28}	4.24804×10^{-32}	1

	Estimate	Standard Error	t-Statistic	P-Value
μ	52.4777	0.00130108	40334.	2.15967×10^{-42}
σ	0.0232375	0.00286712	8.10482	0.0000105042

2/22/24 14:43:24 Out[]=

```
{0.99942, 0.999323, 0.99964, 0.993862, 0.999707,
 0.999525, 0.999596, 0.999439, 0.99899, 0.996865, 0.99729}
```

a[1] → 4995.7	a[1] → 7636.35	a[1] → 0.999745	a[1] → 1.29044	a[1] → 0.984728
μ → 54.495	μ → 54.4938	μ → 52.4781	μ → 52.4023	μ → 52.4122
σ → 0.62799	σ → 0.598483	σ → 0.0289094	σ → 0.0703418	σ → 0.0270342
g[0] → -1.29821	g[0] → -1.22638	g[0] → -0.043281	g[0] → -0.268375	g[0] → 0.0735265

```
{0.996646, 0.994136, 0.994857, 0.996415, 0.994368}
```

2/22/24 14:43:24 In[]:=

```
Table[
  Fig2[k, 2, m] = Table[Fig2[k, 2, m, n, j], {n, nmax}, {j, 1, jcoupnum}];
  Fig2ZoomOut[k, 2, m] = Table[Fig2ZoomOut[k, 2, m, n, j], {n, nmax}, {j, 1, jcoupnum}];
  , {m, mmaxTot}];
{Manipulate[Fig2[k, 2, m] // GraphicsGrid // Rasterize, {m, 1, mmaxTot, 1}],
  Manipulate[Fig2ZoomOut[k, 2, m] // GraphicsGrid // Rasterize, {m, 1, mmaxTot, 1}]} // Row
```

2/22/24 14:43:24 In[]:=

```
Table[auxGif[m] = Fig2[k, 2, m] // GraphicsGrid // Rasterize, {m, 1, mmaxTot, 1}];
```

2/22/24 14:45:42 In[]:=

```
Export[FileNameJoin[{datafolderMain, "Answer to Reviewers v2", "Fig2[k,2,m].gif"}],
  Manipulate[auxGif[m], {m, 1, mmaxTot, 1}], "DisplayDurations" → 0.5];
```

2/22/24 14:46:16 In[]:=

```
np = -1; q = 1;
Monitor[Table[
   Export[FileNameJoin[{datafolderMain, "Answer to Reviewers v2",
      "Fig2[k,2," <> ToString[m] <> "].pdf"}], Fig2[k, 2, m][[1 ;; np ;; q]] // GraphicsGrid];
   (*Export[FileNameJoin[{datafolderMain,"Answer to Reviewers v2",
      "Fig2[k,2,"<>ToString[m]<>"][1].pdf"}],Fig2[k,2,m][[1;;4;;q]]//GraphicsGrid];
   Export[FileNameJoin[{datafolderMain,"Answer to Reviewers v2",
      "Fig2[k,2,"<>ToString[m]<>"][2].pdf"}],Fig2[k,2,m][[5;;nmax;;q]]//GraphicsGrid];*)
   (*Export[FileNameJoin[{datafolderMain,"Answer to Reviewers v2",
      "Fig2[k,2,"<>ToString[m]<>"][3].pdf"}],Fig2[k,2,m][[6;;9;;q]]//GraphicsGrid];
   Export[FileNameJoin[{datafolderMain,"Answer to Reviewers v2",
      "Fig2[k,2,"<>ToString[m]<>"][4].pdf"}],Fig2[k,2,m][[10;;11;;q]]//GraphicsGrid];
   Export[FileNameJoin[
     {datafolderMain,"Answer to Reviewers v2","Fig2[k,2,"<>ToString[m]<>"][final].pdf"}],
    Fig2[k,2,m][[{5,7,9,11}]]//GraphicsGrid];*)

   Export[FileNameJoin[{datafolderMain, "Answer to Reviewers v2", "Fig2ZoomOut[k,2," <>
       ToString[m] <> "].pdf"}], Fig2ZoomOut[k, 2, m][[1 ;; np ;; q]] // GraphicsGrid];
   (*Export[FileNameJoin[{datafolderMain,"Answer to Reviewers v2",
      "Fig2[k,2,"<>ToString[m]<>"][1].pdf"}],Fig2[k,2,m][[1;;4;;q]]//GraphicsGrid];
   Export[FileNameJoin[{datafolderMain,"Answer to Reviewers v2",
      "Fig2[k,2,"<>ToString[m]<>"][2].pdf"}],Fig2[k,2,m][[5;;nmax;;q]]//GraphicsGrid];*)
   (*Export[FileNameJoin[{datafolderMain,"Answer to Reviewers v2",
      "Fig2[k,2,"<>ToString[m]<>"][3].pdf"}],Fig2[k,2,m][[6;;9;;q]]//GraphicsGrid];
   Export[FileNameJoin[{datafolderMain,"Answer to Reviewers v2",
      "Fig2[k,2,"<>ToString[m]<>"][4].pdf"}],Fig2[k,2,m][[10;;11;;q]]//GraphicsGrid];
   Export[FileNameJoin[
     {datafolderMain,"Answer to Reviewers v2","Fig2[k,2,"<>ToString[m]<>"][final].pdf"}],
    Fig2[k,2,m][[{5,7,9,11}]]//GraphicsGrid];*)

   , {m, mmaxTot}], "m = " <> ToString@{m}];
```

2/22/24 14:51:54 In[]:=

```
Monitor[
 Table[
   JcoupCDF[k, m, n] = Abs@Table[{
       {fit[k, 4, m, n, j]["BestFitParameters"][[1, 2]],
        fit[k, 4, m, n, j]["ParameterErrors"][[1]]},
       1/Sqrt[omax[[n]]] {fit[k, 4, m, n, j]["BestFitParameters"][[2, 2]],
          fit[k, 4, m, n, j]["ParameterErrors"][[2]]}
      }, {j, 1, jcoupnum}];
```

```
JcoupRatioCDF[k, m, n] = {
    ((2/3) JcoupCDF[k, m, n]〚3, 1, 1〛) / (JcoupCDF[k, m, n]〚1, 1, 1〛)
     {1, Sqrt[(JcoupCDF[k, m, n]〚3, 1, 2〛 / JcoupCDF[k, m, n]〚3, 1, 1〛)^2 + (JcoupCDF[k, m, n]〚1, 1, 2〛 / JcoupCDF[k, m, n]〚1, 1, 1〛)^2],
      Sqrt[(JcoupCDF[k, m, n]〚3, 2, 1〛 / JcoupCDF[k, m, n]〚3, 1, 1〛)^2 + (JcoupCDF[k, m, n]〚1, 2, 1〛 / JcoupCDF[k, m, n]〚1, 1, 1〛)^2]},
    ((2/3) JcoupCDF[k, m, n]〚3, 1, 1〛) / ((1/2) JcoupCDF[k, m, n]〚2, 1, 1〛)
     {1, Sqrt[(JcoupCDF[k, m, n]〚3, 1, 2〛 / JcoupCDF[k, m, n]〚3, 1, 1〛)^2 + (JcoupCDF[k, m, n]〚2, 1, 2〛 / JcoupCDF[k, m, n]〚2, 1, 1〛)^2],
      Sqrt[(JcoupCDF[k, m, n]〚3, 2, 1〛 / JcoupCDF[k, m, n]〚3, 1, 1〛)^2 + (JcoupCDF[k, m, n]〚2, 2, 1〛 / JcoupCDF[k, m, n]〚2, 1, 1〛)^2]},
    ((2/3) JcoupCDF[k, m, n]〚3, 1, 1〛) / ((1/3) JcoupCDF[k, m, n]〚4, 1, 1〛)
     {1, Sqrt[(JcoupCDF[k, m, n]〚3, 1, 2〛 / JcoupCDF[k, m, n]〚3, 1, 1〛)^2 + (JcoupCDF[k, m, n]〚4, 1, 2〛 / JcoupCDF[k, m, n]〚4, 1, 1〛)^2],
      Sqrt[(JcoupCDF[k, m, n]〚3, 2, 1〛 / JcoupCDF[k, m, n]〚3, 1, 1〛)^2 + (JcoupCDF[k, m, n]〚4, 2, 1〛 / JcoupCDF[k, m, n]〚4, 1, 1〛)^2]},
    ((2/5) JcoupCDF[k, m, n]〚5, 1, 1〛) / (JcoupCDF[k, m, n]〚1, 1, 1〛)
     {1, Sqrt[(JcoupCDF[k, m, n]〚5, 1, 2〛 / JcoupCDF[k, m, n]〚5, 1, 1〛)^2 + (JcoupCDF[k, m, n]〚1, 1, 2〛 / JcoupCDF[k, m, n]〚1, 1, 1〛)^2],
      Sqrt[(JcoupCDF[k, m, n]〚5, 2, 1〛 / JcoupCDF[k, m, n]〚5, 1, 1〛)^2 + (JcoupCDF[k, m, n]〚1, 2, 1〛 / JcoupCDF[k, m, n]〚1, 1, 1〛)^2]},
    ((2/5) JcoupCDF[k, m, n]〚5, 1, 1〛) / ((1/2) JcoupCDF[k, m, n]〚2, 1, 1〛)
     {1, Sqrt[(JcoupCDF[k, m, n]〚5, 1, 2〛 / JcoupCDF[k, m, n]〚5, 1, 1〛)^2 + (JcoupCDF[k, m, n]〚2, 1, 2〛 / JcoupCDF[k, m, n]〚2, 1, 1〛)^2],
      Sqrt[(JcoupCDF[k, m, n]〚5, 2, 1〛 / JcoupCDF[k, m, n]〚5, 1, 1〛)^2 + (JcoupCDF[k, m, n]〚2, 2, 1〛 / JcoupCDF[k, m, n]〚2, 1, 1〛)^2]},
    ((2/5) JcoupCDF[k, m, n]〚5, 1, 1〛) / ((1/3) JcoupCDF[k, m, n]〚4, 1, 1〛)
     {1, Sqrt[(JcoupCDF[k, m, n]〚5, 1, 2〛 / JcoupCDF[k, m, n]〚5, 1, 1〛)^2 + (JcoupCDF[k, m, n]〚4, 1, 2〛 / JcoupCDF[k, m, n]〚4, 1, 1〛)^2],
```

```
          Sqrt[(JcoupCDF[k, m, n][[5, 2, 1]] / JcoupCDF[k, m, n][[5, 1, 1]])^2 + (JcoupCDF[k, m, n][[4, 2, 1]] / JcoupCDF[k, m, n][[4, 1, 1]])^2]}
     };
   , {m, mmaxTot}, {n, 5, nmax}],
  "{m,n} = " <> ToString@{m, n} <> " of " <> ToString@omax[[n]] <> " scans"];
```

2/22/24 14:52:07 In[]:=

```
Manipulate[
 {{JcoupStat[k, m, nmax] // MatrixForm,
     JcoupRatioStat[k, m, nmax] // MatrixForm} // Row,
   {JcoupCDF[k, m, nmax] // MatrixForm,
     JcoupRatioCDF[k, m, nmax] // MatrixForm} // Row} // Column,
 {m, 1, mmax, 1}]
```

2/22/24 14:52:07 In[]:=

```
np = -1; q = 1;
jcoupnum = 5;
Clear@aux;
Monitor[Table[
   aux[3] = Table[JcoupCDF[k, m, n], {n, 5, nmax}];
   aux[4] = Table[JcoupRatioCDF[k, m, n], {n, 5, nmax}];
   Export[FileNameJoin[{datafolderMain, "Answer to Reviewers v2",
      "JcoupCDF[k," <> ToString[m] <> "].h5"}], aux[3], {"Datasets", "Dataset1"}];
   Export[FileNameJoin[{datafolderMain, "Answer to Reviewers v2",
      "JcoupRatioCDF[k," <> ToString[m] <> "].h5"}], aux[4], {"Datasets", "Dataset1"}];
   Clear@aux;, {m, mmax}], "{m} = " <> ToString@{m}];
Clear@aux;
```

2/22/24 14:52:08 In[]:=

```
Clear@q; q = 1;
Monitor[Table[
   aux[1] = Import[FileNameJoin[
      {datafolderMain, "Answer to Reviewers v2", "JcoupCDF[k," <> ToString[m] <> "].h5"}],
     {"Datasets", "Dataset1"}, "ComplexKeys" → {"Re", "Im"}];
   aux[2] = Import[FileNameJoin[{datafolderMain,
       "Answer to Reviewers v2", "JcoupRatioCDF[k," <> ToString[m] <> "].h5"}],
     {"Datasets", "Dataset1"}, "ComplexKeys" → {"Re", "Im"}];
   Table[JcoupCDF[k, m, n] = {#⟦1⟧, #⟦2⟧} & /@ (aux[1]⟦n - 4⟧⟦;; ;; q⟧), {n, 5, nmax}];
   Table[
    JcoupRatioCDF[k, m, n] = {#⟦1⟧, #⟦2⟧, #⟦3⟧} & /@ (aux[2]⟦n - 4⟧⟦;; ;; q⟧), {n, 5, nmax}];

   aux[3] = Import[FileNameJoin[
      {datafolderMain, "Answer to Reviewers v2", "JcoupStat[k," <> ToString[m] <> "].h5"}],
     {"Datasets", "Dataset1"}, "ComplexKeys" → {"Re", "Im"}];
   aux[4] = Import[FileNameJoin[{datafolderMain,
       "Answer to Reviewers v2", "JcoupRatioStat[k," <> ToString[m] <> "].h5"}],
     {"Datasets", "Dataset1"}, "ComplexKeys" → {"Re", "Im"}];
   Table[JcoupStat[k, m, n] = {#⟦1⟧, #⟦2⟧} & /@ (aux[3]⟦n⟧⟦;; ;; q⟧), {n, nmax}];
   Table[JcoupRatioStat[k, m, n] = {#⟦1⟧, #⟦2⟧} & /@ (aux[4]⟦n⟧⟦;; ;; q⟧), {n, nmax}];,
   {m, mmax}], "{m} = " <> ToString@{m}];
Clear@aux
```

2/22/24 14:52:13 In[]:=

```
Table[
  Fig3[k, 2, m] = ListLogLogPlot[
    Table[Thread@{ConstantArray[36 000/omax[[n]], jcoupnum], 10^3 JcoupCDF[k, m, n][[All, 1, 2]]},
      {n, 5, nmax}]^T, Joined → False,
    FrameLabel → {"Number of averaged scans", "Standard Error (mHz)"},
    PlotLegends → {"52 Hz", "105 Hz", "110 Hz", "157 Hz", "183 Hz"},
    Axes → True,
    ImagePadding → All,
    Frame → True,
    PlotRange → {{0.5, 1×10^5 }, All}];
  Fig3[k, 3, m] = ListLogLogPlot[
    Table[Thread@{ConstantArray[36 000/omax[[n]], jcoupnum], 10^3 JcoupCDF[k, m, n][[All, 2, 1]]},
      {n, 5, nmax}]^T, Joined → False,
    FrameLabel → {"Number of averaged scans", "Standard Error (mHz)"},
    PlotLegends → {"52 Hz", "105 Hz", "110 Hz", "157 Hz", "183 Hz"},
    Axes → True,
    ImagePadding → All,
    Frame → True,
    PlotRange → {{0.5, 1×10^5 }, All}];
  , {m, mmax}];
Manipulate[{Fig3[k, 2, m] // Rasterize,
   Fig3[k, 3, m] // Rasterize} // Column, {m, 5, 1}]
```

2/22/24 14:52:14 In[]:=

```
np = -1; q = 1;
Table[
  Export[FileNameJoin[{datafolderMain, "Answer to Reviewers v2",
     "Fig3[k,2," <> ToString[m] <> "].pdf"}], Fig3[k, 2, m][[1 ;; np ;; q]]];
  Export[FileNameJoin[{datafolderMain, "Answer to Reviewers v2",
     "Fig3[k,3," <> ToString[m] <> "].pdf"}], Fig3[k, 3, m][[1 ;; np ;; q]]];
  , {m, mmax}];
```

2/22/24 14:52:32 In[]:=

```
Table[Fig4[k, 1, m] = ListLogLogPlot[Table[Thread@
      {ConstantArray[36000/omax[[n]], jcoupnum + 1], 10^3 JcoupRatio[k, m, 1, 1][[All, 2]]}, {n, 1}]^T,
    PlotMarkers → "☆",
    (*PlotStyle→"Rainbow",*)
    Joined → False,
    FrameLabel → {"Number of averaged scans", "(fit 36k scans) Standard Error (10^3)"},
    (*PlotLegends→{"(2/3(3/2 × 1J15NH))/(1J14NH)","(2/3(3/2 × 1J15NH))/(1/2(2x1J14NH))",
     "(2/3(3/2 × 1J15NH))/(1/3(3x1J14NH))" ,"(2/5(5/2 × 1J15NH))/(1J14NH)","(2/5(5/2 × 1J15NH))/(1/2(2x1J14NH))","(2/5(5/2 × 1J15NH))/(1/3(3x1J14NH))"},*)
    Axes → True,
    ImagePadding → All,
    Frame → True,
    PlotRange → {All, All}];, {m, mmax}];
Fig4[k, 1, 3]
```

2/22/24 14:52:32 Out[]=

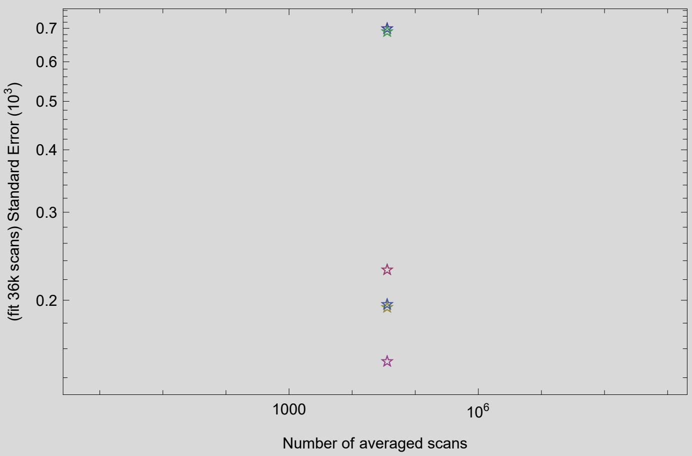

2/22/24 14:52:32 In[]:=

```
Table[
  Fig4[k, 2, m] = ListLogLogPlot[
    Table[Thread@{ConstantArray[36000/omax[[n]], jcoupnum + 1], 10^3 JcoupRatioStat[k, m, n][[All, 2]]},
      {n, 5, nmax}]^T, Joined → False,
    FrameLabel → {"Number of averaged scans", "(Statistical) Standard Error (10^3)"},
    PlotLegends → {"(2/3 (3/2 × 1 J15NH))/(1 J14NH)", "(2/3 (3/2 × 1 J15NH))/(1/2 (2x1 J14NH))", "(2/3 (3/2 × 1 J15NH))/(1/3 (3x1 J14NH))",
      "(2/5 (5/2 × 1 J15NH))/(1 J14NH)", "(2/5 (5/2 × 1 J15NH))/(1/2 (2x1 J14NH))", "(2/5 (5/2 × 1 J15NH))/(1/3 (3x1 J14NH))"},
    Axes → True,
    ImagePadding → All,
```

```
    Frame → True,
    PlotRange → {{0.5, 1×10^5}, {10^-2, 1}}];
  Fig4[k, 3, m] = ListLogLogPlot[
    Table[Thread@{ConstantArray[36000/omax[[n]], jcoupnum + 1], 10^3 JcoupRatioCDF[k, m, n][[All, 2]]},
      {n, 5, nmax}]^T, Joined → False,
    FrameLabel → {"Number of averaged scans", "(Statistical) Standard Error (10^3)"},
    PlotLegends → {"(2/3 (3/2 × ^1 J15NH))/(^1 J14NH)", "(2/3 (3/2 × ^1 J15NH))/(1/2 (2×^1 J14NH))", "(2/3 (3/2 × ^1 J15NH))/(1/3 (3×^1 J14NH))",
      "(2/5 (5/2 × ^1 J15NH))/(^1 J14NH)", "(2/5 (5/2 × ^1 J15NH))/(1/2 (2×^1 J14NH))", "(2/5 (5/2 × ^1 J15NH))/(1/3 (3×^1 J14NH))"},
    Axes → True,
    ImagePadding → All,
    Frame → True,
    PlotRange → {{0.5, 1×10^5}, {10^-2, 1}}];
  Fig4[k, 4, m] = ListLogLogPlot[
    Table[Thread@{ConstantArray[36000/omax[[n]], jcoupnum + 1], 10^3 JcoupRatioCDF[k, m, n][[All, 3]]},
      {n, 5, nmax}]^T, Joined → False,
    FrameLabel → {"Number of averaged scans", "(Sigmoide σ/ √N) Standard Error (10^3)"},
    PlotLegends → {"(2/3 (3/2 × ^1 J15NH))/(^1 J14NH)", "(2/3 (3/2 × ^1 J15NH))/(1/2 (2×^1 J14NH))", "(2/3 (3/2 × ^1 J15NH))/(1/3 (3×^1 J14NH))",
      "(2/5 (5/2 × ^1 J15NH))/(^1 J14NH)", "(2/5 (5/2 × ^1 J15NH))/(1/2 (2×^1 J14NH))", "(2/5 (5/2 × ^1 J15NH))/(1/3 (3×^1 J14NH))"},
    Axes → True,
    ImagePadding → All,
    (*PlotStyle→"Rainbow",*)
    Frame → True,
    PlotRange → {{0.5, 1×10^5}, {10^-2, 1}}];
  , {m, mmax}];
```

2/22/24 14:52:33 In[]:=

```
Manipulate[{Show[Fig4[k, 2, m], Fig4[k, 1, m]],
   (*Show[Fig4[k,3,m],Fig4[k,1,m]],*)
   Show[Fig4[k, 4, m], Fig4[k, 1, m]]} // Column, {m, 5, 1}]
```

2/22/24 14:52:33 In[]:=

```
np = -1; q = 1;
Table[
  Export[FileNameJoin[{datafolderMain, "Answer to Reviewers v2",
     "Fig4[k,2," <> ToString[m] <> "].pdf"}], Show[Fig4[k, 2, m], Fig4[k, 1, m]][[1 ;; np ;; q]]];
  Export[FileNameJoin[{datafolderMain, "Answer to Reviewers v2",
     "Fig4[k,3," <> ToString[m] <> "].pdf"}], Show[Fig4[k, 3, m], Fig4[k, 1, m]][[1 ;; np ;; q]]];
  Export[FileNameJoin[{datafolderMain, "Answer to Reviewers v2",
     "Fig4[k,4," <> ToString[m] <> "].pdf"}], Show[Fig4[k, 4, m], Fig4[k, 1, m]][[1 ;; np ;; q]]];
  , {m, mmax}];
```

2/22/24 14:53:03 In[]:=

```
Manipulate[NumberForm[{JcoupRatioStat[k, m, n] // MatrixForm,
     JcoupRatioCDF[k, m, n][[All, {1, 3}]] // MatrixForm} // Row, nmax] // Quiet,
 {m, 5, 1}, {n, nmax, 1}]
```

$N_0 = 36\,000;$

n (folder)	N_0 / N	N		$\left\|\frac{J_{^{15}NH}}{J_{^{14}NH}}\right\|$ (single scan fit)	$\left\|\frac{J_{^{15}NH}}{J_{^{14}NH}}\right\|$ (stat)	$\Big\|\frac{J_{^{15}}}{J_{^{14}}}$
1☆	1☆	36 000	$\frac{2/3\,(3/2\times{}^1J_{^{15}NH})}{{}^1J_{^{14}NH}}$<br>$\frac{2/3\,(3/2\times{}^1J_{^{15}NH})}{1/2\,(2\times{}^1J_{^{14}NH})}$<br>$\frac{2/3\,(3/2\times{}^1J_{^{15}NH})}{1/3\,(3\times{}^1J_{^{14}NH})}$<br>$\frac{2/5\,(5/2\times{}^1J_{^{15}NH})}{{}^1J_{^{14}NH}}$<br>$\frac{2/5\,(5/2\times{}^1J_{^{15}NH})}{1/2\,(2\times{}^1J_{^{14}NH})}$<br>$\frac{2/5\,(5/2\times{}^1J_{^{15}NH})}{1/3\,(3\times{}^1J_{^{14}NH})}$	1.40094 ± 0.000657166<br>1.40108 ± 0.000182699<br>1.40103 ± 0.000140827<br>1.4012 ± 0.00064863<br>1.40134 ± 0.0001486<br>1.40129 ± 0.0000923643	N.A.	
5	12	3000		N.A.	1.401 ± 0.000211363<br>1.40108 ± 0.0000489622<br>1.40102 ± 0.0000456259<br>1.40127 ± 0.000208563<br>1.40135 ± 0.000034714<br>1.40129 ± 0.0000298241	1.3472 ±<br>1.40116 ±<br>1.40103 ±<br>1.34743 ±<br>1.40139 ±<br>1.40127 ±
6	36	1000		N.A.	1.40102 ± 0.000217844<br>1.40108 ± 0.0000358695<br>1.40102 ± 0.0000364668<br>1.40129 ± 0.000216797<br>1.40135 ± 0.0000285241<br>1.40129 ± 0.0000292727	1.4011 ±<br>1.40112 ±<br>1.40109 ±<br>1.40135 ±<br>1.40137 ±<br>1.40133 ±
7	60	600		N.A.	1.40111 ± 0.00020283<br>1.40108 ± 0.0000313517<br>1.40102 ± 0.0000335006<br>1.40138 ± 0.000202008<br>1.40136 ± 0.0000251871<br>1.40129 ± 0.0000278179	1.4014 ±<br>1.40112 ±<br>1.40093 ±<br>1.40166 ±<br>1.40139 ±<br>1.40119 ±
8	360	100		N.A.	1.40159 ± 0.00021631<br>1.40108 ± 0.0000227192<br>1.40102 ± 0.0000306032<br>1.40187 ± 0.000216111<br>1.40136 ± 0.0000202946<br>1.40129 ± 0.000028852	1.40117 ±<br>1.40111 ±<br>1.40105 ±<br>1.40142 ±<br>1.40135 ±<br>1.4013 ±

9	1000	36		N.A.	1.40282 ± 0.000369387<br>1.40108 ± 0.0000207114<br>1.40101 ± 0.0000297866<br>1.4031 ± 0.000369378<br>1.40136 ± 0.0000191831<br>1.40129 ± 0.0000287483	1.40133 ±<br>1.40107 ±<br>1.40104 ±<br>1.40159 ±<br>1.40133 ±<br>1.4013 ±
10	2250	16		N.A.	1.40351 ± 0.000547167<br>1.40107 ± 0.0000196869<br>1.40109 ± 0.0000602659<br>1.4038 ± 0.000547256<br>1.40136 ± 0.0000190627<br>1.40137 ± 0.0000600759	1.40224 ±<br>1.40107 ±<br>1.40099 ±<br>1.40249 ±<br>1.40133 ±<br>1.40124 ±
11	3600	10		N.A.	1.40368 ± 0.000678682<br>1.40108 ± 0.000019653<br>1.40193 ± 0.000239437<br>1.40397 ± 0.000678811<br>1.40137 ± 0.000019207<br>1.40223 ± 0.00023945	1.40295 ±<br>1.40109 ±<br>1.401 ±<br>1.40321 ±<br>1.40134 ±<br>1.40125 ±

(**Appropiate number of significant digits**)

$N_0 = 36\,000;$

n (folder)	N_0/N	N		$\left\lvert\frac{J_{15_{NH}}}{J_{14_{NH}}}\right\rvert$ (single scan fit)	$\left\lvert\frac{J_{15_{NH}}}{J_{14_{NH}}}\right\rvert$ (stat)	$\left\lvert\frac{J_{15_{NH}}}{J_{14_{NH}}}\right\rvert$ (CDF)
1☆	1☆	36 000	$\frac{2/3\,(3/2\times{}^1J_{15_{NH}})}{{}^1J_{14_{NH}}}$<br>$\frac{2/3\,(3/2\times{}^1J_{15_{NH}})}{1/2\,(2\times{}^1J_{14_{NH}})}$<br>$\frac{2/3\,(3/2\times{}^1J_{15_{NH}})}{1/3\,(3\times{}^1J_{14_{NH}})}$<br>$\frac{2/5\,(5/2\times{}^1J_{15_{NH}})}{{}^1J_{14_{NH}}}$<br>$\frac{2/5\,(5/2\times{}^1J_{15_{NH}})}{1/2\,(2\times{}^1J_{14_{NH}})}$<br>$\frac{2/5\,(5/2\times{}^1J_{15_{NH}})}{1/3\,(3\times{}^1J_{14_{NH}})}$	1.4009 (7)<br>1.40108 (18)<br>1.40103 (14)<br>1.4012 (6)<br>1.40134 (15)<br>1.40129 (9)	N.A.	N.A.
5	12	3000		N.A.	1.40100 (21)<br>1.40108 (5)<br>1.40102 (5)<br>1.40127 (21)<br>1.40135 (3)<br>1.40129 (3)	1.347 (4)<br>1.40116 (4)<br>1.401034 (15)<br>1.347 (4)<br>1.40139 (4)<br>1.401266 (15)
6	36	1000		N.A.	1.40102 (22)<br>1.40108 (4)<br>1.40102 (4)<br>1.40129 (22)<br>1.40135 (3)<br>1.40129 (3)	1.4011 (3)<br>1.401120 (24)<br>1.40109 (3)<br>1.40135 (3)<br>1.401365 (22)<br>1.40133 (3)
7	60	600		N.A.	1.40111 (20)<br>1.40108 (3)<br>1.40102 (3)<br>1.40138 (20)<br>1.401355 (25)<br>1.40129 (3)	1.40140 (20)<br>1.401124 (24)<br>1.40093 (5)<br>1.401660 (20)<br>1.401386 (20)<br>1.40119 (5)
8	360	100		N.A.	1.40159 (22)<br>1.401082 (23)<br>1.40102 (3)<br>1.40187 (22)	1.40117 (21)<br>1.401109 (21)<br>1.40105 (3)<br>1.40142 (21)

				1.401360 (20)<br>1.40129 (3)	1.401352 (20)<br>1.40130 (3)
9	1000	36	N.A.	1.4028 (4)<br>1.401080 (20)<br>1.40101 (3)<br>1.4031 (4)<br>1.401362 (19)<br>1.40129 (3)	1.40133 (19)<br>1.401073 (20)<br>1.40104 (3)<br>1.40159 (19)<br>1.401332 (18)<br>1.40130 (3)
10	2250	16	N.A.	1.4035 (5)<br>1.401075 (20)<br>1.40109 (6)<br>1.4038 (5)<br>1.401361 (19)<br>1.40137 (6)	1.40224 (20)<br>1.401071 (19)<br>1.40099 (3)<br>1.40249 (20)<br>1.401326 (18)<br>1.40124 (3)
11	3600	10	N.A.	1.40368 (7)<br>1.401075 (20)<br>1.40193 (24)<br>1.40397 (7)<br>1.401368 (19)<br>1.40223 (24)	1.40295 (19)<br>1.401089 (19)<br>1.40010 (3)<br>1.40321 (19)<br>1.401343 (19)<br>1.40125 (3)

2/22/24 14:53:03 In[]:=

```
Clear@Fig5;
Table[Fig5[k, m, n, 1] = Show[{
    ListPlot[
     MapThread[{#1, Around[#2, #3]} &,
      {
       Range[1, 6],
       #[[1]] & /@ JcoupRatioStat[k, m, n],
       #[[2]] & /@ JcoupRatioStat[k, m, n]
      }
     ]
     , Joined → False,
     PlotMarkers → pltMark,
     FrameLabel → frLabel,
     FrameTicks → {{All, None}, {{{1, "(2/3 (3/2 × 1J15NH))/(1J14NH)"},
         {2, "(2/3 (3/2 × 1J15NH))/(1/2 (2×1J14NH))"}, {3, "(2/3 (3/2 × 1J15NH))/(1/3 (3×1J14NH))"}, {4, "(2/5 (5/2 × 1J15NH))/(1J14NH)"},
         {5, "(2/5 (5/2 × 1J15NH))/(1/2 (2×1J14NH))"}, {6, "(2/5 (5/2 × 1J15NH))/(1/3 (3×1J14NH))"}}, None}},
     ImagePadding → All,
     Frame → True,
     PlotRange → pltRangeZoomOut,
     ImageSize → Large,
     PlotStyle → ColorData[1, "ColorList"][[1]],
     PlotLegends → pltLeg],
    graphicsLines[m]
   }], {m, mmaxTot}, {n, nmax}];
```

```
Table[Fig5[k, m, n, 2] = Show[{
    ListPlot[
     MapThread[{#1, Around[#2, #3]} &,
      {
       Range[1, 6],
       #[[1]] & /@ JcoupRatioCDF[k, m, n],
       #[[3]] & /@ JcoupRatioCDF[k, m, n]
      }
     ]
     , Joined → False,
     PlotMarkers → pltMark,
     FrameLabel → frLabel,
     FrameTicks → {{All, None}, {{{1, "(2/3 (3/2 × ¹J15NH))/(¹J14NH)"},
         {2, "(2/3 (3/2 × ¹J15NH))/(1/2 (2×¹J14NH))"}, {3, "(2/3 (3/2 × ¹J15NH))/(1/3 (3×¹J14NH))"}, {4, "(2/5 (5/2 × ¹J15NH))/(¹J14NH)"},
         {5, "(2/5 (5/2 × ¹J15NH))/(1/2 (2×¹J14NH))"}, {6, "(2/5 (5/2 × ¹J15NH))/(1/3 (3×¹J14NH))"}}, None}},
     Axes → True,
     ImagePadding → All,
     Frame → True,
     PlotRange → pltRangeZoomOut,
     ImageSize → Large,
     PlotLegends → Placed[{"Cumulative Distribution Function Fit"}, {Center, Bottom}]],
    graphicsLines[m]
   }], {m, mmaxTot}, {n, 5, nmax}];
```

2/22/24 14:53:08 In[]:=

```
Table[Fig5[k, m] = Table[Fig5[k, m, n, j], {n, nmax}, {j, 1, 2}];, {m, mmaxTot}];
Manipulate[{Fig5[k, m, n, 1], Fig5[k, m, n, 2]} // Row,
 {m, 1, mmaxTot, 1}, {{n, nmax}, 1, nmax, 1}]
```

(*We chose partition 8 for the plot of the paper*)

2/22/24 14:53:08 In[]:=

```
Table[
  Export[FileNameJoin[
     {datafolderMain, "Answer to Reviewers v2", "Fig5[k," <> ToString[m] <> "][1].pdf"}],
    GraphicsGrid[Fig5[k, m][[1 ;; 4 ;; q]], ImageSize → Large];
  Export[FileNameJoin[
     {datafolderMain, "Answer to Reviewers v2", "Fig5[k," <> ToString[m] <> "][2].pdf"}],
    GraphicsGrid[Fig5[k, m][[5 ;; nmax ;; q]], ImageSize → Large];
  (*Export[FileNameJoin[
      {datafolderMain,"Answer to Reviewers v2","Fig5[k,"<>ToString[m]<>"][3].pdf"}],
     GraphicsGrid[Fig5[k,m][[9;;11;;q]],ImageSize→Large];*), {m, mmax}];
```

2/22/24 14:53:41 In[]:=

```
Export["anim.gif",
 Table[Fig5[k, m, nmax, 1], {m, 5}], "DisplayDurations" → 1
]
```

2/22/24 14:53:42 In[]:=

```
Table[Fig6old[k, m, 8] = Show[{
      ListPlot[
       {
        {{1, 1.401082 ± 0.000023},
         {2, 1.401017 ± 0.000031}, {3, 1.401360 ± 0.000020}, {4, 1.401294 ± 0.000029}}
        ,
        {{1, 1.401109 ± 0.000021},
         {2, 1.401052 ± 0.000029}, {3, 1.401352 ± 0.000020}, {4, 1.401296 ± 0.000029}}
       }
       , Joined → False,
       PlotMarkers → pltMark,
       FrameLabel → frLabel,
       PlotLabel → "{m,n} = " <> ToString[{m, 8}],
       FrameTicks → frTicks,
       Axes → True,
       ImagePadding → All,
       Frame → True,
       (*AspectRatio→aspRat,*)
       PlotRange → pltRange,
       ImageSize → Large,
       PlotLegends → pltLeg],
      graphicsLines[m]
     }];
   Fig6[k, m, n] = Show[{
      ListPlot[
       {
        MapThread[{#1, Around[#2, #3]} &,
          {
           {1, 1, 2, 3, 3, 4},
           #[[1]] & /@ JcoupRatioStat[k, m, n],
           #[[2]] & /@ JcoupRatioStat[k, m, n]
          }
         ][[{2, 3, 5, 6}]],
        MapThread[{#1, Around[#2, #3]} &,
          {
           {1, 1, 2, 3, 3, 4},
           #[[1]] & /@ JcoupRatioCDF[k, m, n],
           #[[3]] & /@ JcoupRatioCDF[k, m, n]
          }
         ][[{2, 3, 5, 6}]]
       }
       , Joined → False,
       PlotMarkers → pltMark,
       FrameLabel → frLabel,
```

```
        PlotLabel → "{m,n} = " <> ToString[{m, nmax}],
        FrameTicks → frTicks,
        Axes → True,
        ImagePadding → All,
        Frame → True,
        (*AspectRatio→aspRat,*)
        PlotRange → pltRange,
        ImageSize → Large,
        PlotLegends → pltLeg,
        PlotStyle → {{ColorData[1, "ColorList"][[1]], Opacity@0.75},
          {ColorData[1, "ColorList"][[2]], Opacity@0.75}}],
       graphicsLines[m]
      }];, {m, mmaxTot}, {n, nmax}] // Quiet;
Table[Fig6oldZoomOut[k, m, 8] = Show[{
       ListPlot[
        {
         {{1, 1.401082 ± 0.000023},
          {2, 1.401017 ± 0.000031}, {3, 1.401360 ± 0.000020}, {4, 1.401294 ± 0.000029}}
         ,
         {{1, 1.401109 ± 0.000021},
          {2, 1.401052 ± 0.000029}, {3, 1.401352 ± 0.000020}, {4, 1.401296 ± 0.000029}}
        }
        , Joined → False,
        PlotMarkers → pltMark,
        FrameLabel → frLabel,
        PlotLabel → "{m,n} = " <> ToString[{m, 8}],
        FrameTicks → frTicks,
        Axes → True,
        ImagePadding → All,
        Frame → True,
        (*AspectRatio→aspRat,*)
        PlotRange → pltRangeZoomOut,
        ImageSize → Large,
        PlotLegends → pltLeg],

       graphicsLines[m]
      }];
   Fig6ZoomOut[k, m, n] = Show[{
       ListPlot[
        {
         MapThread[{#1, Around[#2, #3]} &,
           {
            (*Range@6*){1, 1, 2, 3, 3, 4},
            #[[1]] & /@ JcoupRatioStat[k, m, n],
            #[[2]] & /@ JcoupRatioStat[k, m, n]
           }
          ][[{2, 3, 5, 6}]],
         MapThread[{#1, Around[#2, #3]} &,
           {
            (*Range@6*){1, 1, 2, 3, 3, 4},
            #[[1]] & /@ JcoupRatioCDF[k, m, n],
```

```
            #[[3]] & /@ JcoupRatioCDF[k, m, n]
           }
         ][[{2, 3, 5, 6}]]
        }
        , Joined → False,
        PlotMarkers → pltMark,
        FrameLabel → frLabel,
        PlotLabel → "{m,n} = " <> ToString[{m, nmax}],
        FrameTicks → frTicks,
        Axes → True,
        ImagePadding → All,
        Frame → True,
        (*AspectRatio→aspRat,*)
        PlotRange → pltRangeZoomOut,
        ImageSize → Large,
        PlotLegends → pltLeg,
        PlotStyle → {{ColorData[1, "ColorList"][[1]], Opacity@0.75},
          {ColorData[1, "ColorList"][[2]], Opacity@0.75}}],
       graphicsLines[m]
      }];, {m, mmaxTot}, {n, nmax}] // Quiet;
```

2/22/24 14:53:55 In[]:=

```
Manipulate[{(*Fig6old[k,1,8],*)Fig6[k, m, n], Fig6ZoomOut[k, m, n]} // Row,
 {m, 1, mmaxTot, 1}, {{n, nmax}, 5, nmax, 1}]
```

2/22/24 14:53:55 In[]:=

```
Export["anim.gif",
 Table[Fig6[k, m, nmax], {m, mmaxTot}], "DisplayDurations" → 1
]; Export["anim2.gif",
 Table[Fig6ZoomOut[k, m, nmax], {m, mmaxTot}], "DisplayDurations" → 1
];
```

(*We chose partition 8 for the plot*)

2/22/24 14:54:01 In[]:=

```
Table[
  Export[FileNameJoin[{datafolderMain, "Answer to Reviewers v2",
     "Fig6ZoomOut[k," <> ToString[m] <> "," <> ToString[nmax] <> "].pdf"}],
   Fig6ZoomOut[k, m, nmax], ImageSize → Large];
  Export[FileNameJoin[{datafolderMain, "Answer to Reviewers v2", "Fig6[k," <> ToString[m] <>
      "," <> ToString[nmax] <> "].pdf"}], Fig6[k, m, nmax], ImageSize → Large];
  , {m, mmaxTot}];
```

2/22/24 14:54:10 In[]:=

```
Export[FileNameJoin[
   {datafolderMain, "Answer to Reviewers v2", "Fig6ZoomOut[k," <> ToString[nmax] <> "].gif"}],
  Table[Fig6ZoomOut[k, m, nmax], {m, mmaxTot}], "DisplayDurations" → 0.5];
Export[FileNameJoin[
   {datafolderMain, "Answer to Reviewers v2", "Fig6[k," <> ToString[nmax] <> "].gif"}],
  Table[Fig6[k, m, nmax], {m, mmaxTot}], "DisplayDurations" → 0.5];
```

2/22/24 14:54:17 In[]:=

```
Export[FileNameJoin[{datafolderMain, "Answer to Reviewers v2",
    "Fig6[k,m=" <> ToString@Range@mmaxTot <> "," <> ToString[nmax] <> "].pdf"}],
  Table[Fig6[k, m, nmax], {m, mmaxTot}] // Column, ImageSize → Large];
Export[FileNameJoin[{datafolderMain, "Answer to Reviewers v2",
    "Fig6ZoomOut[k,m=" <> ToString@Range@mmaxTot <> "," <> ToString[nmax] <> "].pdf"}],
  Table[Fig6ZoomOut[k, m, nmax], {m, mmaxTot}] // Column, ImageSize → Large];
```

(*Here I construct the systematic error by the standard error of the different m baseline procedures. We only do it for a selection of reasonable baseline cleaning procedures*)

2/22/24 14:54:20 In[]:=

```
Clear[aux, JcoupRatioSys];
mSelection = {1, 2, 5, 6, 7} ~ Join ~ Range[8, 13]
mSelection // Length
Table[aux[1, n] = Table[STD[Table[JcoupRatioStat[k, m, n][[l, 1]], {m, mSelection // Length}]],
    {l, JcoupRatioStat[k, 1, 1] // Length}], {n, nmax}];
Table[
  JcoupRatioSys[1][k, m, n] = Thread[{JcoupRatioStat[k, m, n][[ ;; , 1]], aux[1, n]}];
  , {k, 3, 3}, {m, mmaxTot}, {n, nmax}];
Table[aux[2, n] = Table[STD[Table[JcoupRatioCDF[k, m, n][[l, 1]], {m, mSelection // Length}]],
    {l, JcoupRatioCDF[k, 1, 5] // Length}], {n, 5, nmax}];
Table[
  JcoupRatioSys[2][k, m, n] = Thread[{JcoupRatioCDF[k, m, n][[ ;; , 1]], aux[2, n]}];
  , {k, 3, 3}, {m, mmaxTot}, {n, 5, nmax}];
```

2/22/24 14:54:20 Out[]=

```
{1, 2, 5, 6, 7, 8, 9, 10, 11, 12, 13}
```

2/22/24 14:54:20 Out[]=

```
11
```

2/22/24 14:54:20 In[]:=

```
Manipulate[{JcoupRatioStat[k, m, n][[{2, 3, 5, 6}]] // MatrixForm,
    JcoupRatioSys[1][k, m, n][[{2, 3, 5, 6}]] // MatrixForm,
    JcoupRatioCDF[k, m, n][[{2, 3, 5, 6}]] // MatrixForm,
    JcoupRatioSys[2][k, m, n][[{2, 3, 5, 6}]] // MatrixForm} // Row,
  {{m, 2}, 1, mmaxTot, 1}, {{n, nmax}, 1, nmax, 1}] // Quiet
```

2/22/24 14:54:20 In[]:=

```
Table[
   Fig8[k, m, n] = Show[{
      ListPlot[
       {
        MapThread[{#1, Around[#2, #3]} &,
          {
           (*Range@6*){1, 1, 2, 3, 3, 4},
           #[[1]] & /@ JcoupRatioSys[1][k, m, n],
           #[[2]] & /@ JcoupRatioSys[1][k, m, n]
          }
         ][[{2, 3, 5, 6}]],
        MapThread[{#1, Around[#2, #3]} &,
```

```
          {
           {1, 1, 2, 3, 3, 4},
           #[[1]] & /@ JcoupRatioSys[2][k, m, n],
           #[[2]] & /@ JcoupRatioSys[2][k, m, n]
          }
         ][[{2, 3, 5, 6}]]
       }
       , Joined → False,
       PlotMarkers → pltMark,
       FrameLabel → frLabel,
       PlotLabel → "{m,n} = " <> ToString[{m, nmax}],
       FrameTicks → frTicks,
       Axes → True,
       ImagePadding → All,
       Frame → True,
       (*AspectRatio→aspRat,*)
       PlotRange → pltRange,
       ImageSize → Large,
       PlotLegends → pltLeg,
       PlotStyle → {{ColorData[1, "ColorList"][[1]], Opacity@0.75},
         {ColorData[1, "ColorList"][[2]], Opacity@0.75}}],
      graphicsLines[m]
     }];
  Fig8ZoomOut[k, m, n] = Show[{
      ListPlot[
       {
        MapThread[{#1, Around[#2, #3]} &,
          {
           {1, 1, 2, 3, 3, 4},
           #[[1]] & /@ JcoupRatioSys[1][k, m, n],
           #[[2]] & /@ JcoupRatioSys[1][k, m, n]
          }
         ][[{2, 3, 5, 6}]],
        MapThread[{#1, Around[#2, #3]} &,
          {
           {1, 1, 2, 3, 3, 4},
           #[[1]] & /@ JcoupRatioSys[2][k, m, n],
           #[[2]] & /@ JcoupRatioSys[2][k, m, n]
          }
         ][[{2, 3, 5, 6}]]
       }
       , Joined → False,
       PlotMarkers → pltMark,
       FrameLabel → frLabel,
       PlotLabel → "{m,n} = " <> ToString[{m, nmax}],
       FrameTicks → frTicks,
       Axes → True,
       ImagePadding → All,
       Frame → True,
       (*AspectRatio→aspRat,*)
       PlotRange → pltRangeZoomOut,
```

```
        ImageSize → Large,
        PlotLegends → pltLeg,
        PlotStyle → {{ColorData[1, "ColorList"][[1]], Opacity@0.75},
           {ColorData[1, "ColorList"][[2]], Opacity@0.75}}],
       graphicsLines[m]
      }];
    , {m, mmaxTot}, {n, nmax}] // Quiet;
```

2/22/24 14:54:26 In[]:=

```
Manipulate[{{Fig6[k, m, n], Fig8[k, m, n]},
   {Fig6ZoomOut[k, m, n], Fig8ZoomOut[k, m, n]}
  } // MatrixForm, {{m, 2}, 1, mmaxTot, 1}, {{n, nmax}, 1, nmax, 1}]
```

2/22/24 14:54:26 In[]:=

```
Print[
 {
   {"Systematic Error \n Statistical",
     {#[[2]]} & /@ JcoupRatioSys[1][k, m, 8][[{2, 3, 5, 6}]] // MatrixForm} // MatrixForm,
   {"Systematic Error \n CDF",
     {#[[2]]} & /@ JcoupRatioSys[2][k, m, 8][[{2, 3, 5, 6}]] // MatrixForm} // MatrixForm,
   Table[{
        "m = " <> ToString@m,
        {"Statistical",
          {#[[1]] ± #[[2]]} & /@ JcoupRatioStat[k, m, 8][[{2, 3, 5, 6}]] // MatrixForm} // MatrixForm,
        {"CDF",
          {#[[1]] ± #[[3]]} & /@ JcoupRatioCDF[k, m, 8][[{2, 3, 5, 6}]] // MatrixForm} // MatrixForm
      } // Row, {m, mmaxTot}] // Column,
   {"Systematic Error \n Statistical",
     {#[[2]]} & /@ JcoupRatioSys[1][k, m, 8][[{2, 3, 5, 6}]] // MatrixForm} // MatrixForm,
   {"Systematic Error \n CDF",
     {#[[2]]} & /@ JcoupRatioSys[2][k, m, 8][[{2, 3, 5, 6}]] // MatrixForm} // MatrixForm,
   Table[{
        "m = " <> ToString@m,
        {"Statistical", {NumberForm[#[[1]], 6] ± RoundR@#[[2]]} & /@
            JcoupRatioStat[k, m, 8][[{2, 3, 5, 6}]] // MatrixForm} // MatrixForm,
        {"CDF", {NumberForm[#[[1]], 6] ± RoundR@#[[3]]} & /@
            JcoupRatioCDF[k, m, 8][[{2, 3, 5, 6}]] // MatrixForm} // MatrixForm
      } // Row, {m, mmaxTot}] // Column
  } // Row
]
```

(*Below we find the results of all baseline procedures, and to the right the same thing is shown with appropriate significant digits*)

```
( Systematic Error        ) ( Systematic Error        )
(  Statistical            ) (  CDF                    )
(   ( 0.000349159 )       ) (   ( 0.000359551 )       )
(   ( 0.000298894 )       ) (   ( 0.000308335 )       )
(   (  0.00027212 )       ) (   (  0.00028227 )       )
(   (  0.00013785 )       ) (   ( 0.000119998 )       )

( Statistical                      ) ( CDF                              )
( ( 1.40107 ± 0.0000232464 )       ) ( ( 1.40108 ± 0.0000222154 )       )
```

m	Statistical	CDF
m = 1	1.40106 ± 0.0000316259<br>1.4014 ± 0.0000204902<br>1.40139 ± 0.0000296626	1.40107 ± 0.0000309679<br>1.4014 ± 0.0000204878<br>1.40139 ± 0.0000297565
m = 2	1.40108 ± 0.00002272<br>1.40102 ± 0.0000306035<br>1.40136 ± 0.0000202951<br>1.40129 ± 0.0000288521	1.4011 ± 0.0000218178<br>1.40103 ± 0.0000298639<br>1.40136 ± 0.000020351<br>1.4013 ± 0.0000288126
m = 3	1.40108 ± 0.0000210548<br>1.40086 ± 0.0000253687<br>1.4018 ± 0.0000197788<br>1.40157 ± 0.0000243247	1.4011 ± 0.0000200195<br>1.40087 ± 0.0000248071<br>1.4018 ± 0.0000195025<br>1.40157 ± 0.0000243963
m = 4	1.40039 ± 0.0000226602<br>1.40031 ± 0.0000529619<br>1.40131 ± 0.0000264668<br>1.40124 ± 0.0000547263	1.40039 ± 0.0000225853<br>1.40023 ± 0.000052432<br>1.40133 ± 0.0000259561<br>1.40117 ± 0.0000539972
m = 5	1.40124 ± 0.0000280728<br>1.40135 ± 0.0000333888<br>1.40125 ± 0.0000286082<br>1.40136 ± 0.0000338404	1.40125 ± 0.0000271021<br>1.40135 ± 0.0000330331<br>1.40125 ± 0.0000274288<br>1.40135 ± 0.0000333017
m = 6	1.40098 ± 0.0000178635<br>1.40119 ± 0.0000310322<br>1.40106 ± 0.0000179157<br>1.40128 ± 0.0000310634	1.40096 ± 0.0000170557<br>1.4012 ± 0.0000325105<br>1.40104 ± 0.0000173403<br>1.40127 ± 0.000032662
m = 7	1.40113 ± 0.0000181946<br>1.40116 ± 0.0000246531<br>1.40137 ± 0.0000189995<br>1.4014 ± 0.0000252549	1.40111 ± 0.0000171925<br>1.40116 ± 0.0000255782<br>1.40135 ± 0.0000182686<br>1.4014 ± 0.0000263159
m = 8	1.40118 ± 0.0000277084<br>1.40106 ± 0.000041976<br>1.40162 ± 0.000027166<br>1.4015 ± 0.0000416277	1.40118 ± 0.0000275001<br>1.40107 ± 0.0000417145<br>1.40162 ± 0.0000271399<br>1.4015 ± 0.0000414854
m = 9	1.40124 ± 0.0000278606<br>1.40115 ± 0.0000347126<br>1.40134 ± 0.000027668<br>1.40125 ± 0.0000345592	1.40124 ± 0.0000277728<br>1.40117 ± 0.0000334189<br>1.40133 ± 0.0000275976<br>1.40126 ± 0.0000332742
m = 10	1.40132 ± 0.0000255637<br>1.40132 ± 0.000264139<br>1.40165 ± 0.0000399625<br>1.40165 ± 0.000265979	1.40133 ± 0.0000254363<br>1.40108 ± 0.0000494259<br>1.40164 ± 0.000040378<br>1.40138 ± 0.0000585383
m = 11	1.40025 ± 0.0000257106<br>1.4007 ± 0.0000257145<br>1.40082 ± 0.0000318311<br>1.40127 ± 0.0000318378	1.40021 ± 0.0000203679<br>1.40067 ± 0.0000249593<br>1.40077 ± 0.0000282644<br>1.40122 ± 0.0000317397

Systematic Error
Statistical
0.000349159<br>0.000298894<br>0.00027212<br>0.00013785

m	Statistical	CDF
m = 1	1.40107 ± 0.000023<br>1.40106 ± 0.000032<br>1.4014 ± 0.00002	1.40108 ± 0.000022<br>1.40107 ± 0.000031<br>1.4014 ± 0.00002

m	Statistical	CDF
	1.4014 ± 0.00002<br>1.40139 ± 0.00003	1.4014 ± 0.00002<br>1.40139 ± 0.00003
m = 2	1.40108 ± 0.000023<br>1.40102 ± 0.000031<br>1.40136 ± 0.00002<br>1.40129 ± 0.000029	1.4011 ± 0.000022<br>1.40103 ± 0.00003<br>1.40136 ± 0.00002<br>1.4013 ± 0.000029
m = 3	1.40108 ± 0.000021<br>1.40086 ± 0.000025<br>1.4018 ± 0.00002<br>1.40157 ± 0.000024	1.4011 ± 0.00002<br>1.40087 ± 0.000025<br>1.4018 ± 0.00002<br>1.40157 ± 0.000024
m = 4	1.40039 ± 0.000023<br>1.40031 ± 0.000053<br>1.40131 ± 0.000026<br>1.40124 ± 0.000055	1.40039 ± 0.000023<br>1.40023 ± 0.000052<br>1.40133 ± 0.000026<br>1.40117 ± 0.000054
m = 5	1.40124 ± 0.000028<br>1.40135 ± 0.000033<br>1.40125 ± 0.000029<br>1.40136 ± 0.000034	1.40125 ± 0.000027<br>1.40135 ± 0.000033<br>1.40125 ± 0.000027<br>1.40135 ± 0.000033
m = 6	1.40098 ± 0.000018<br>1.40119 ± 0.000031<br>1.40106 ± 0.000018<br>1.40128 ± 0.000031	1.40096 ± 0.000017<br>1.4012 ± 0.000033<br>1.40104 ± 0.000017<br>1.40127 ± 0.000033
m = 7	1.40113 ± 0.000018<br>1.40116 ± 0.000025<br>1.40137 ± 0.000019<br>1.4014 ± 0.000025	1.40111 ± 0.000017<br>1.40116 ± 0.000026<br>1.40135 ± 0.000018<br>1.4014 ± 0.000026
m = 8	1.40118 ± 0.000028<br>1.40106 ± 0.000042<br>1.40162 ± 0.000027<br>1.4015 ± 0.000042	1.40118 ± 0.000028<br>1.40107 ± 0.000042<br>1.40162 ± 0.000027<br>1.4015 ± 0.000041
m = 9	1.40124 ± 0.000028<br>1.40115 ± 0.000035<br>1.40134 ± 0.000028<br>1.40125 ± 0.000035	1.40124 ± 0.000028<br>1.40117 ± 0.000033<br>1.40133 ± 0.000028<br>1.40126 ± 0.000033
m = 10	1.40132 ± 0.000026<br>1.40132 ± 0.00026<br>1.40165 ± 0.00004<br>1.40165 ± 0.00027	1.40133 ± 0.000025<br>1.40108 ± 0.000049<br>1.40164 ± 0.00004<br>1.40138 ± 0.000059
m = 11	1.40025 ± 0.000026<br>1.4007 ± 0.000026<br>1.40082 ± 0.000032<br>1.40127 ± 0.000032	1.40021 ± 0.00002<br>1.40067 ± 0.000025<br>1.40077 ± 0.000028<br>1.40122 ± 0.000032

Systematic Error
CDF
0.000359551
0.000308335
0.00028227
0.000119998

```
m = 4; Print@{
  {
    MapThread[{#1, Around[#2, #3]} &,
      {
       {1, 1, 2, 3, 3, 4},
       #[[1]] & /@ JcoupRatioStat[k, m, 8],
       #[[2]] & /@ JcoupRatioStat[k, m, 8]
      }
     ][[{2, 3, 5, 6}]],
    MapThread[{#1, Around[#2, #3]} &,
      {
       {1, 1, 2, 3, 3, 4},
       #[[1]] & /@ JcoupRatioCDF[k, m, 8],
       #[[3]] & /@ JcoupRatioCDF[k, m, 8]
      }
     ][[{2, 3, 5, 6}]]
   } // Quiet // Column, ,
  {
    MapThread[{#1, Around[#2, #3]} &,
      {
       {1, 1, 2, 3, 3, 4},
       #[[1]] & /@ JcoupRatioSys[1][k, m, 8],
       #[[2]] & /@ JcoupRatioSys[1][k, m, 8]
      }
     ][[{2, 3, 5, 6}]],
    MapThread[{#1, Around[#2, #3]} &,
      {
       {1, 1, 2, 3, 3, 4},
       #[[1]] & /@ JcoupRatioSys[2][k, m, 8],
       #[[2]] & /@ JcoupRatioSys[2][k, m, 8]
      }
     ][[{2, 3, 5, 6}]]
   } // Quiet // Column, ,
 } // Column;
```

```
{ {{1, 1.400388 ± 0.000023}, {2, 1.40031 ± 0.00005}, {3, 1.401313 ± 0.000026}, {4, 1.40124 ± 0.00005}}
  {{1, 1.400389 ± 0.000023}, {2, 1.40023 ± 0.00005}, {3, 1.401326 ± 0.000026}, {4, 1.40117 ± 0.00005}} , Null,
  {{1, 1.40039 ± 0.00035}, {2, 1.40031 ± 0.00030}, {3, 1.40131 ± 0.00027}, {4, 1.40124 ± 0.00014}}
  {{1, 1.4004 ± 0.0004}, {2, 1.40023 ± 0.00031}, {3, 1.40133 ± 0.00028}, {4, 1.40117 ± 0.00012}} , Null, Null }
```

2/22/24 14:54:26 In[]:=

```
Table[
  Export[FileNameJoin[{datafolderMain, "Answer to Reviewers v2",
     "Fig8ZoomOut[k," <> ToString[m] <> "," <> ToString[nmax] <> "].pdf"}],
   Fig8ZoomOut[k, m, nmax], ImageSize → Large];
  Export[FileNameJoin[{datafolderMain, "Answer to Reviewers v2", "Fig8[k," <> ToString[m] <>
      "," <> ToString[nmax] <> "].pdf"}], Fig8[k, m, nmax], ImageSize → Large];
  , {m, mmaxTot}];
```

2/22/24 14:54:34 In[]:=

```
Export[FileNameJoin[
   {datafolderMain, "Answer to Reviewers v2", "Fig8ZoomOut[k," <> ToString[nmax] <> "].gif"}],
  Table[Fig8ZoomOut[k, m, nmax], {m, mmaxTot}], "DisplayDurations" → 0.5];
Export[FileNameJoin[
   {datafolderMain, "Answer to Reviewers v2", "Fig8[k," <> ToString[nmax] <> "].gif"}],
  Table[Fig8[k, m, nmax], {m, mmaxTot}], "DisplayDurations" → 0.5];
```

2/22/24 14:54:41 In[]:=

```
Export[FileNameJoin[{datafolderMain, "Answer to Reviewers v2",
    "Fig8[k,m=" <> ToString@Range@mmaxTot <> "," <> ToString[nmax] <> "].pdf"}],
  Table[Fig8[k, m, nmax], {m, mmaxTot}] // Column, ImageSize → Large];
Export[FileNameJoin[{datafolderMain, "Answer to Reviewers v2",
    "Fig8ZoomOut[k,m=" <> ToString@Range@mmaxTot <> "," <> ToString[nmax] <> "].pdf"}],
  Table[Fig8ZoomOut[k, m, nmax], {m, mmaxTot}] // Column, ImageSize → Large];
```
