# Supplementary material for: Zero-field J-spectroscopy of quadrupolar nuclei
Source: Nat Commun. 2024 May 27;15:4487. doi: 10.1038/s41467-024-48390-2 (PMC11637023; doi:10.1038/s41467-024-48390-2)

Simulation of $^{15}NH_3$ D_1 at zero field

Preinitialization

10/19/23 15:52:33 In[]:=

```
SetOptions[ListLinePlot, Axes → False, Frame → True, PlotRange → All,
  AspectRatio → 0.4, BaseStyle → 13, ImageSize → Medium, PlotStyle → RGBColor[0, 0, 1]];
SetOptions[ListPlot, Axes → False, Frame → True, PlotRange → All,
  AspectRatio → 0.4, BaseStyle → 13, ImageSize → Medium, PlotStyle → RGBColor[0, 0, 1]];
```

Fourier transform

10/19/23 15:52:33 In[]:=

```
fourpos[data_, dt_] := Module[{nerp},
  ft = 2 / Sqrt[Length[data]] Fourier[data];
  ft = Drop[ft, -Length[data] / 2];
  nuvals = Table[1 / (Length[data] * dt) * (j - 1), {j, 1, Length[data] / 2}];
  ft = Transpose[{nuvals, ft}]
 ](*Fourier tranform of the data*)
```

10/19/23 15:52:33 In[]:=

```
cullkeepx[data_, νrange_] := Module[{jmin, jmax, dν, dum},
  dν = data⟦2, 1⟧ - data⟦1, 1⟧;
  dum = Drop[data, -(Length[data] - Floor[νrange⟦2⟧ / dν])];
  Drop[dum, Floor[νrange⟦1⟧ / dν]]](*Construct the frequency axis*)
```

Physical Properties

10/19/23 15:52:33 In[]:=

```
M = 6 mol / L ;(*Molar concentration of the compound*)
NMoleculesPerLitre = M * 6.02 × 10^23 / mol;(*Number of molecues per litre*)
SampleVolume = 0.0003 L;

kB = 1.380649 * 10^-23;(* J / K *)
T = 300; (*K*)
kT = kB * T;
hbar = 1.054571817 * 10^-34; (* J s *)

γH = 2 Pi * 42.57746905766274; (* rad /(s uT) *)
γD = 2 Pi * 6.5359013131563915;(* rad /(s uT) *)
γN14 = 2 Pi * 3.0777051852829093;(* rad /(s uT) *)
γN15 = 2 Pi * -4.317265697862487;(* rad /(s uT) *)
```

Simulation

Simulation Parameters

10/19/23 15:52:33 In[]:=

```
(*  ZULF Magnetic field in uT*)
Bx := 0.0000;
By := 0.0000;
Bz := 0.0000;
Bp = 2*10^6; (* Prepolarizing Field in uT*)
```

10/19/23 15:52:33 In[]:=

```
dt = 0.001; (*Time increment, or 1/(sampling rate)*)
LW = 2; (*full width at half maximum in Hz*)
T2 = 1/(π LW); (*Coherence decay time*)
acq = 20; (*Acquisition time in s*)

Spins = {1/2, 1/2, 1/2, 1/2, 1}; (* Here we input the spin number of each nuclei,
where the nitrogen is in the first position *)
gamma = {γN15, γH, γH, γH, γD};
NspinsOneHalf = Length@Select[Spins, # == 1/2 &];
(* We count the number of spins that are 1/2 *)
NspinsOne = Length@Select[Spins, # == 1 &]; (* We count the number of spins that are 1 *)
Nspins = NspinsOne + NspinsOneHalf; (* Those are the only possibilities,
so the sum is the total number of spins*)
```

10/19/23 15:52:33 In[]:=

```
(* J-Couplings in Hz*)
JNH := +73.429;
JND := +11.3;
JHD := +2.6;

 (*J-Coupling Topology*)
jnet = Table[0, {i, 1, Nspins}, {j, 1, Nspins}];

jnet[[1, 2]] = jnet[[1, 3]]  = jnet[[1, 4]] = JNH ;
jnet[[1, 5]] = JND ;
jnet[[2, 5]]  = jnet[[3, 5]] = jnet[[4, 5]] = JHD ;

jnet // MatrixForm
```

10/19/23 15:52:33 Out[]//MatrixForm=

$$\begin{pmatrix} 0 & 73.429 & 73.429 & 73.429 & 11.3 \\ 0 & 0 & 0 & 0 & 2.6 \\ 0 & 0 & 0 & 0 & 2.6 \\ 0 & 0 & 0 & 0 & 2.6 \\ 0 & 0 & 0 & 0 & 0 \end{pmatrix}$$

Spin operators

10/19/23 15:52:33 In[]:=

```
imax = 2^NspinsOneHalf * 3^NspinsOne; (*We construct the Hilbert space*)
```

```
σ = {{{1, 0}, {0, 1}}, 1/2 {{0, 1}, {1, 0}}, 1/2 {{0, -I}, {I, 0}}, 1/2 {{1, 0}, {0, -1}},
   {{1, 0, 0}, {0, 1, 0}, {0, 0, 1}}, √2/2 {{0, 1, 0}, {1, 0, 1}, {0, 1, 0}},
   √2/(2 I) {{0, 1, 0}, {-1, 0, 1}, {0, -1, 0}}, {{1, 0, 0}, {0, 0, 0}, {0, 0, -1}}};
(*Sets of Pauli Matrices for spin 1/2 and spin 1*)

ZeemanBasis := Module[{ner},
  MzQuantumNumbers = {{0, 0}, {0, 0}};
  For[i = 1, i ≤ Nspins, i++,
   tmp = Table[s, {s, Spins[[i]], -Spins[[i]], -1}];
   MzQuantumNumbers = Insert[MzQuantumNumbers, tmp, 2 + i];
  ];
  MzQuantumNumbers = Drop[MzQuantumNumbers, 2];
  basis = Tuples[MzQuantumNumbers];
  basis] (* This is the uncoupled Zeeman basis *)

IOps = ConstantArray[0, {Nspins, 3}];
For[i = 1, i ≤ Nspins, i++,
  For[j = 1, j ≤ 3, j++,
   list1 = Table[KroneckerDelta[s, i], {s, 1, Nspins}];
   Which[Max[list1 * Spins] == 1/2,
    list1 = (j + 1) * list1,
    Max[list1 * Spins] == 1,
    list1 = (j + 5) * list1
   ];
   list2 = (ConstantArray[1, {Nspins}] - Table[KroneckerDelta[s, i], {s, 1, Nspins}]) * Spins;
   For[k = 1, k ≤ Nspins, k++,
    Which[list2[[k]] == 1/2,
      list2[[k]] = 1,
      list2[[k]] == 1,
      list2[[k]] = 5
     ];];
   list1 = list1 + list2;
   IOps[[i, j]] = σ[[list1[[1]]]];
   If[Nspins > 1,
    For[k = 2, k ≤ Nspins, k++,
      IOps[[i, j]] = KroneckerProduct[IOps[[i, j]], σ[[list1[[k]]]]];
     ];];];]];(*We construct the spin operators
 taking into acount indvidual spin being either 1/2 or 1*)
```

Hamiltonians

```
Hj := 2 π * Sum[ jnet[[i, j]] (IOps[[i, 1]].IOps[[j, 1]] + IOps[[i, 2]].IOps[[j, 2]] + IOps[[i, 3]].IOps[[j, 3]]),
    {i, 1, Nspins}, {j, 1, Nspins}];(*Zero-field hamiltonian,
where 1,2,3 represent the x, y, and z axis, respectively*)
Hz := - (Bx * Sum[gamma[[i]] × IOps[[i, 1]], {i, 1, Nspins}] +
     By * Sum[gamma[[i]] × IOps[[i, 2]], {i, 1, Nspins}] + Bz * Sum[gamma[[i]] × IOps[[i, 3]], {i, 1, Nspins}]);
(*Zeeman hamiltonian*)
```

Observable and propagation

10/19/23 15:52:33 In[]:=

```
(*We observe along the z-axis of total magnetization observable*)
Mutotz = Sum[gamma⟦j⟧ × IOps⟦j, 3⟧, {j, 1, Nspins}];
```

10/19/23 15:52:33 In[]:=

```
(* Density matrix elements in the basis of eigenstates of the hamiltonian *)
Rho[ρ_, eigensystem_, a_, b_] := Conjugate[eigensystem⟦2, a⟧].ρ.eigensystem⟦2, b⟧;
```

10/19/23 15:52:33 In[]:=

```
(* Time dependent magnetization given initial density matrix ρ and eigensystem *)
Magzt[ρ_, eigensystem_, t_] :=
 Parallelize[Sum[Exp[-I (Chop[eigensystem⟦1, a⟧ - eigensystem⟦1, b⟧]) t]
      Chop[Rho[ρ, eigensystem, a, b] * Conjugate[eigensystem⟦2, b⟧].Mutotz.eigensystem⟦2, a⟧],
     {a, 1, imax}, {b, 1, imax}]] // ExpToTrig // Chop
```

10/19/23 15:52:33 In[]:=

```
Propagate[rho_, H_, t_] := Module [{v},
  v = Transpose[H⟦2, All⟧];
  U[t] = v.DiagonalMatrix[Exp[-I (H⟦1, All⟧) t]].Inverse[v];
  Rho[t] = Chop[U[t].(rho ).ConjugateTranspose[U[t]]];
  Rho[t]
 ]
```

Calculate the eigen system of the Hamiltonian

10/19/23 15:52:33 In[]:=

```
(* Eigenstates and eigenvalues *)
esys = Eigensystem[Chop[Hj + Hz]];
Do[esys⟦2, i⟧ = esys⟦2, i⟧ / Norm[esys⟦2, i⟧], {i, 1, Length[esys]}]
```

10/19/23 15:52:34 In[]:=

```
Table[Conjugate[esys⟦2, i⟧].esys⟦2, j⟧,
    {i, 1, Length[esys⟦1⟧]}, {j, 1, Length[esys⟦1⟧]}] // Chop // MatrixForm;
```

Thermal polarization, pulse, evolve

10/19/23 15:52:34 In[]:=

```
beta = hbar Bp gamma / kT; (* For thermal polarization *)
Z = Sum[beta⟦i⟧ * IOps⟦i, 3⟧, {i, 1, Nspins}]; (*Partition function*)
rho0 = MatrixExp[Z] / Tr[MatrixExp[Z]]; (*thermal distribution density operator*)
```

10/19/23 15:52:34 In[]:=

```
(* Generate observable *)
mzSudden[t_] = Magzt[rho0, esys, t];

(* Conversion to moles *)
NMoleculesPerSample = SampleVolume * NMoleculesPerLitre;
```

10/19/23 15:52:34 In[]:=

```
ts = 0;
mzvalsSudden = Re[Chop[Parallelize[Table[mzSudden[t] Exp[-t / T2], {t, ts, acq - dt, dt}]]] *
    NMoleculesPerSample * hbar * 10^6 * 10^12 ];
tvals = Parallelize[Table[t, {t, ts, acq - dt, dt}]]; (*We parallelize to compute faster*)
```

10/19/23 15:52:39 In[]:=

```
parsSudden = FindFit[Transpose[{tvals, Chop[mzvalsSudden]}], A*Exp[-t/T2], {{A, -1}}, t];
(*Subtraction of DC magnetization*)
mzvalsSudden = mzvalsSudden - A*Exp[-tvals/T2] /. parsSudden;
ListLinePlot[{Transpose[{tvals, mzvalsSudden}]},
 PlotRange → All, FrameLabel → {"Time (s)", "Signal (pT)"}]
```

10/19/23 15:52:39 Out[]=

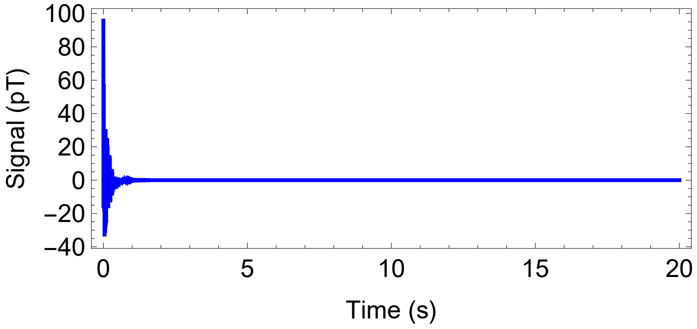

Get the spectra

10/19/23 15:52:40 In[]:=

```
SpecSuddenNH3D1 = fourpos[mzvalsSudden, dt];
SpecSuddenNH3D1 = cullkeepx[SpecSuddenNH3D1, {0, 200}];
pdrsim4 = ListLinePlot[{#[[1]], Re@#[[2]]/0.15} & /@ SpecSuddenNH3D1,
  FrameLabel → {"Frequency (Hz)", "Signal (norm.)"}]
```

10/19/23 15:52:40 Out[]=

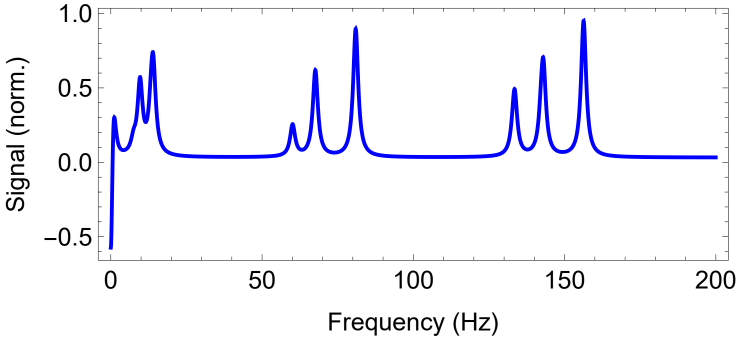

Supplement: Supplementary file 5 — Supplementary Data 2 [file 41467_2024_48390_MOESM5_ESM.pdf]
